# The subthalamic nucleus-ventral pallidum projection targeting cholinergic circuits modulates chronic pain

**DOI:** 10.1371/journal.pbio.3003923

**Published:** 2026-08-03

**Authors:** Ya-Wei Ji, Xiang-Ying Xu, Qiang-Qiang Zhou, Yin-Nan Cao, Xing-Jie Zhao, Kai-Ran Zhang, Yu Han, Su Liu, Yong Niu, Chun Yang, Chunyi Zhou, Cheng Xiao

**Affiliations:** 1 Department of Anesthesiology and Perioperative Medicine, The First Affiliated Hospital of Nanjing Medical University, Nanjing, Jiangsu, China; 2 Jiangsu Province Key Laboratory of Anesthesiology, School of Anesthesiology, Xuzhou Medical University, Xuzhou, Jiangsu, China; 3 Jiangsu Province Key Laboratory of Anesthesiology and Brain Science, Xuzhou Medical University, Xuzhou, Jiangsu, China; 4 Department of Anesthesiology, Affiliated Hospital of Xuzhou Medical University, Xuzhou, Jiangsu, China; 5 Key Laboratory of Chemical Safety and Health, National Institute for Occupational Health and Poison Control, Chinese Center for Disease Control and Prevention, Beijing, China; Columbia University Irving Medical Center, UNITED STATES OF AMERICA

## Abstract

The cholinergic system shows promise for pain management. Although cholinergic neurons in the ventral pallidum (VP) are involved in neuropathic pain, the anatomical organization and pharmacology of the cholinergic circuit contributing to the processing of acute and chronic pain remain unknown. In the present study, we demonstrate that VP cholinergic neurons in mice received pain signals from and mediated pain modulation by glutamatergic (Glu) neurons in the subthalamic nucleus (STN) in both physiological and neuropathic pain conditions. VP cholinergic neurons were a major source of acetylcholine released in the VP upon pain stimulation, and α4 and α7 were major subunits comprising functional nicotinic acetylcholine receptors (nAChRs) in VP cholinergic, GABAergic, and Glu neurons. In neuropathic pain mouse models, VP cholinergic neurons became hyperactive and released more acetylcholine in the VP, and α4 and α7 nAChRs were differentially modified on VP GABAergic and Glu neurons. These modifications were accompanied by hyperexcitability in VP GABAergic neurons and hypoactivity in VP Glu neurons. Reversal of upregulated α4 nAChRs on VP GABAergic neurons, but not that of downregulated α4 and α7 nAChRs on VP Glu neurons, attenuated the severity of hyperalgesia in neuropathic pain. Therefore, the STN^Glu^-VP^ChAT^ pathway is implicated in neuropathic pain by enhancing VP cholinergic circuits, and α4 nAChRs on VP GABAergic neurons may be potential therapeutic targets for the treatment of neuropathic pain.

## Abstract 2

Cholinergic circuits in the ventral pallidum contribute to pain processing, but their organization and receptor mechanisms in acute and neuropathic pain remain unclear. This study shows that subthalamic glutamatergic inputs drive cholinergic activity, with α4-containing receptors on GABAergic neurons mediating hyperalgesia.

## Introduction

Chronic pain is a major public health problem. Approximately 10%−15% of global population has been suffered from debilitating chronic pain with seriously degraded quality of life [1,2]. In contrast, the currently available therapies with opioid and non-steroid anti-inflammatory drugs fail to meet the demand for controlling symptoms of chronic pain without causing intolerable side effects [3–5]. Validation of effective therapeutic targets for pain relief is highly needed for drug development to overcome this challenge.

The cholinergic system may be a promising target for drug development to relieve pain [6–9]. Systemic enhancement of cholinergic transmission with pharmacological means mitigates hyperalgesia or spontaneous pain in many pain states, including inflammatory pain, migraine, and neuropathic pain [7,9–11]. However, the heterogeneity in cholinergic receptor subtypes, locations, and physiological outcomes prompts that more specific strategies are needed to find precise targets to minimize side effects of cholinergic drugs currently used for pain relief. To fulfill this purpose, it is keenly important to dissect endogenous cholinergic circuits in pain modulation.

Cholinergic neurons reside in forebrain and brain stem nuclei, including the diagonal band of Broca (DB), nucleus basalis of Meynert (NBM), medial septum, substantia innominata, ventral pallidum (VP), and pedunculopontine tegmental nucleus (PPN) [5,12–14], and distinctly modulate pain. Ablation of basal forebrain cholinergic neurons with intraventricular injection of 192-IgG-saporin reduces sensitivity to thermal stimulation in rats [15]. Recent cell- and projection-specific neuromodulation studies reveal that the role of cholinergic neurons in pain modulation depends on their projections. For instance, cholinergic in the medial septum exert anti- and pro-nociceptive effects through their projections to the ventral hippocampus and anterior cingulate cortex, respectively [16]. NBM cholinergic neurons alleviates inflammatory pain through their projections to the prelimbic cortex [17], while inhibition of cholinergic neurons in the anterior nucleus basalis relieves neuropathic pain during sleep through their projections to the primary sensorimotor cortex [18]. The cholinergic projection from the PPN to the ventrolateral periaqueductal gray elevated pain thresholds even in morphine-tolerant condition [8]. Inhibition of VP cholinergic neurons mitigates inflammatory and neuropathic pain through their projections to VP non-cholinergic neurons and to neurons in the basolateral amygdala [19], a region involved in hyperalgesia [20]. Stimulation of projections from the PPN to the substantia nigra pars reticulata relieved hyperalgesia in inflammatory and neuropathic pain [21]. Therefore, cholinergic neurons in different nuclei may mitigate or exacerbate pain through distinct neural pathways. Addressing subtypes and locations (such as nuclei, neuron types, etc.) of acetylcholine receptors mediating the function of cholinergic neurons is important to reveal potential targets for the development of analgesic drugs.

Our previous study shows that stimulating synaptic inputs to the VP from the subthalamic nucleus (STN) reduces pain thresholds in mice, while inhibition of the STN-VP projection ameliorates hyperalgesia in parkinsonian mice [22]. Additionally, both STN neurons and VP cholinergic neurons respond to pain-like stimulation with enhanced activity [19,23]. These findings suggest that VP cholinergic neurons may be synaptic targets of STN glutamatergic neurons in pain modulation. But this possibility should be addressed with cell-specific circuit interrogations.

In the present study, we demonstrated that pain signaling and modulation by VP cholinergic neurons were monosynaptically controlled by STN glutamatergic neurons. Major nAChRs on glutamatergic and GABAergic neurons in the VP contained α4 and α7 subunits, which were differentially modified in neuropathic pain; reversal of the modification of α4 nAChRs on GABAergic neurons, but not α4 and α7 subunits on the glutamatergic neurons, mitigates hyperalgesia in neuropathic pain. Therefore, STN^Glu^-VP^ChAT^ circuit can be intervened by targeting α4 nAChRs on VP GABAergic neurons to treat chronic neuropathic pain.

## Results

### The subthalamic projection to VP cholinergic neurons signals and modulates pain

Recent studies reveal that individually stimulating STN neurons, the STN-VP projection, and VP cholinergic neurons reduces pain thresholds [19,22,23]. Because GABAergic, glutamatergic, and cholinergic neurons account for about 70%, 15%, and 10% of neurons in the VP, respectively [24,25], it remains unknown whether VP cholinergic neurons are among major contributors to mediate hyperalgesia induced by stimulation of the STN-VP projection [22]. To address this issue, we first used a cell-specific retrograde tracing strategy involving rabies virus to probe the upstream nuclei of VP cholinergic neurons. We injected AAV-EF1α-DIO-TVA-eGFP and AAV-EF1α-DIO-oRVG into the VP of ChAT-Cre mice, and 2 weeks later, injected RV-CVS-EnvA(∆G)-tdTomato into the VP, then the mice were allowed to recover for 7–10 days (S1A and S1B Fig). Histological assay shows that the STN is among upstream nuclei of VP cholinergic neurons, in addition to the nucleus accumbens, medial prefrontal cortex, and periventricular, mediodorsal, and parafascicular nuclei of the thalamus, etc. (S1C–S1E Fig). These results indicate that VP cholinergic neurons are innervated by the STN-VP projection.

**Fig 1:**
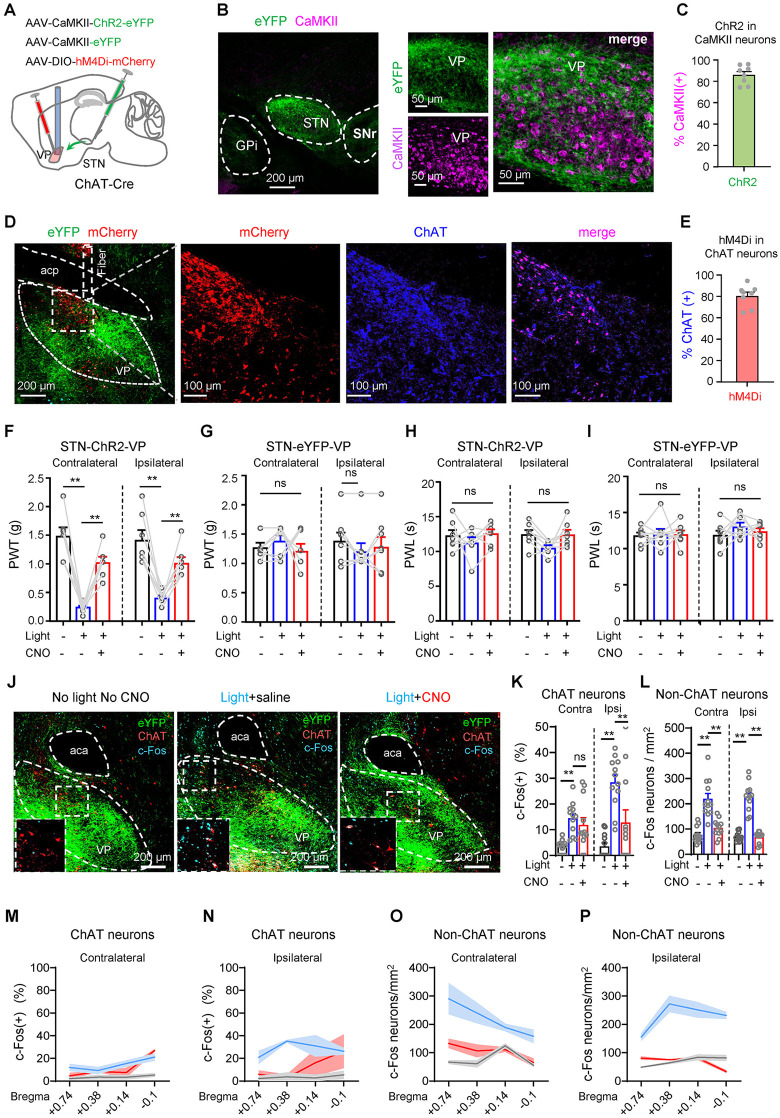
VP cholinergic neurons mediate hypersensitivity induced by optogenetic stimulation of the STN-VP projection. **(A)** Schematic diagram for injecting AAV-CaMKII-ChR2-eYFP or AAV-CaMKII-eYFP into the STN and AAV-EF1α-DIO-hM4Di-mCherry into the VP, and implanting an optical fiber into the VP in ChAT-Cre mice. **(B, C)** Representative images and summary showing eYFP expression in the STN glutamatergic neurons. CaMKII-ChR2 overlapping neurons accounted for 86% of ChR2 neurons. Summary data were from 8 slices of 4 mice. **(D, E)** Representative images and summary showing mCherry in the VP cholinergic neurons. ChAT-hM4Di overlapping neurons accounted for 80.5% of hM4Di neurons. Summary data were from 8 slices of 4 mice. **(F**–**I)** Mechanical paw withdrawal threshold (PWT) and thermal paw withdrawal latency (PWL) on either hind paw before and during blue light illumination of the VP following intraperitoneal administration of saline or CNO (3 mg/kg) in both ChR2 (*n* = 7) and eYFP (*n* = 8) mice. **(F)** PWT in ChR2 mice. Contralateral: *F*_(1.515, 9.091)_ = 32.46, *P* = 0.001; *t* = 7.81, *P* < 0.001, control vs. light; *t* = 7.16, *P* < 0.001, ligh*t* vs. light+CNO. Ipsilateral: *F*_(1.527, 9.164)_ = 25.10, *P* < 0.001; *t* = 5.66, *P* = 0.0026, control vs. light; *t* = 5.31, *P* = 0.0026, light vs. light+CNO. **(G)** PWT in eYFP mice. Con*t*ralateral: *F*_(1.209, 8.465)_ = 0.54, *P* = 0.51. Ipsi: *F*_(1.815, 12.71)_ = 1.45, *P* = 0.27. **(H)** PWL in ChR2 mice. Contralateral: *F*_(1.408, 8.451)_ = 0.96, *P* = 0.39. Ipsi: *F*_(1.105, 6.629)_ = 3.38, *P* = 0.11. **(I)** PWL in eYFP mice. Contralateral: *F*_(1.644, 11.51)_ = 0.029, *P* = 0.95. Ipsilateral: *F*_(1.724, 12.07)_ = 1.30, *P* = 0.30. **(J**–**L)** Representative images and summary for optogenetic stimulation of the STN-VP projection (green)-induced changes in c-Fos-positive (cyan) cholinergic (red) and non-cholinergic neurons in the VP on either side with and without intraperitoneal administration of CNO (3 mg/kg). Insets show cholinergic (red) and c-Fos-positive neurons (cyan). Summary data were from 8 slices of 4 mice in each group. **(K)** ChAT c-Fos (%). Contralateral, *F*_(2, 33)_ = 8.14, *P* = 0.001; *t* = 3.86, *P* = 0.001, Control vs. Light; *t* = 0.92, *P* = 0.36, Light vs. Light + CNO; Ipsila*t*eral: *F*_(2, 33)_ = 13.86, *P* < 0.001; *t* = 5.21, *P* < 0.001, Control vs. Light; *t* = 3.27, *P* = 0.002, Ligh*t* vs. Light+CNO. **(L)** Non-ChAT c-Fos (%). Contralateral, *F*_(2, 33)_ = 27.3, *P* < 0.001; *t* = 6.96, *P* < 0.001, Control vs. Light; *t* = 5.63, *P* < 0.001, Light vs. Light+CNO. Ipsila*t*eral, *F*_(2, 33)_ = 78.23, *P* < 0.0001; *t* = 10.76, *P* < 0.001, Control vs. Light; *t* = 10.9, *P* < 0.001, Light vs. Light+CNO. **(M–P)** Numbers of c-Fos+ neurons at anterior-posterior levels (rela*t*ive to bregma) of coronal sections before (gray) and after op*t*ogenetic stimulation of the STN-VP projection (blue) and optogenetic stimulation + intraperitoneal administration of CNO (red). ** *P* < 0.01; ns not significant. One-way Repeated Measures ANOVAs with Bonferroni tests for **(F**–**I)**. One-way ANOVAs for **(K, L)**. Contra, Contralateral; Ipsi, Ipsilateral. Data are available in S1 Data as a part of Supporting information.

To confirm the functionality of the STN-VP projection onto VP cholinergic neurons, we injected AAV-CaMKII-ChR2-eYFP into the right STN and AAV-EF1α-DIO-hM4Di-mCherry into the right VP in ChAT-Cre mice (Fig 1A and 1D). These viral vectors labeled VP cholinergic neurons and STN CaMKII neurons with a high specificity (Fig 1B–1E). Six weeks later, we performed patch-clamp recording from mCherry-labeled VP cholinergic neurons in live brain slices (S2A–S2C Fig). Optogenetic stimulation of ChR2-labeled STN-VP projection evoked excitatory postsynaptic currents (EPSCs) in mCherry-labeled VP cholinergic neurons. The EPSCs were not sensitive to TTX (1 μM) + 4-AP (0.1 mM), but were eliminated by CNQX (20 μM), an AMPA receptor antagonist (S2D–S2F Fig). Additionally, the STN-VP projection faithfully followed optogenetic stimulation up to 20 Hz (460 nm, 5 ms, 2 mW) (S2G and S2H Fig). These data suggest that VP cholinergic neurons receive functional mono-synaptic glutamatergic (Glu) projections from STN CaMKII neurons.

To address whether VP cholinergic neurons mediate modulation of mechanical and thermal thresholds by the STN-VP projection, we combined optogenetic stimulation of the STN-VP projection and chemogenetic inhibition of VP cholinergic neurons as illustrated in Fig 1A. We observed that 3 min bilateral blue light illumination (5 ms, 20 Hz, 21.72 mW/mm^2^) of the STN-VP projection increased distance and velocity in the open field tests (OFT) in ChR2 mice, but not in eYFP mice; it did not change time in the central zone in the OFT (S3A–S3E Fig); in contrast, 3 min unilateral blue light illumination (5 ms, 20 Hz, 21.72 mW/mm^2^) of the STN-VP projection did not change distance and velocity in the OFT, and center time and rotation in the OFT in both ChR2 and eYFP mice (S3F–S3H Fig). Therefore, unilateral stimulation of the STN-VP projection has minor effects on motor function, and may suitable for examining the modulation of pain thresholds by the STN-VP projection.

In pain-like behavioral tests, we observed that unilateral optogenetic stimulation (5 ms, 20 Hz, 21.72 mW/mm^2^) of the STN^Glu^-VP projection reduced paw withdrawal threshold (PWT) but not thermal paw withdrawal latency (PWL) on either hind paw (Fig 1F–1I); chemogenetic inhibition of VP cholinergic neurons by intraperitoneal injection of clozapine-N-oxide (CNO, 3 mg/kg) dramatically attenuated the reduction of mechanical PWT and thermal PWL induced by optogenetic stimulation of the STN^Glu^-VP projection (Fig 1F–1I). After optogenetic stimulation of the STN^Glu^-VP projection (5 ms, 20 Hz, 21.72 mW/mm^2^, 2 min episodes with 2 min intervals for 30 min), we observed increased c-Fos-positive neurons in the VP, including both cholinergic and non-cholinergic neurons, while chemogenetic inhibition of VP cholinergic neurons attenuated these effects (Fig 1J–1P). These results suggest that VP cholinergic neurons play important roles in mediating mechanical and thermal sensitization and enhancement of activity in non-cholinergic neurons by stimulation of the STN^Glu^-VP projection.

**Fig 2:**
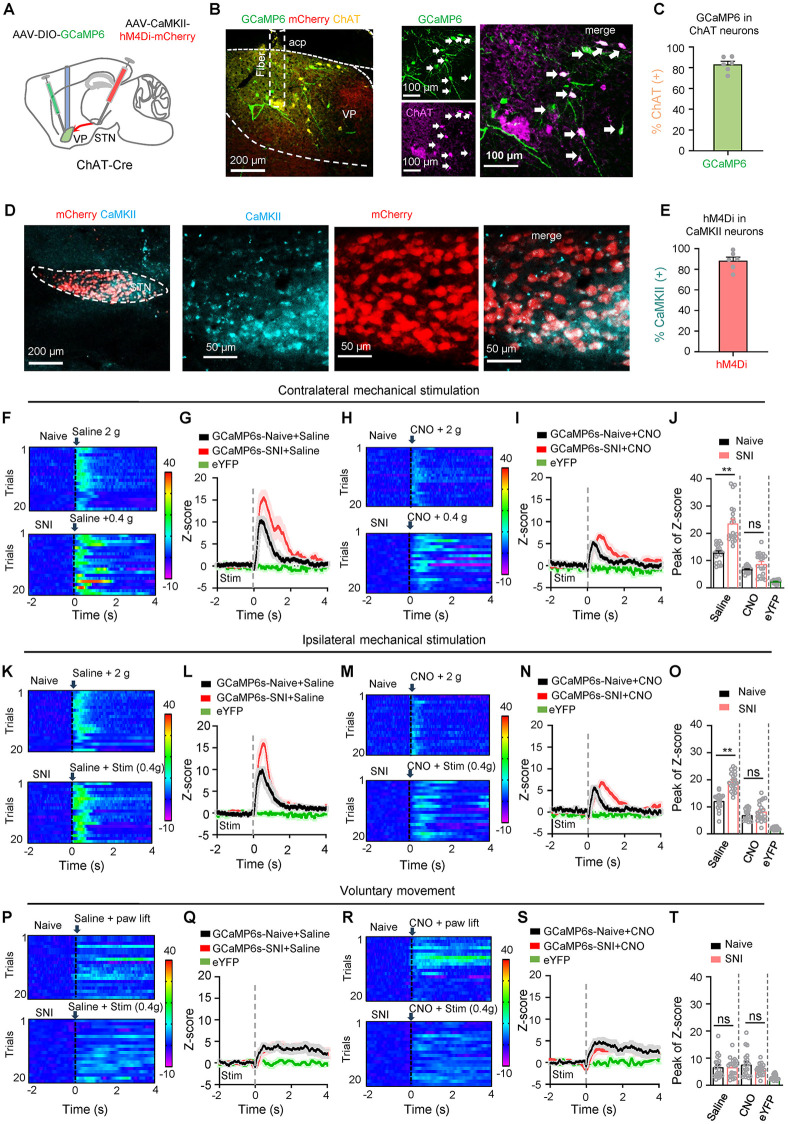
Subthalamic neurons control mechanical responses in VP cholinergic neurons. **(A)** Schematic diagram and a representative image for injecting AAV-EF1α-DIO-GCaMP6s or AAV-EF1α-DIO-eYFP into the VP and AAV-CaMKII-hM4Di-mCherry into the subthalamic nucleus (STN), and implanting optical fiber into the VP in ChAT-Cre mice. **(B, C)** A representative image showing the overlapping of GCaMP6 and ChAT-antibody-labeled neurons. ChAT-GCaMP6 overlapping neurons accounted for 83% of GCaMP6 neurons. Data from 6 slices (2 from each mouse) were summarized. **(D, E)** Representative images and summary showing hM4Di and CaMKII-antibody-labeled neurons in the STN. Data from 6 slices (2 from each mouse) were summarized. Eighty-eight% of hM4Di neurons expressed CaMKII. **(F**–**J)** Heat maps, averaged traces, and summary showing responses of GCaMP6 signal to mechanical stimulation on the contralateral hind paw in GCaMP6-Naive (*n* = 6), GCaMP6-SNI (*n* = 6), and eYFP (*n* = 6) mice before and after intraperitoneal administration of CNO (3 mg/kg). **(J)** *F*_(4, 95)_ = 81.89, *P* < 0.001; *t* = 8.50, *P* < 0.001, GCaMP6-Naive-saline vs. GCaMP6-SNI-saline; *t* = 1.54, *P* = 0.23, GCaMP6-Naive-CNO vs. GCaMP6-SNI-CNO. **(K**–**O)** Heat maps, averaged *t*races, and summary showing responses of GCaMP6 signal to mechanical stimulation on the ipsilateral hind paw in GCaMP6-Naive (*n* = 6), GCaMP6-SNI (*n* = 6), and eYFP (*n* = 6) mice, before and after intraperitoneal administration of CNO (3 mg/kg). **(O)** *F*_(4, 95)_ = 112.5, *P* < 0.001. *t* = 8.36, *P* < 0.001, GCaMP6-Naive-saline vs. GCaMP6-SNI-saline. *t* = 1.56, *P* = 0.23, GCaMP6-Naive-CNO vs. GCaMP6-SNI-CNO. **(P**–**T)** Heat maps, averaged *t*races, and summary showing changes of GCaMP6 signal during episodes of voluntary movemen*t* in GCaMP6-Naive (*n* = 6), GCaMP6-SNI (*n* = 6), and eYFP (*n* = 6) mice, after intraperitoneal administration of saline or CNO (3 mg/kg). **(T)** *F*_(4, 95)_ = 6.06, *P* < 0.001. *t* = 0.042, *P* = 0.99, GCaMP6-Naive-saline vs. GCaMP6-SNI-saline. *t* = 1.81, *P* = 0.14, GCaMP6-Naive-CNO vs. GCaMP6-SNI-CNO. ** *P* < 0.01; ns not significant. One-way ANOVAs with Bonferroni pos*t*-hoc tests for **(J, O, T)**. Data are available in S1 Data as a part of Supporting information.

To address whether STN neurons control pain responses in VP cholinergic neurons, we transfected GCaMP6s into VP cholinergic neurons by injecting AAV-EF1α-DIO-GCaMP6s into the VP of ChAT-Cre mice and injected AAV-CaMKII-hM4Di-mCherry into the STN (Fig 2A–2D). GCaMP6- and mCherry-labeled neurons in the VP are mostly cholinergic neurons (83%, 111 out of 134 neurons from 4 mice) and STN CaMKII neurons (89%, 812 out of 912 neurons from 4 mice), respectively (Fig 2C and 2E). Some mice were subjected to spared sciatic nerve injury (SNI) to establish neuropathic pain. Fiber photometry recording from VP cholinergic neurons showed that mechanical pressures (Naïve mice: 2 g von Frey filament; SNI mice: 0.4 g von Frey filament) on either hind paw evoked stronger increases in VP GCaMP6 signal in SNI mice than sham mice (Fig 2F, 2G, 2J, 2K, 2L, and 2O), moreover, these increases were dramatically diminished after STN neurons were chemogenetically inhibited in hM4Di mice by intraperitoneal administration of CNO (3 mg/kg) (Fig 2H–2J and 2M–2O–2O). Note that similar alterations in GCaMP6 signal during voluntary movement were observed between naïve and SNI mice, furthermore, these responses were not changed by chemogenetic inhibition of STN neurons (Fig 2P–2T). These data suggest that STN CaMKII neurons control sensory aspect of responses to mechanical stimulation on hind paws in both physiological and neuropathic pain conditions.

As illustrated in Fig 2G, 2I, 2L, and 2N, responses in VP cholinergic neurons to mechanical stimulation appear different in kinetics between sham and SNI mice. We measured decay time of mechanical responses in VP cholinergic neurons, and found that mechanical stimulation of either contralateral and ipsilateral hind paw evoked an increase of GCaMP6 signal in VP cholinergic neurons with a slower decay time and a larger area under the curve (AUC), but these differences were not eliminated by injection of CNO to inhibit STN neurons (S4A–S4D Fig). Together with Fig 2, these data suggest that the STN-VP projection may among many factors that may contribute to the enhancement of mechanical responses in VP cholinergic neurons in neuropathic pain condition.

In the experimental configuration shown in Fig 2A, CNO inhibits STN neurons projecting to the VP and other nuclei, which may complicate data interpretation. To mitigate this caveat, we combined retrograde and Flp-recombinase-dependent strategy to transfect hM4Di into VP-projecting STN neurons, and performed fiber photometry recording from VP cholinergic neurons (S5A and S5B Fig). We observed that inhibition of VP-projecting STN neurons attenuated contralateral and ipsilateral pain stimulation-induced elevation of GCaMP6 signal in VP cholinergic neurons in both naïve and SNI mice (S5C–S5N Fig). Note that paw lifting during locomotion induced only minor increase of GCaMP6 signal in VP cholinergic neurons (S5O–S5R Fig). These data further confirm that the STN-VP projection provides pain signal to VP cholinergic neurons.

To address the synaptic mechanism underlying the enhancement of mechanical responses in VP cholinergic neurons in SNI mice relative to sham mice, we recorded VP cholinergic neurons with the whole-cell patch-clamp technique (S6A and S6B Fig) and observed that the postsynaptic currents evoked by optogenetic stimulation of the STN-VP projection were enhanced in VP cholinergic neurons with increased amplitude and paired pulse ratio (S6C–S6F Fig). These data suggest that the STN-VP projection to VP cholinergic neurons is enhanced via both pre- and post-synaptic mechanisms.

**Fig 3:**
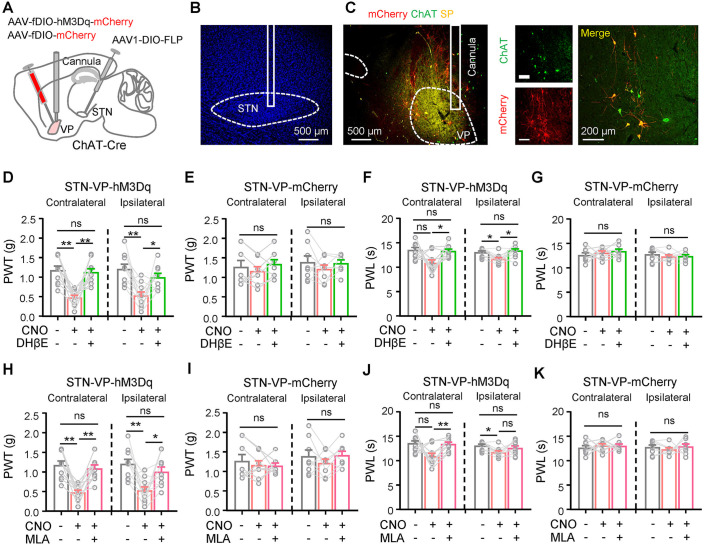
Stimulation of VP cholinergic neurons receiving projection from the STN regulates pain thresholds through activation of α4 and α7 nAChRs in the VP. **(A)** Schematic diagram for labeling of STN-innervated VP ChAT neurons with hM3Dq-mCherry with anterograde viral vector and Cre- and Flp-recombination. **(B)** Needle track for injection of AAV1-hSyn-DIO-Flp into the STN. **(C)** Representative images showing transfection of hM3Dq-mCherry into VP cholinergic neurons. **(D**–**G)** PWT and PWL on hind paws of mice transfected with hM3Dq-mCherry (*n* = 10) or mCherry (*n* = 8) into VP cholinergic neurons before and after injection of CNO and CNO + DHβE into the VP. **(D)** PWT in hM3Dq. Contralateral: *F*_(1.919, 17.27)_ = 15.57, *P* < 0.001; *t* = 5.24, *P* = 0.002, baseline vs. CNO; *t* = 4.93, *P* = 0.002, CNO vs. CNO-DHβE; *t* = 0.34, *P* = 0.74, baseline vs. CNO-DHβE. Ipsila*t*eral: *F*_(1.655, 14.89)_ = 10.95, *P* = 0.0018; *t* = 4.14, *P* = 0.0075, baseline vs. CNO; *t* = 2.82, *P* = 0.040, CNO vs. CNO-DHβE; *t* = 1.95, *P* = 0.08, baseline vs. CNO-DHβE. **(E)** PWT in mCherry. Con*t*ralateral: *F*_(1.144, 8.008)_ = 0.92, *P* = 0.38. Ipsilateral: *F*_(1.392, 9.745)_ = 0.62, *P* = 0.50. **(F)** PWL in hM3Dq. Contralateral: *F*_(1.427, 12.85)_ = 5.91, *P* = 0.022; *t* = 2.46, *P* = 0.07, baseline vs. CNO; *t* = 2.97, *P* = 0.046, CNO vs. CNO-DHβE; *t* = 0.37, *P* = 0.71, baseline vs. CNO-DHβE. Ipsila*t*eral: *F*_(1.441, 12.97)_ = 10.96, *P* = 0.003; *t* = 3.57, *P* = 0.02, baseline vs. CNO; *t* = 3.56, *P* = 0.02, CNO vs. CNO-DHβE; *t* = 1.29, *P* = 0.23, baseline vs. CNO-DHβE. **(G)** PWL in mCherry. Con*t*ralateral: *F*_(1.784, 12.49)_ = 0.66, *P* = 0.52. Ipsilateral: *F*_(1.911, 13.38)_ = 0.71, *P* = 0.50. **(H**–**K)** PWT and PWL on hind paws of mice transfected with hM3Dq-mCherry or mCherry into VP cholinergic neurons before and after injection of CNO and CNO + MLA into the VP. **(H)** PWT in hM3Dq. Contralateral: *F*_(1.692, 15.23)_ = 17.00, *P* < 0.001; *t* = 5.25, *P* = 0.0011, baseline vs. CNO; *t* = 5.99, *P* < 0.001, CNO vs. CNO-MLA; *t* = 0.55, *P* = 0.59, baseline vs. CNO-MLA. Ipsila*t*eral: *F*_(1.955, 17.59)_ = 9.97, *P* = 0.0013; *t* = 4.14, *P* = 0.0075, baseline vs. CNO; *t* = 2.98, *P* = 0.031, CNO vs. CNO-MLA; *t* = 1.41, *P* = 0.19, baseline vs. CNO-MLA. **(I)** PWT in mCherry. Con*t*rala*t*eral: *F*_(1.695, 11.87)_ = 0.46, *P* = 0.61. Ipsila*t*eral: *F*_(1.155, 8.084)_ = 1.48, *P* = 0.26. **(J)** PWL in hM3Dq. Contralateral: *F*_(1.355, 12.20)_ = 6.30, *P* = 0.02; *t* = 2.46, *P* = 0.07, baseline vs. CNO; *t* = 4.20, *P* = 0.007, CNO vs. CNO-MLA; *t* = 0.22, *P* = 0.83, baseline vs. CNO-MLA. Ipsila*t*eral: *F*_(1.561, 14.05)_ = 4.39, *P* = 0.04; *t* = 3.57, *P* = 0.02, baseline vs. CNO; *t* = 1.11, *P* = 0.30, CNO vs. CNO-MLA; *t* = 1.55, *P* = 0.29, baseline vs. CNO-MLA. **(K)** PWL in mCherry. Con*t*rala*t*eral: *F*_(1.856, 12.99)_ = 0.29, *P* = 0.74. Ipsila*t*eral: *F*_(1.356, 9.494)_ = 0.40, *P* = 0.60. * *P* < 0.05; ** *P* < 0.01; ns not significant. One-way repeated measures ANOVAs with Bonferroni tests for **(D**–**K)**. Data are available in S1 Data as a part of Supporting information.

To further confirm that VP cholinergic neurons innervated by the STN modulate pain threshold, we combined transsynaptic anterograde viral vector (AAV1-hSyn-DIO-Flp) with ChAT-Cre mice to transfect AAV-hSyn-fDIO-hM3Dq-mCherry into VP cholinergic neurons innervated by STN neurons and implanted cannula into the VP (Fig 3A–3C). In this set of experiments, hM3Dq was transfected into 73% VP cholinergic neurons with a high specificity (91%) (6 slices from 3 mice), and stimulating these neurons by locally injecting CNO (3 µM) caused mechanical allodynia (Fig 3D, 3E, 3H, and 3I) and thermal hypersensitivity in mice (Fig 3F, 3G, 3J, and 3K). Considering that stimulating VP cholinergic neurons may increase the release of acetylcholine (ACh) in the VP and activate adjacent neurons, we then examined whether blocking nicotinic receptors in the VP attenuate reduction of mechanical and thermal thresholds by VP cholinergic neurons. As expected, co-application of CNO with nicotinic receptor antagonists, dihydro-β-erythroidine (DHβE, 0.3 µM, 200 nl) (Fig 3D–3G) or methyllycaconitine (MLA, 2 µM, 200 nl) (Fig 3H–3K) blocked the effects of CNO on mechanical and thermal thresholds. These data suggest that the stimulation of VP cholinergic neurons may induce mechanical allodynia and thermal hypersensitivity by activating nicotinic receptors in the VP.

In summary, these results indicate that the STN^Glu^-VP^ChAT^ projection is involved in pain modulation in which nicotinic receptors in the VP play important roles. We next tried to elaborate VP cholinergic circuits in pain modulation.

### Mechanical stimulation-induced ACh release from VP cholinergic neurons is enhanced in neuropathic pain mice

**Fig 4:**
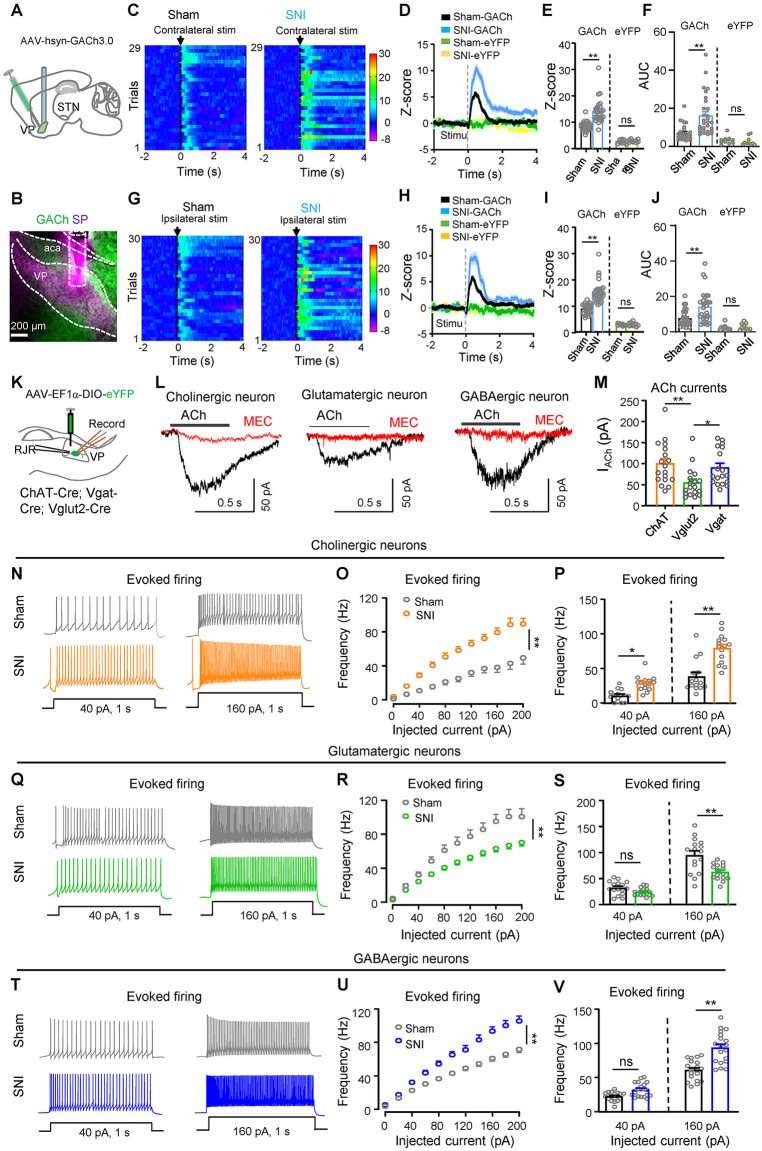
Enhancement of ACh release in the VP results in differential effects on the activity of VP neurons in SNI mice. **(A, B)** Schematic diagram and a representative image showing injection of AAV-hSyn-GACh3.0 and implantation of an optical fiber into the VP. **(C**–**F)** Heat maps, averaged traces, and summary showing responses of GACh signal in the VP to mechanical stimulation on the contralateral hind paw in GACh and eYFP mice subjected to sham or spared sciatic nerve injury (SNI) surgery. **(E)** Peak z-score. *F*_(1, 37)_ = 11.85, *P* = 0.0014; *t* = 7.25, *P* < 0.001, Sham-ACh vs. SNI-ACh; *t* = 0.26, *P* = 0.79, Sham-eYFP vs. SNI-eYFP. **(F)** AUC of z-score. *F*_(3, 74)_ = 15.59, *P* < 0.001; *t* = 4.91, P < 0.001, Sham-GACh vs. SNI-GACh; *t* = 0.17, *P* = 0.86, Sham-eYFP vs. SNI-eYFP. GACh: sham (*n* = 29 *t*rials), SNI (*n* = 29 trials). eYFP: sham (*n* = 10), SNI (*n* = 10). **(G**–**J)** Hea*t* maps, averaged traces, and summary showing responses of GACh signal in the VP to mechanical stimulation on the ipsilateral hind paw in GACh and eYFP mice subjected to sham or SNI surgery. **(I)** Peak z-score. *F*_(1, 38)_ = 17.16, *P* < 0.001; *t* = 8.37, *P* < 0.001, Sham-ACh vs. SNI-ACh; *t* = 0.05, *P* = 0.95, Sham-eYFP vs. SNI-eYFP. *n* = 6 mice in each group. 4-5 trials were performed in each mouse. **(J)** AUC of z-score. *F*_(3, 76)_ = 14.98, *P* < 0.001; *t* = 4.11, *P* < 0.001, Sham-GACh vs. SNI-GACh; *t* = 0.86, *P* = 0.17, Sham-eYFP vs. SNI-eYFP. Sham (*n* = 30 trials), SNI (*n* = 30 *t*rials). eYFP: sham (*n* = 10 trials), SNI (*n* = 10 trials). **(K)** Schema*t*ic diagram for viral vector-assis*t*ed cell-specific fluorescence labeling of VP cholinergic, glutamatergic, and GABAergic neurons in ChAT-Cre, Vglut2-Cre, and Vgat-Cre mice. Patch-clamp recording was performed to measure acetylcholine (ACh)-evoked responses from eYFP-labeled VP neurons. **(L, M)** Representative traces and summary of ACh-evoked responses from eYFP-labeled VP cholinergic (*n* = 18), glutamatergic (*n* = 18), and GABAergic (*n* = 18) neurons before and after bath-perfusion of 10 μM mecamylamine (MEC). *F*_(2, 51)_ = 5.56, *P* = 0.007; *t* = 3.16, *P* = 0.008, ChAT vs. Vglut2; *t* = 2.51, *P* = 0.03, Vglut2 vs. Vgat; *t* = 0.65, *P* = 0.52, ChAT vs. Vgat. Da*t*a for each neuron types were from 3 mice. **(N, O)** Represen*t*ative traces and summary of firing evoked by depolarizing curren*t* injections in VP cholinergic neurons from sham and SNI mice (3 for each group). Currents, *F*_(10, 154)_ = 40.82, *P* < 0.001; Group, *F*_(1, 154)_ = 273.1, *P* < 0.001; Interaction, *F*_(10, 154)_ = 5.75, *P* < 0.001. Data from 15 neurons from each group. **(P)** Summary of firing rate when VP cholinergic neurons were depolarized by injecting 40 and 160 pA currents. *F*_(3, 56)_ = 43.37, *P* < 0.001; 40 pA, *t* = 2.87, *P* = 0.012, Sham vs. SNI; 160 pA, *t* = 6.58, *P* < 0.001, Sham vs. SNI. **(Q, R)** Representative traces and summary of firing evoked by depolarizing current injections in VP glutamatergic neurons from sham and SNI mice (n = 3 in each group). Currents, *F*_(10, 165)_ = 57.12, *P* < 0.001; Group, *F*_(1, 165)_ = 83.66, *P* < 0.001; Interac*t*ion, *F*_(10, 165)_ = 2.41, *P* = 0.001. Da*t*a from 16 neurons in each group. **(S)** Summary of firing rate when VP glutamatergic neurons were depolarized by injecting 40 and 160 pA currents. *F*_(3, 60)_ = 43.55, *P* < 0.001. Forty pA, *t* = 1.15, *P* = 0.44, Sham vs. SNI. 160 pA, *t* = 4.65, *P* < 0.001, Sham vs. SNI. **(T, U)** Representative traces and summary of firing evoked by depolarizing current injections in VP GABAergic neurons from sham and SNI mice (*n* = 3). Currents, *F*_(10, 198)_ = 99.34, *P* < 0.001; Group, *F*_(1, 198)_ = 235.7, *P* < 0.001; Interaction, *F*_(10, 198)_ = 7.43, *P* < 0.001. Data from 18 neurons from each group. **(V)** Summary of firing rate when VP glutamatergic neurons were depolarized by injecting 40 and 160 pA curren*t*s. *F*_(3, 72)_ = 87.73, *P* < 0.001. Forty pA, *t* = 1.99, *P* = 0.09, Sham vs. SNI. 160 pA, *t* = 6.75, *P* < 0.001, Sham vs. SNI. * *P* < 0.05; ** *P* < 0.01; ns not significant. Two-way ANOVA with Bonferroni’s post-hoc analysis for **(E, F, I, J, O, P, R, S, U, V)**. One-way ANOVA in **(M)**. Data are available in S1 Data as a part of Supporting information.

Our previous study reports that activity of VP cholinergic and non-cholinergic neurons is enhanced in chronic pain [19]. We postulate that VP cholinergic neurons may excite non-cholinergic neurons by releasing ACh. To measure ACh levels in the VP, we injected AAV-hSyn-GACh3.0 or AAV-hSyn-eYFP into the right VP and implanted an optical fiber into the injection site of the viral vector (Fig 4A and 4B). Fiber photometry recording of GACh signal in the VP showed that mechanical stimulation (2 g von Frey filament) of either hind paw increased GACh signal, but not eYFP signal in the VP (Fig 4C–4J). Additionally, GACh signal did not change when the mice randomly lifted either hind paw (S7A–S7C Fig). These results suggest that the increase of GACh signal in response to mechanical stimulation is most likely caused by the sensory aspect of the stimulation, but not by the action of paw withdrawal. Furthermore, we observed that mechanical stimulation (0.4 g von Frey filament)-evoked increase of GACh signal but not eYFP signal in the VP was significantly enhanced in SNI mice relative to sham mice (Fig 4C–4J). These data suggest that pain stimulation may cause more ACh release in the VP, consistent with the hyperexcitability of VP cholinergic neurons in SNI mice [19].

Considering that VP cholinergic neurons may not be the only source of ACh in the VP, to examine whether VP cholinergic neurons play a significant role in mechanical stimulation-induced ACh release in the VP, we injected AAV-hSyn-GACh3.0 and AAV-EF1α-DIO-hM4Di-mCherry, and implanted an optical fiber into the right VP of ChAT-Cre mice (S7D Fig). In this set of experiment, hM4Di was transfected into VP cholinergic neurons (S7E–S7G Fig). In naïve mice, mechanical stimulation increased GACh signal in the VP with high specificity (>90%) and high efficiency (>75%) (S7E–S7G Fig). The increase in GACh following mechanical stimulation was significantly attenuated by inhibiting VP cholinergic neurons with CNO (S7H, S7I, S7L–S7N, and S7Q Fig). Similar results were observed in SNI mice (S7J–S7L and S7O–S7Q Fig). These data support that VP cholinergic neurons significantly contribute to pain-related ACh release in the VP.

To address whether VP neurons respond to ACh, we injected AAV-EF1α-DIO-eYFP into the VP of ChAT-Cre, Vglut2-Cre, and Vgat-Cre mice to label cholinergic, glutamatergic, and GABAergic neurons, and conducted patch-clamp recording from eYFP-labeled neurons (Figs 4K, S8A, and S8B). To confirm the specificity of eYFP-labeled neurons with this strategy, we collected eYFP-positive VP neurons (20–30 neurons per vial) from 15 μm-thick frozen brain sections mounted onto a RNase-free glass slide using a laser capture microscope system (S8C and S8D Fig). Then, we conducted quantitative real-time PCR (qRT-PCR) analysis of the mRNA of genes encoding neuronal marker proteins (*ChAT* for cholinergic neurons, *Vglut2* for glutamatergic neurons, and *Vgat* for GABAergic neurons) (S8E and S8F Fig). The qRT-PCR data indicate that the viral vector can specifically label Cre-positive neurons (S8E and S8F Fig; S1 Table). This is consistent with previous studies showing that cholinergic, glutamatergic, and GABAergic neurons in the VP are largely separated [24–27].

Using the patch-clamp technique (Fig 4K), we recorded ACh-evoked inward currents in most neurons (>80%) of each type, and the currents were almost eliminated by 10 μM mecamylamine (MEC), a broad-spectrum antagonist of nAChRs (Fig 4L). But the amplitude of ACh-induced currents in these three types of neurons was not significantly altered in the presence of 0.5 μM tetrodotoxin (TTX) (which limits presynaptic neurotransmitter release) or mixture of 20 μM CNQX and 10 μM GABAzine (blocker of glutamate receptor and GABA_A_ receptor) (S8G–S8L Fig). These data suggest that ACh puff-induced inward currents are primarily mediated by nAChRs on VP neurons. Interestingly, the amplitude of ACh currents in glutamatergic neurons were smaller than those in other types of neurons (Fig 4M).

After confirmed the expression of nAChRs on VP neurons, we wondered whether the excitability of VP neurons is modified in SNI mice. We observed that in SNI mice, VP cholinergic neurons exhibited stronger responses to depolarizing stimulation than those in sham mice (Fig 4N–4P), similarly to our previous study [19]; the responses of glutamatergic and GABAergic neurons to injection of depolarizing currents were respectively attenuated (Fig 4Q–4S) and enhanced (Fig 4T–4V), respectively. The alterations of excitability in non-cholinergic neurons in SNI mice are different from our previous study [19], in which we did not separate them into glutamatergic and GABAergic neurons and observed hyperactivity in non-cholinergic neurons. Because GABAergic neurons are overnumbered glutamatergic neurons in the VP [26,27], in our previous study, a lot more GABAergic neurons may be sampled and the overall changes in the activity of non-cholinergic neurons may be determined by changes of GABAergic neurons.

To address whether alterations in activity of VP neurons in SNI mice are related to nAChRs or major presynaptic neurotransmitters, we examined spontaneous and depolarizing currents-evoked firing in these neurons in the absence and presence of MEC or CNQX + GABAzine (S9A–S9O Fig).

We observed that blocking GABA_A_ and glutamate receptors with GABAzine and CNQX reduced spontaneous firing in glutamatergic neurons (S9K and S9L Fig), but not in cholinergic (S9A and S9B Fig) and GABA neurons (S9F and S9G Fig), in sham mice, whereas, reduced spontaneous firing in GABA neurons (S9F and S9G Fig), but not in cholinergic (S9A and S9B Fig) and glutamatergic neurons (S9K and S9L Fig), in SNI mice; it did not change depolarizing currents-evoked firing rate in cholinergic neurons (S9C–S9E Fig), GABAergic neurons (S9H–S9J Fig), and glutamatergic neurons (S9M–S9O Fig). These data suggest that presynaptic inputs do affect activity in some neuronal populations in physiological and neuropathic pain conditions. However, blocking GABAergic and glutamatergic inputs eliminated differences in evoked firing in GABAergic (S9H–S9J Fig) and glutamatergic neurons (S9M–S9O Fig), but not in cholinergic neurons (S9C–S9E Fig), between sham and SNI mice.

We also observed very complex results in spontaneous and evoked firing in VP neurons following blockade of nicotinic receptors with MEC. In sham mice, MEC significantly reduced spontaneous firing rate in cholinergic neurons (S9A and S9B Fig) and glutamatergic neurons (S9K and S9L Fig), but not in GABAergic (S9F and S9G Fig), and reduced evoked firing rate in GABAergic (S9H–S9J Fig) and glutamatergic neurons (S9M–S9O Fig), but not in cholinergic neurons (S9C–S9E Fig). In SNI mice, MEC reduced spontaneous firing rate in cholinergic neurons (S9A and S9B Fig), but not in GABA (S9F and S9G Fig) and glutamatergic neurons (S9K and S9L Fig), surprisingly did not affect evoked firing rate in VP neurons (S9C–S9E, S9H–S9J and S9M–S9O Fig). MEC eliminated the differences in spontaneous firing rate and evoked firing rate in VP glutamatergic neurons (S9M–S9O Fig), but not in cholinergic (S9C–S9E Fig) and GABAergic (S9H–S9J Fig) neurons, between sham and SNI mice.

These data hint that tonic activation of nicotinic receptors differentially regulates excitability of three types of VP neurons in physiological and neuropathic pain conditions. In fact, nAChRs in VP neurons and the levels of ACh, GABA, and glutamate are among numerous neuropathological changes that occur in neuropathic pain. Additionally, nAChR subunits may be subjected to modifications in SNI. These may lead to the outcome that blocking either nicotinic receptors or GABA_A_ and glutamate receptors did not consistently eliminate alterations in neuronal excitability in the VP in SNI mice.

### α4 and α7 subunits in VP neurons are differentially modified in VP neurons

**Fig 5:**
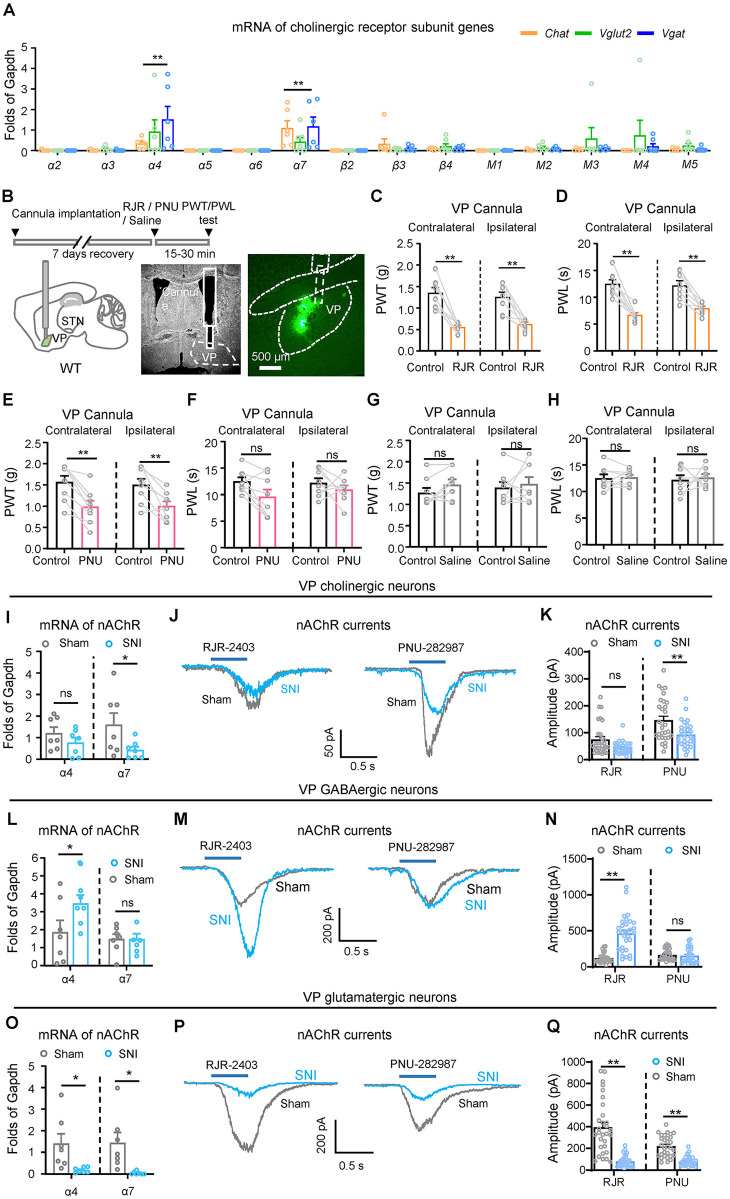
nAChRs in VP neurons are differentially modified in SNI mice. **(A)** qRT-PCR was used to quantify the levels of nicotinic and muscarinic acetylcholine receptor (nAChR and mAChR) subunit genes which were normalized to that of *Gapdh* in eYFP-labeled cholinergic (*n* = 6–7), glutamatergic (*n* = 6–7) and GABAergic (*n* = 6–7) neurons in the VP. *α*4 vs. *α*2, *α*3, *α*5, *α*6, *β*2, *β*3, *β*4, M, M2, M3, M4, M5; P < 0.01. *α*7 vs. *α*2, *α*3, *α*5, *α*6, *β*2, *β*3, *β*4, M1, M2, M3, M4, M5; *P* < 0.01. *α*4 vs. *α*7; *P* = 0.99. mean ± SEM; α4 (ChAT: 0.7 ± 0.20, Vglut2: 1.85 ± 1.15, Vgat: 3.04 ± 1.26); α7 (ChAT: 2.2 ± 0.72, Vglut2: 0.87 ± 0.42, Vgat: 2.36 ± 0.89). **(B)** Schematic diagram for intracranial injection of nAChR agonists into the VP and a representative image for position of cannula and spreading of fluorescence dye injected into the VP. **(C, D)** Mechanical PWT and thermal PWL were measured before and after injection of RJR-2403 (RJR) into the VP (*n* = 8). **(C)** PWT. *F*_(1.460, 10.22)_ = 19.77, *P* < 0.001. Contralateral: *t* = 6.35, *P* = 0.0008, control vs. RJR. Ipsilateral: *t* = 5.32, *P* = 0.001, control vs. RJR. **(D)** PWL. *F*_(2.542, 17.79)_ = 24.46, *P* < 0.001. Contrala*t*eral: *t* = 6.45, *P* < 0.001, control vs. RJR. Ipsilateral: *t* = 4.92, *P* = 0.002, con*t*rol vs. RJR. **(E, F)** Mechanical PWT and thermal PWL were measured before and after injection of PNU-282987 (PNU) in*t*o the VP (*n* = 8). **(E)** PWT. *F*_(1.877, 13.14)_ = 6.73, *P* = 0.01. Contralateral PWT: *t* = 5.48, *P* = 0.002, Control vs. PNU. Ipsilateral PWT: *t* = 3.83, *P* = 0.006, Control vs. PNU. **(F)** PWL. *F*_(2.359, 16.51)_ = 2.34, *P* = 0.12. **(G, H)** Mechanical PWT and *t*hermal PWL were measured before and after injection of saline in*t*o the VP (*n* = 8). **(G)** PWT. *F*_(1.724, 12.07)_ = 0.45, *P* = 0.62. **(H)** PWL. *F*_(1.676, 11.73)_ = 0.11, *P* = 0.87. **(I)** Quantification of *α4* and *α7* mRNA levels in VP cholinergic neurons in sham (*n* = 7) and SNI (*n* = 7) mice. *α4*: *t* = 0.94, *P* = 0.36, Sham vs. SNI; *α7*: *t* = 2.47, *P* = 0.04, Sham vs. SNI. **(J, K)** Representative traces and summary of RJR-2403 and PNU282987-induced inward curren*t*s in VP cholinergic neurons in sham and SNI mice. RJR: *t* = 2.12, *P* = 0.06, Sham vs. SNI. PNU: *t* = 3.79, *P* < 0.001, Sham vs. SNI. **(L)** Quantification of *α4* and *α7* mRNA levels in VP GABAergic neurons in sham (*n* = 7) and SNI (*n* = 7) mice. *α4*: *t* = 2.47, *P* = 0.04, Sham vs. SNI; *α7*: *t* = 0.02, *P* = 0.98, Sham vs. SNI. **(M, N)** Represen*t*ative traces and summary of RJR-2403 and PNU282987-induced inward currents in VP GABAergic neurons in SNI and sham mice. RJR: *t* = 9.25, *P* < 0.001, Sham vs. SNI. PNU: *t* = 0.26, *P* = 0.95, Sham vs. SNI. **(O)** Quantification of *α4* and *α7* mRNA levels in VP glutamatergic neurons in sham (*n* = 7) and SNI (*n* = 7) mice. *α4*: *t* = 2.55, *P* = 0.02, Sham vs. SNI; *α7*: *t* = 2.91, *P* = 0.02, Sham vs. SNI. **(P, Q)** Represen*t*ative traces and summary of RJR-2403 and PNU282987-induced inward currents in VP glutama*t*ergic neurons in SNI and sham mice. RJR: *t* = 8.88, < 0.001, Sham vs. SNI. PNU: *t* = 3.84, *P* < 0.001, Sham vs. SNI. * *P* < 0.05; ** *P* < 0.01; ns not significant. Two-way ANOVAs with Bonferroni tests for **(A)**. Two-*t*ailed paired *t*-*t*ests for **(C**–**H)**. Two-tailed *t*-tests for **(I, K, L, N, O, Q)**. Data are available in S1 Data as a part of Supporting informa*t*ion.

To address whether in SNI mice, the major acetylcholine receptor subtypes in three types of VP neurons are differentially modified, we collected eYFP-labeled VP neurons and analyzed the expression of genes of nicotinic and muscarinic acetylcholine receptors (nAChRs and mAChRs) (S10A and S10B Fig). We observed that the major nAChRs in three neuron types contained mRNA of α4 and α7 subunit genes (*Chrna4* and *Chrna7*), while muscarinic receptors in glutamatergic neurons contained mRNA of M3 and M4 subunit genes (*Chrm3* and *Chrm4*) (Fig 5A and S1 Table). To address whether α4 and α7 nAChRs are tonically activated and involved in pain modulation, we injected DHβE (antagonist of α4 nAChRs) (0.3 μM, 200 nl) and MLA (antagonist of α7 nAChRs) (2 μM, 200 nl) into the VP and measured mechanical and thermal thresholds on hind paws (Fig 5B). We observed no difference in pain thresholds before and after drug administration into the VP in naïve mice (S10C–S10F Fig). These data suggest that in physiological condition, blocking α4 or α7 nAChRs does not change mechanical and thermal thresholds.

To address whether activation of nAChRs in the VP modulate mechanical and thermal thresholds, we then injected nAChR subtype-selective agonists, RJR-2403 (agonist of α4 nAChRs) (50 μM, 200 nL) and PNU-282987 (agonist of α7 nAChRs) (30 μM, 200 nL), into the VP (Fig 5B). We found that activation of α4 nAChRs in the VP significantly reduced mechanical PWT and thermal PWL on both hind paws (Fig 5C and 5D); activation of α7 nAChRs in the VP significantly reduced mechanical PWT, but not thermal PWL, on both hind paws (Fig 5E and 5F); injection of saline into the VP changed neither PWT nor PWL on hind paws (Fig 5G and 5H). Therefore, activation of major nAChR subtypes in the VP is sufficient to cause mechanical and thermal hypersensitivity. These data are consistent with the notion that enhancement of VP cholinergic circuit may be a major pathophysiology of hyperalgesia in neuropathic pain.

We then measured mRNA levels of acetylcholine receptor subunits in VP cholinergic, GABAergic and glutamatergic neurons in sham and SNI mice. We observed that in SNI mice, mRNA levels of *Chrna4* subunit were elevated in GABAergic neurons, reduced in glutamatergic neurons, but were not altered in cholinergic neurons; mRNA levels of *Chrna*7 gene were reduced in cholinergic and glutamatergic neurons, but were not altered in GABAergic neurons (Fig 5I, 5L, and 5O). Additionally, we also observed that in cholinergic neurons, SNI upregulated β3, M1, and M5 subunit genes, while downregulated M4 subunit gene (S10G Fig); in glutamatergic neurons, SNI upregulated M4 subunit gene (S10H Fig); in GABAergic neurons, SNI upregulated α5, M1, and β3 subunit genes (S10I Fig). Considering α4 and α7 subunit-containing nAChRs are major nAChRs in the central nervous system and have selective agonists and antagonists, we compared functions of α4 and α7 nAChRs in VP neurons between sham and SNI mice with the patch-clamp technique. We observed that the amplitude of currents evoked by puffing RJR-2403 (50 μM) or PNU-282987 (30 μM) onto cholinergic, GABAergic, and glutamatergic neurons in the VP were modified in a pattern consistent with the levels of mRNAs of *Chrn*α*4* and *Chrn*α*7* genes (Fig 5I–5Q). Therefore, in SNI mice, α4 nAChRs may be upregulated in VP GABAergic neurons, in contrast, α4 and α7 nAChRs were downregulated in VP glutamatergic neurons.

To address the contribution of changes in nAChRs to hyperalgesia in SNI mice, we constructed viral vectors to fulfill cell-specific regulation of α4 and α7 nAChRs and examined whether reversal of changes in α4 and α7 nAChRs alleviates hyperalgesia in SNI mice.

### Downregulation of α4 nAChR subunit in VP GABAergic neurons distinctly reduces pain thresholds but attenuates hyperalgesia in neuropathic pain

**Fig 6:**
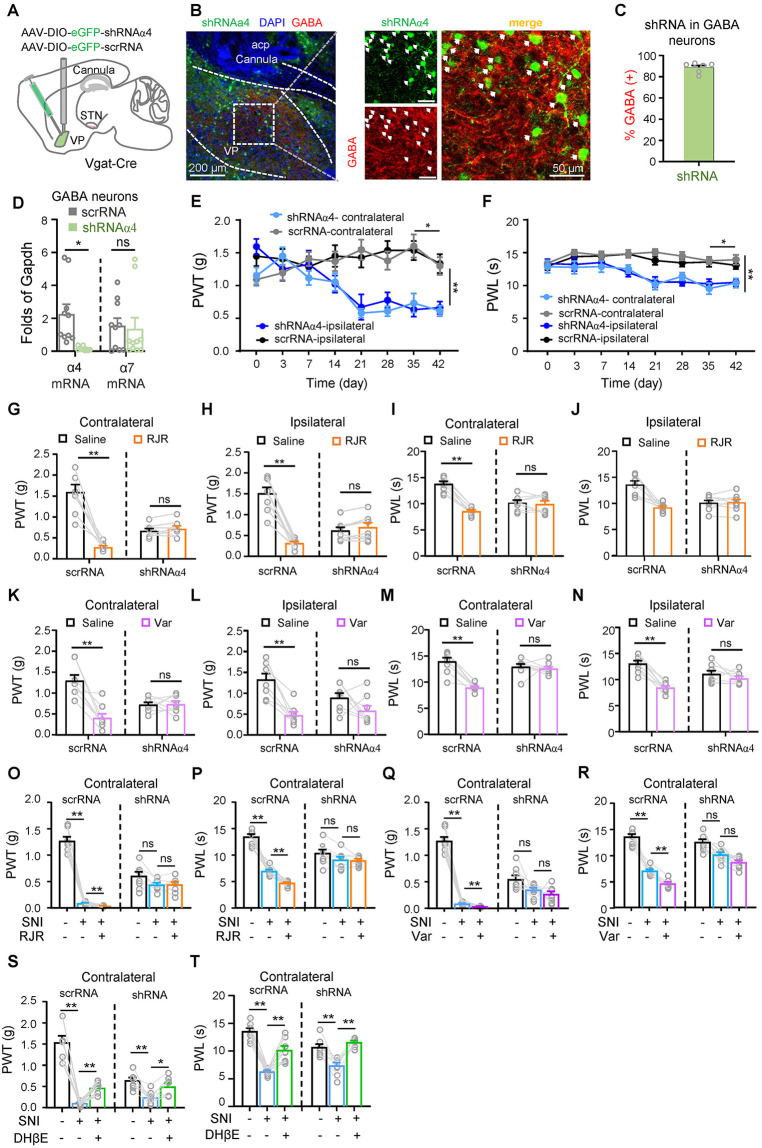
Down-regulation of α4 nAChR subunit in VP GABAergic neurons mitigates hyperalgesia in SNI mice. **(A)** Schematic diagram for injection of AAV-CMV-DIO-EGFP-shRNA(Chrna4) (shRNA) or AAV-CMV-DIO-eGFP-shRNA(scramble) (scrRNA) and implantation of a cannula into the right VP of Vgat-Cre mice. **(B, C)** Representative images and summary showing eGFP expression (green) in GABAergic neurons (red) 3 weeks after virus injection. GABA and shRNA co-expression neurons accounted for 88.64% of shRNA neurons. Summary data were from 6 slices of 3 mice. **(D)** The levels of *α4* and *α7* subunit mRNA in VP GABAergic neurons in shRNA and scrRNA mice were quantified with qRT-PCR assay. Quantification of mRNA levels of nAChR α4 and α7 subunits on VP GABAergic neurons from shRNA (*n* = 8) and scrRNA (*n* = 8) mice. Interaction: *F*_(1, 28)_ = 8.59, *P* = 0.006; Group: *F*_(1, 28)_ = 11.44, P = 0.0021; Receptor: *F*_(1, 28)_ = 5.55, *P* = 0.026. α4: *t* = 3.74, *P* = 0.001, shRNA-α4 vs. scrRNA. α7: *t* = 0.41, *P* = 0.90, shRNA-α4 vs. scrRNA. **(E, F)** Time courses of PWT and PWL on either hind paw in mice subjected to injection of AAV-CMV-DIO-shRNA-α4-eGFP (shRNA-α4) or AAV-CMV-DIO-scramble RNA-eGFP (scrRNA). **(E)** PWT. Interaction, *F*_(21, 196)_ = 3.74, *P* < 0.001; Group, *F*_(3, 28)_ = 21.12, *P* < 0.001; Time, *F*_(5.678, 159.0)_ = 3.92, *P* = 0.001. **(F)** PWL. Interaction, *F*_(21, 196)_ = 1.84, *P* = 0.018; Group, *F*_(3, 28)_ = 18.17, *P* < 0.001; Time, *F*_(3, 28)_ = 18.17, *P* < 0.001. **(G**–**N)** PWT and PWL on either hind paw before and after microinjection of an α4 nAChR agonist: RJR-2403 (RJR) or varenicline (Var), into the VP of shRNA (*n* = 8) and scrRNA (*n* = 8) mice. **(G)** Contralateral PWT. *F*_(1, 14)_ = 47.84, *P* < 0.001. scrRNA: *t* = 9.42, *P* < 0.001, saline vs. RJR. shRNAa4: *t* = 0.36, *P* = 0.73, saline vs. RJR. **(H)** Ipsilateral PWT. *F*_(1, 14)_ = 86.04, *P* < 0.001. scrRNA: *t* = 12.27, *P* < 0.001, saline vs. RJR. shRNAa4: *t* = 0.85, *P* = 0.41, saline vs. RJR. **(I)** Con*t*ralateral PWL. *F*_(1, 14)_ = 53.60, *P* < 0.001. scrRNA: *t* = 10.93, *P* < 0.001, saline vs. RJR. shRNAa4: *t* = 0.57, *P* = 0.57, saline vs. RJR. **(J)** Ipsilateral PWL. *F*_(1, 14)_ = 19.39, *P* < 0.001. scrRNA: *t* = 6.91, *P* < 0.001, saline vs. RJR. shRNAa4: *t* = 0.13, *P* = 0.89, saline vs. RJR. **(K)** Con*t*ralateral PWT. *F*_(1, 14)_ = 19.42, *P* < 0.001. scrRNA: *t* = 6.13, *P* < 0.001, saline vs. Var. shRNAa4: *t* = 0.098, *P* = 0.92, saline vs. Var. **(L)** Ipsila*t*eral PWT. *F*_(1, 14)_ = 5.12, *P* = 0.04. scrRNA: *t* = 5.01, *P* < 0.001, saline vs. Var. shRNAa4: *t* = 1.81, *P* = 0.09, saline vs. Var. **(M)** Contralateral PWL. *F*_(1, 14)_ = 19.68, *P* = 0.04. scrRNA: *t* = 6.75, *P* < 0.001, saline vs. Var. shRNAa4: *t* = 0.48, *P* = 0.63, saline vs. Var. **(N)** Ipsila*t*eral PWL. F_(1, 14)_ = 13.66, *P* = 0.0024. scrRNA: *t* = 6.46, *P* < 0.001, saline vs. Var. shRNAa4: *t* = 1.24, *P* = 0.23, saline vs. Var. **(O, P)** PWT and PWL in scrRNA (*n* = 8) and shRNA (*n* = 8) mice before and after SNI surgery and injec*t*ion of RJR2403 into *t*he VP. **(O)** Contrala*t*eral PWT. scrRNA: *F*_(1.043, 7.302)_ = 236.9, *P* < 0.001; *t* = 14.18, *P* < 0.001, con*t*rol vs. SNI; *t* = 3.75, *P* = 0.007, SNI vs. SNI + RJR-2403. shRNA: *F*_(1.686, 11.80)_ = 3.90, *P* = 0.06; *t* = 2.3, *P* = 0.11, con*t*rol vs. SNI; *t* = 0.09, *P* = 0.93, SNI vs. SNI + RJR-2403. **(P)** Con*t*ralateral PWL. scrRNA: *F*_(1.313, 9.193)_ = 141.3, *P* < 0.001; *t* = 9.59, *P* < 0.001, Control vs. SNI; *t* = 7.33, *P* = 0.0003, SNI vs. SNI + RJR-2403. shRNA: *F*_(1.856, 12.99)_ = 4.32, *P* = 0.039; *t* = 2.47, *P* = 0.084, Control vs. SNI; *t* = 0.26, *P* = 0.96, SNI vs. SNI + RJR-2403. **(Q, R)** PWT and PWL in scrRNA (*n* = 8) and shRNA (*n* = 8) mice before and after SNI surgery and injection of Var into the VP. **(Q)** Con*t*ralateral PWT. scrRNA: F_(1.025, 7.176)_ = 241.0, *P* < 0.001; *t* = 14.8, *P* < 0.001, Con*t*rol vs. SNI; *t* = 4.67, *P* = 0.002, SNI vs. SNI + Var. shRNA: F_(1.833, 12.83)_ = 4.49, *P* = 0.04; *t* = 2.08, *P* = 0.14, Con*t*rol vs. SNI; *t* = 0.95, *P* = 0.37, SNI vs. SNI + Var. **(R)** Contralateral PWL. scrRNA: F_(1.152, 8.065)_ = 143.8, *P* < 0.001; *t* = 9.59, *P* < 0.001, Control vs. SNI; *t* = 11.52, *P* < 0.001, SNI vs. SNI + Var. shRNA: F_(1.598, 11.19)_ = 12.77, *P* = 0.002; *t* = 2.72, *P* = 0.06, Con*t*rol vs. SNI; *t* = 2.74, *P* = 0.06, SNI vs. SNI + Var. **(S, T)** PWT and PWL in scrRNA (*n* = 8) and shRNA (*n* = 8) mice before and af*t*er SNI surgery and injection of DHβE into the VP. **(S)** Contralateral PWT. scrRNA: *F*_(1.147, 8.026)_ = 59.32, *P* < 0.001; *t* = 9.46, *P* < 0.001, Control vs. SNI; *t* = 6.51, *P* < 0.001, SNI vs. SNI + DHβE. shRNA: *F*_(1.647, 11.53)_ = 9.81, *P* = 0.004; *t* = 4.55, *P* = 0.005, Control vs. SNI; *t* = 3.45, *P* = 0.021, SNI vs. SNI + DHβE. **(T)** Con*t*ralateral PWL. scrRNA: *F*_(1.469, 10.28)_ = 45.83, *P* < 0.001; *t* = 14.23, *P* < 0.001, control vs. SNI; *t* = 4.97, *P* = 0.003, SNI vs. SNI + DHβE. shRNA: *F*_(1.832, 12.82)_ = 20.92, *P* < 0.001; *t* = 4.66, *P* = 0.005, con*t*rol vs. SNI; *t* = 5.57, *P* = 0.002, SNI vs. SNI + DHβE. * *P* < 0.05; ** *P* < 0.01; ns not significan*t*. Two-way ANOVAs with Bonferroni *t*ests for **(D)**. Two-way repea*t*ed measures ANOVAs for **(E, F)**. Two-way repeated measures ANOVAs for **(G**–**N)**. One-way repeated measurements ANOVAs with Holm-Sidak’s multiple comparisons for **(O**–**T)**. Da*t*a are available in S1 Data as a part of Suppor*t*ing information.

As in SNI mice, α4 nAChR subunit in VP GABAergic neurons was upregulated, we wondered whether downregulation of α4 nAChR subunit in VP GABAergic neurons mitigates hyperalgesia in SNI mice. To fulfill this goal, we constructed Cre-dependent AAV viral vectors carrying shRNA of α4 nAChR subunit (AAV-hSyn-DIO-Chrna4 shRNA-eGFP) (shRNAa4) or scrambled RNA (AAV-hSyn-DIO-Chrna scrRNA-eGFP) (scrRNA) [28] and injected individual vectors into the VP of Vgat-Cre mice (Fig 6A).

We then applied multidisciplinary techniques to confirm the specificity and effectiveness of the viral vectors in downregulating α4 nAChR subunit in VP GABAergic neurons one month after injection of the viral vector. Our morphological data confirmed that the great majority of virally transfected eGFP-positive neurons in the VP were GABAergic neurons defined by the presence of GABA (Fig 6B and 6C). The qRT-PCR analysis of laser-captured eGFP-positive neurons in the VP showed that shRNA-eGFP-positive neurons exhibited dramatically lower mRNA level of *Chrna4* gene with unchanged mRNA level of *Chrna7* gene, relative to scrRNA-eGFP neurons (Fig 6D). α4 nAChR agonists (50 μM RJR-2403 and 100 μM varenicline) evoked significantly smaller inward currents in shRNAa4-eGFP-positive neurons than scrRNA-eGFP-positive neurons (S11A–S11D Fig). These data indicate that these viral vectors possess specificity and function suitable for down-regulating α4 nAChR subunit in Cre-positive neurons.

We next examined mechanical PWT and thermal PWL in mice subjected to injection of viral vector into the VP of Vgat-Cre mice and observed that up to 3 weeks and beyond after viral vector injection, shRNAa4 mice exhibited a significantly lower mechanical PWT and thermal PWL on either hind paw (Fig 6E and 6F). Relative to scrRNA mice, shRNAa4 mice exhibited similar performance in the OFT, including locomotion and time in the center zone of the open field arena (S11E Fig), and showed mitigated anxiety-like behavior in the elevated plus maze (EPM) (S11F Fig) and unchanged depression-like behavior in the tail suspension test (TST) and forced swim test (FST) (S11G and S11H Fig). These data suggest that tonic activation of α4 nAChR on VP GABAergic neurons may be among analgesic components required for the maintenance of normal mechanical and thermal thresholds, but did not affect locomotion and depression-like behaviors.

We next examined whether downregulation of α4 nAChRs on VP GABAergic neurons affects modulation of mechanical and thermal thresholds by activating α4 nAChRs in the VP. We observed that injection of α4 nAChR agonists, RJR-2403 (500 μM 200 nl) (Fig 6G–6J) and varenicline (1 μg in 200 nl) (Fig 6K–6N), into the VP induced mechanical allodynia and thermal hypersensitivity in scrRNAa4 mice; in a stark contrast, injection of RJR-2403 (Fig 6G–6J) and varenicline (Fig 6K–6N) into the VP did not reduce mechanical PWT and thermal PWL in shRNAa4 mice. These pharmacological data support a notion that α4 nAChRs on GABAergic neurons are important components mediating hyperalgesia following increase of ACh release in the VP. Interestingly, SNI dramatically reduced mechanical PWT and thermal PWL in scrRNA mice, but not in shRNAa4 mice (Fig 6O–6R). Furthermore, in SNI mice, injection of RJR-2403 and varenicline exacerbated hyperalgesia in scrRNA mice, but not in shRNAa4 mice (Fig 6O–6R). We confirmed that after SNI, the mRNA level of *Chrna4* in VP GABAergic neurons in shRNA mice was still significantly lower than that in scrRNA mice (S11I Fig). Note that mechanical PWT and thermal PWL in shRNAa4 mice remained at the similar levels before and after SNI surgery and after injection of α4 nAChR agonist into the VP, and were significantly higher than those in scrRNA mice subjected to SNI surgery and received injection of α4 nAChR agonist into the VP (Fig 6O–6R). Therefore, these data suggest that downregulation of α4 nAChRs on VP GABAergic neurons mitigates hyperalgesia in SNI mice.

As in shRNAa4 mice, α4 nAChRs on VP GABAergic neurons were significantly downregulated, injected α4 nAChR antagonists into the VP may act on α4 nAChRs on neurons and terminals other than VP GABAergic neurons. We observed that injection of DHβE into the VP exhibited similar elevation in mechanical PWT and thermal PWL in scrRNA and shRNAa4 mice subjected to SNI (Fig 6S and 6T). These data suggest that α4 nAChRs on circuit components in the VP other than GABAergic neurons also mediate the analgesic effect of DHβE in the VP in SNI mice.

Considering that VP GABAergic neurons express α7 nAChRs, we next examined whether downregulating these receptors has similar effects as downregulating α4 nAChRs on mechanical and thermal thresholds and development of neuropathic pain (S12A–S12D and S13A–S13D Figs). We observed that downregulation of α7 subunit in shRNAa7 mice did not change either mechanical or thermal thresholds (S12E and S12F Fig), but attenuated reduction of mechanical and thermal thresholds on the ipsilateral side and thermal threshold on the contralateral side, following injection of PNU, an agonist of α7 nAChRs, into the VP (S12G–S12J Fig); it did not affect alterations of mechanical and thermal thresholds in sham mice following MLA injection into the VP (S12K and S12L Fig); it attenuated elevation of mechanical and thermal thresholds in SNI mice (mRNA level of α7 nAChRs remained lower in shRNAa7 mice than that in scrRNA mice) (S13E Fig) following MLA injection into the VP (S12M and S12N Fig). Similar to shRNAa4 mice, shRNAa7 mice did not exhibit alterations in performance in the OFT (distance and time in center zone), TST, FST (S12O, S12Q, and S12R Fig), but mitigation of anxiety in the EPM (S12P Fig). Therefore, α7 nAChRs on VP GABA neurons are involved in modulation of mechanical and thermal thresholds. Although inhibition of these receptors has minor effects on mechanical and thermal thresholds in naïve mice, activation or inhibition of these receptors modulate mechanical and thermal thresholds in physiological or neuropathic pain condition, respectively.

### Downregulation of α4 nAChR subunit in VP glutamatergic neurons may not contribute to hyperalgesia in neuropathic pain

**Fig 7:**
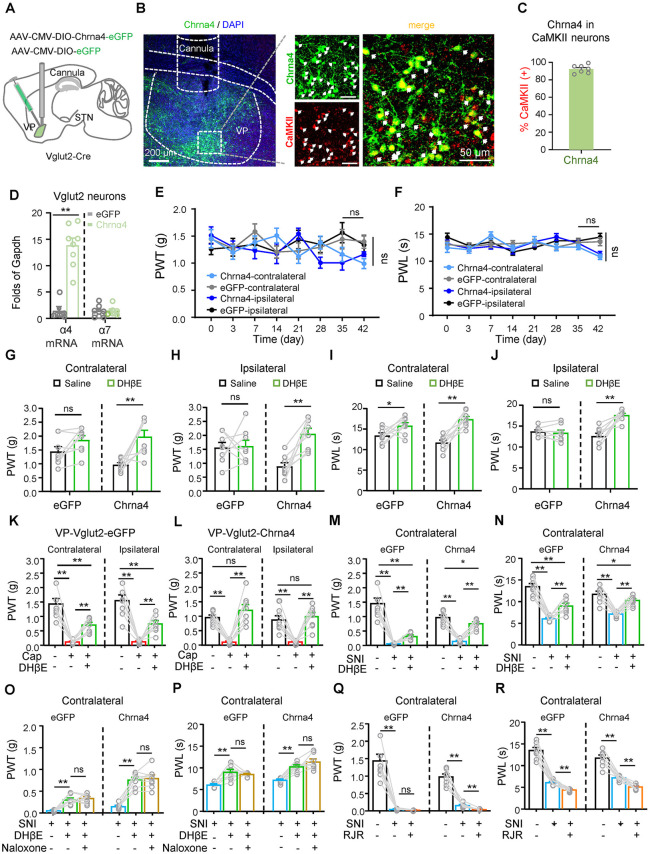
Upregulation of α4 nAChR subunit in VP glutamatergic neurons enhances analgesic effect of DHβE injection into the VP. **(A)** Schematic diagram for injection of AAV-CMV-DIO-EGFP-Chrna4 (Chrna4) or AAV-CMV-DIO-eGFP (eGFP) and implantation of a cannula into the right VP of Vglut2-Cre mice. **(B, C)** Representative images and summary showing eGFP expression (green) in glutamatergic neurons (red) 3 weeks after virus injection. Chrna4-CaMKII co-expression neurons accounted for 92.18% of Chrna4 neurons. Summary data were from 7 slices of 3 mice. **(D)** The mRNA levels of *α4* and *α7* subunit genes in VP glutamatergic neurons in Chrna4 (*n* = 8) and eGFP (*n* = 8) mice were quantified with qRT-PCR assay (S1 Table). Interaction *F*_(1, 28)_ = 61.88, *P* < 0.001; Receptor, *F*_(1, 28)_ = 61.77, *P* < 0.001; Group, *F*_(1, 28)_ = 62.65, *P* < 0.001. *α4*: *t* = 11.16, *P* < 0.001; *α7*: *t* = 0.03, *P* = 0.97. **(E, F)** Time courses of PWT and PWL on either hind paw in mice subjec*t*ed to injec*t*ion of AAV-CMV-DIO-Chrna4-eGFP (Chrna4) or AAV-CMV-DIO-eGFP (eGFP). **(E)** PWT. Interaction, *F*_(21, 196)_ = 1.52, *P* = 0.08; Group, *F*_(3, 28)_ = 1.38, *P* = 0.27; Time, *F*_(5.802, 162.5)_ = 0.99, *P* = 0.44. **(F)** PWL. Interaction, *F*_(21, 196)_ = 2.02, *P* = 0.007; Group, *F*_(3, 28)_ = 2.37, *P* = 0.09; Time, *F*_(5.757, 161.2)_ = 2.18, *P* = 0.05. **(G**–**J)** PWT and PWL on either hind paw before and after microinjection of an α4 nAChR antagonist, DHβE, into the VP of eGFP (*n* = 8) and Chrna4 (*n* = 8) mice. **(G)** Contralateral PWT. *F*_(1, 14)_ = 3.38, *P* = 0.09. eGFP: *t* = 1.84, *P* = 0.09, saline vs. DHβE. Chrna4: *t* = 4.41, *P* = 0.0012, saline vs. DHβE. **(H)** Ipsilateral PWT. *F*_(1, 14)_ = 8.17, *P* = 0.012. eGFP: *t* = 0.18, *P* = 0.86, saline vs. DHβE. Chrna4: *t* = 4.22, *P* = 0.002, saline vs. DHβE. **(I)** Con*t*ralateral PWL. *F*_(1, 14)_ = 4.30, *P* = 0.06. eGFP: *t* = 2.15, *P* = 0.049, saline vs. DHβE. Chrna4: *t* = 5.08, *P* = 0.0003, saline vs. DHβE. **(J)** Ipsilateral PWL. *F*_(1, 14)_ = 25.33, *P* < 0.001. eGFP: *t* = 0.43, *P* = 0.067, saline vs. DHβE. Chrna4: *t* = 6.69, *P* < 0.001, saline vs. DHβE. **(K, L)** PWT on ei*t*her hind paw in eGFP and Chrna4 mice subjected to injec*t*ion of capsaicin (Cap) on hind paws and microinjec*t*ion of DHβE into the VP. **(K)** PWT in eGFP. Contralateral: *F*_(1.275, 8.927)_ = 38.78, *P* < 0.001; *t* = 7.14, *P* < 0.001, saline vs. Cap; *t* = 7.62, *P* < 0.001, Cap vs. Cap + DHβE; *t* = 4.41, *P* = 0.003, saline vs. Cap + DHβE. Ipsilateral: *F*_(1.621, 11.35)_ = 36.73, *P* < 0.001; *t* = 7.47, P = 0.004, saline vs. Cap; *t* = 5.13, *P* = 0.0027, Cap vs. Cap + DHβE; *t* = 4.47, *P* = 0.003, saline vs. Cap + DHβE. **(L)** PWT in Chrna4. Con*t*ralateral: *F*_(1.210, 8.473)_ = 24.09, *P* < 0.001; *t* = 10.92, *P* < 0.001, saline vs. Cap; *t* = 6.08, *P* = 0.001, Cap vs. Cap + DHβE; *t* = 1.2, *P* = 0.27, saline vs. Cap + DHβE. Ipsilateral: *F*_(1.837, 12.86)_ = 20.38, *P* < 0.001; *t* = 5.54, *P* = 0.002; saline vs. Cap; *t* = 6.36, *P* = 0.001, Cap vs. Cap + DHβE; *t* = 0.68, *P* = 0.52, saline vs. Cap + DHβE. **(M, N)** PWT and PWL in eGFP and Chrna4 mice before and af*t*er SNI surgery and injection of DHβE into the VP. **(M)** PWT in eGFP: *F*_(1.088, 7.614)_ = 47.03, *P* < 0.001; *t* = 7.61, *P* < 0.001, saline vs. SNI; *t* = 5.74, *P* = 0.001, SNI vs. SNI + DHβE; *t* = 6.12, *P* = 0.001, saline vs. SNI + DHβE. PWT in Chrna4: *F*_(1.574, 11.02)_ = 69.35, *P* < 0.001; *t* = 9.41, *P* < 0.001, saline vs. SNI; *t* = 11.32, *P* < 0.001, SNI vs. SNI + DHβE; *t* = 2.94, *P* = 0.022, saline vs. SNI + DHβE. **(N)** PWL in eGFP. *F*_(1.931, 13.52)_ = 92.56, *P* < 0.001; *t* = 14.91, *P* < 0.001, saline vs. SNI; *t* = 5.07, *P* = 0.002, SNI vs. SNI + DHβE; *t* = 7.96, *P* < 0.001, saline vs. SNI + DHβE. PWL in Chrna4. *F*_(1.276, 8.935)_ = 45.22, *P* < 0.001; *t* = 7.26, *P* < 0.001, saline vs. SNI; *t* = 10.9, *P* < 0.001, SNI vs. SNI + DHβE; *t* = 2.93, *P* = 0.022, saline vs. SNI + DHβE. **(O, P)** PWT and PWL in eGFP and Chrna4 SNI mice before and after intraperi*t*oneal injection of naloxone (Nal). **(O)** PWT in eGFP. *F*_(1.702, 11.91)_ = 24.44, *P* < 0.001; *t* = 5.74, *P* = 0.001, SNI vs. DHβE; *t* = 0.66, *P* = 0.53, SNI + DHβE vs. SNI + DHβE + Nal. PWT in Chrna4. *F*_(1.457, 10.20)_ = 68.15, *P* < 0.001; *t* = 11.32, *P* < 0.001, SNI vs. DHβE; *t* = 0.67, *P* = 0.53, SNI + DHβE vs. SNI + DHβE + Nal. **(P)** PWL in eGFP: *F*_(1.279, 8.951)_ = 17.51, *P* = 0.0016; *t* = 5.07, *P* = 0.003, SNI vs. DHβE; *t* = 0.79, *P* = 0.45, SNI + DHβE vs. SNI + DHβE + Nal. PWT in Chrna4. *F*_(1.289, 9.020)_ = 31.67, *P* < 0.001; *t* = 10.9, *P* < 0.001, SNI vs. DHβE; *t* = 1.67, *P* = 0.14, SNI + DHβE vs. SNI + DHβE + Nal. **(Q, R)** PWT and PWL in eGFP and Chrna4 mice before and after SNI surgery and injec*t*ion of RJR-2403 (RJR) into *t*he VP. **(Q)** PWT in eGFP. *F*_(1.002, 7.017)_ = 60.09, *P* < 0.001; *t* = 7.60, *P* < 0.001, saline vs. SNI; *t* = 1.89, *P* = 0.10, SNI vs. SNI + RJR. PWT in Chrna4. *F*_(1.050, 7.353)_ = 115.8, *P* < 0.001; *t* = 9.41, *P* < 0.001, saline vs. SNI; *t* = 5.56, *P* < 0.001, SNI vs. SNI + RJR. **(R)** PWL in eGFP: *F*_(1.084, 7.586)_ = 181.6, *P* < 0.001; *t* = 14.91, *P* < 0.001, saline vs. SNI; *t* = 6.88, *P* = 0.002, SNI vs. SNI + RJR. PWL in Chrna4: *F*_(1.119, 7.832)_ = 71.85, *P* < 0.001; *t* = 7.26, *P* < 0.001, saline vs. SNI; *t* = 9.73, *P* < 0.001, SNI vs. SNI + RJR. * *P* < 0.05; ** *P* < 0.01; ns not significant. Two-way ANOVAs with Bonferroni tests for **(D)**. Two-way repea*t*ed measures ANOVAs for **(E, F)**. Two-way repea*t*ed measures ANOVAs for **(G**–**J)**. One-way repeated measuremen*t*s ANOVAs with Bonferroni *t*es*t*s for **(K**–**R)**. Da*t*a are available in S1 Data as a par*t* of Supporting informa*t*ion.

The data in Fig 5 show that in SNI mice, mRNA levels of *Chrna4* gene in VP glutamatergic neurons was downregulated. To determine whether these pathophysiological changes are associated with hyperalgesia in SNI mice, we constructed a Cre-dependent viral vector carrying *Chrna4* gene (AAV-hSyn-DIO-Chrna4-eGFP) (Chrna4) to overexpress α4 nAChR in particular neuron types [28]. We injected this viral vector into the VP of Vglut2-Cre mice to overexpress α4 nAChR subunit in VP glutamatergic neurons and injected AAV-hSyn-DIO-eGFP into the VP of Vglut2-Cre mice as a control (Fig 7A). We confirmed that the great majority of eGFP-positive neurons in the VP were labeled by CaMKII antibody (Fig 7B and 7C). The qRT-PCR analysis of laser-captured Chrna4-eGFP-positive neurons in the VP showed that one month after the injection of the viral vector, *Chrna4* mRNA was upregulated by more than 10-folds without affecting *Chrna7* mRNA, relative to those in eGFP neurons (Fig 7D). Using brain slice patch-clamp technique, we observed that 50 μM RJR-2403-evoked inward currents in Chrna4-eGFP-positive neurons were 5-folds of those in eGFP-positive neurons (S13F–S13I Fig). These data indicate that AAV-hSyn-DIO-Chrna4-eGFP can be used to up-regulate α4 nAChR subunit in Cre-positive neurons.

After injection of AAV-hSyn-DIO-Chrna4-eGFP (Chrna4) or AAV-hSyn-DIO-eGFP (eGFP) into the VP of Vglut2-Cre mice, these mice did not show alterations in either mechanical or thermal threshold up to 6 weeks (Fig 7E and 7F). Although administration of α4 nAChR antagonist, DHβE (0.3 μM, 200 nl), into the VP did not change either mechanical or thermal threshold in eGFP mice (Fig 7G–7J), it elevated both mechanical and thermal thresholds in Chrna4 mice (Fig 7G–7J). These data provide evidence supporting that the viral vector enhances the function of α4 nAChRs in VP glutamatergic neurons at the behavioral setting and α4 nAChR on VP glutamatergic neurons mediate analgesic effect of α4 nAChR antagonist in the VP.

To understand whether upregulation of α4 nAChRs on VP glutamatergic neurons affect severity of hyperalgesia in pathological pain states, we established inflammatory pain (subcutaneous injection of 20 μl capsaicin into the lower hind leg) and neuropathic pain (SNI on the left side) mouse models. We observed that in either pain model, eGFP and Chrna4 mice exhibited similar levels of mechanical PWT and thermal PWL (Fig 7K–7N); after capsaicin injection into the hind paw, eGFP and Chrna4 mice exhibited similarly lower levels of mechanical PWT and thermal PWL, which were significantly elevated after microinjection of DHβE (0.3 μM, 200 nl) into the VP (Fig 7K and 7L); 2 weeks after SNI surgery, eGFP and Chrna4 mice showed lower mechanical PWT and thermal PWL, and DHβE (0.3 μM, 200 nl) injected into the VP elevated mechanical PWT more dramatically in Chrna4 mice than eGFP mice (Fig 7M), while elevated thermal PWL to a similar level in Chrna4 and eGFP mice (Fig 7N). As qRT-PCR assay of *Chrna4* mRNA in VP glutamatergic neurons showed that after SNI, the mRNA level of *Chrna4* gene in VP glutamatergic neurons was higher in Chrna4 mice than eGFP mice (S13J Fig), these data support that upregulation of α4 nAChRs in VP glutamatergic neurons did not affect the severity of hyperalgesia in pain states, while may be involved in analgesic effect of α4 nAChR antagonist on mechanical pain, but not on thermal pain. Interestingly, the analgesic effect of DHβE injected into the VP was not affected by intraperitoneally administered naloxone (5 mg/kg), an antagonist for opioid receptors (Fig 7O and 7P); injection of RJR-2403 exacerbated hyperalgesia in SNI Chrna4 mice (Fig 7Q and 7R). These data suggest that downregulation of α4 nAChR subunit in VP glutamatergic neurons may not contribute to hyperalgesia in neuropathic pain, but upregulation of these receptors may provide a means to enhance analgesic effects of α4 nAChR antagonist on mechanical allodynia in neuropathic pain.

### Upregulation of α7 nAChR subunit in VP glutamatergic neurons enhanced analgesic effect of α7 nAChR antagonist in the VP on neuropathic pain

**Fig 8:**
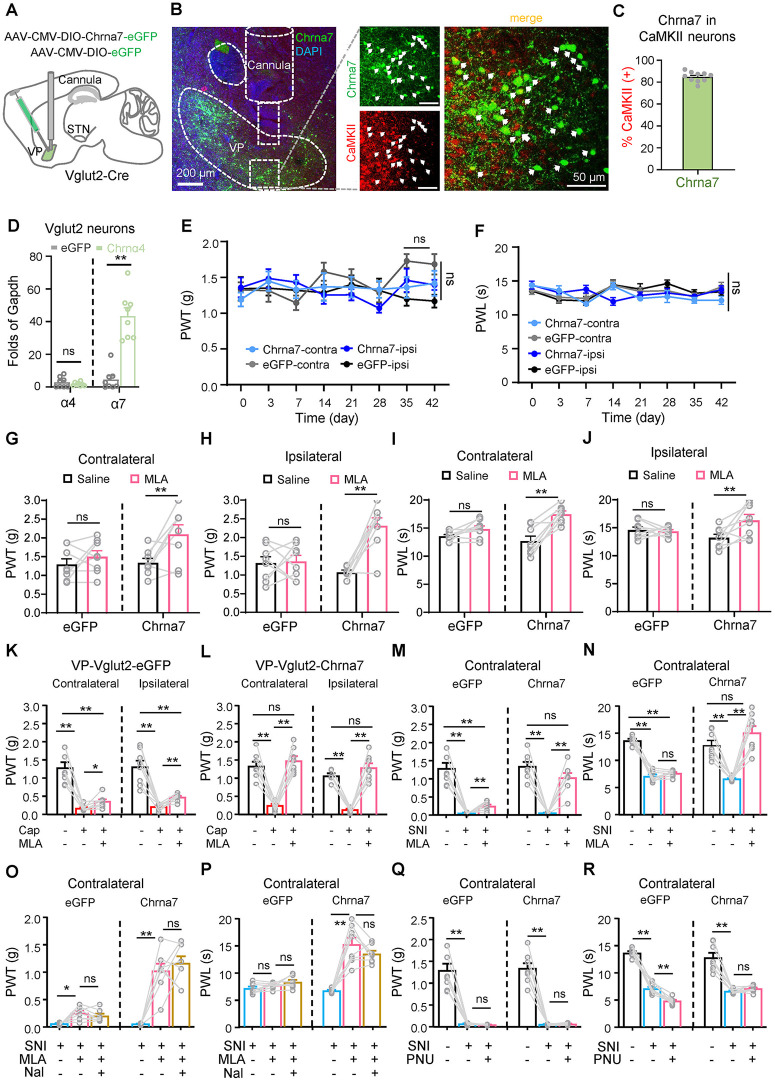
Up-regulation of α7 nAChR subunit in VP glutamatergic neurons enhances analgesic effects of α7 nAChR antagonist injection into the VP. **(A)** Schematic diagram for injection of AAV-CMV-DIO-EGFP-Chrna7 (Chrna7) or AAV-CMV-DIO-eGFP (eGFP) and implantation of a cannula into the right VP of Vgat-Cre mice. **(B, C)** Representative images and summary showing eGFP expression (green) in GABAergic neurons (red) 3 weeks after virus injection. Chrna7 and CaMKII co-expressing neurons accounted for 84.9% of Chrna7 neurons. Summary data were from 10 slices of 5 mice. **(D)** The mRNA levels of *α4* and *α7* subunits in VP glutamatergic neurons in Chrna7 (*n* = 8) and eGFP (*n* = 8) mice were quantified with qRT-PCR assay. Interaction *F*_(1, 28)_ = 49.62, *P* < 0.001; Receptor, *F*_(1, 28)_ = 58.31, *P* < 0.001; Group, *F*_(1, 28)_ = 44.28, *P* < 0.001. *α4*: *t* = 0.27, *P* = 0.78; *α7*: *t* = 9.69, *P* < 0.001. **(E, F)** Time courses of PWT and PWL on either hind paw in mice subjec*t*ed *t*o injection of AAV-CMV-DIO-Chrna7-eGFP (Chrna4) or AAV-CMV-DIO-eGFP (eGFP). **(E)** PWT. Interaction, *F*_(21, 196)_ = 1.20, *P* = 0.25; Group, *F*_(3, 28)_ = 1.65, *P* = 0.29; Time, *F*_(5.491, 153.8)_ = 1.02, *P* = 0.41. **(F)** PWL. *F*_(21, 196)_ = 1.95, *P* = 0.01; Group, *F*_(3, 28)_ = 1.30, *P* = 0.29; Time, *F*_(5.831, 163.3)_ = 3.36, *P* = 0.004. **(G**–**J)** PWT and PWL on either hind paw before and after microinjection of an α7 nAChR antagonist, methyllycaconitine (MLA), into the VP of eGFP and Chrna7 mice. **(G)** Contralateral PWT. *F*_(1, 14)_ = 3.15, *P* = 0.097. eGFP: *t* = 0.96, *P* = 0.35, saline vs. MLA. Chrna7: *t* = 3.46, *P* = 0.007, saline vs. MLA. **(H)** Ipsilateral PWT. *F*_(1, 14)_ = 20.26, *P* < 0.001. eGFP: *t* = 0.21, *P* = 0.83, saline vs. MLA. Chrna7: *t* = 5.49, *P* < 0.001, saline vs. MLA. **(I)** Con*t*ralateral PWL. *F*_(1, 14)_ = 10.88, *P* = 0.005. eGFP: *t* = 1.70, *P* = 0.11, saline vs. MLA. Chrna7: *t* = 6.36, *P* < 0.001, saline vs. MLA. **(J)** Ipsila*t*eral PWL. *F*_(1, 14)_ = 6.78, *P* = 0.02. eGFP: *t* = 0.25, *P* = 0.81, saline vs. MLA. Chrna7: *t* = 3.43, *P* = 0.008, saline vs. MLA. **(K, L)** PWT on either hind paw in eGFP and Chrna7 mice subjec*t*ed *t*o injection of capsaicin (Cap) on hind paws and microinjection of MLA in*t*o *t*he VP. **(K)** PWT in eGFP. Contralateral: *F*_(1.199, 8.393)_ = 43.81, *P* < 0.001; *t* = 7.02, *P* < 0.001, saline vs. Cap; *t* = 3.26, *P* = 0.014, Cap vs. Cap + MLA; *t* = 6.50, *P* < 0.001, saline vs. Cap + MLA. Ipsilateral: *F*_(1.094, 7.655)_ = 36.24, *P* < 0.001; *t* = 6.48, *P* = 0.001, saline vs. Cap; *t* = 6.05, *P* = 0.001, Cap vs. Cap + MLA; *t* = 5.42, *P* = 0.001, saline vs. Cap + MLA. **(L)** PWT in Chrna7. Contrala*t*eral: *F*_(1.199, 8.393)_ = 43.81, *P* < 0.001; *t* = 7.02, *P* < 0.001, saline vs. Cap; *t* = 3.26, *P* = 0.014, Cap vs. Cap + MLA; *t* = 6.50, *P* < 0.001, saline vs. Cap + MLA. Ipsilateral: *F*_(1.094, 7.655)_ = 36.24, *P* < 0.001; *t* = 6.48, *P* = 0.001, saline vs. Cap; *t* = 6.05, *P* = 0.001, Cap vs. Cap + MLA; *t* = 5.42, *P* = 0.001, saline vs. Cap + MLA. **(M, N)** PWT and PWL in eGFP and Chrna7 mice before and af*t*er SNI surgery and injec*t*ion of MLA into the VP. **(M)** PWT in eGFP. *F*_(1.038, 7.269)_ = 44.82, *P* < 0.001; *t* = 8.34, *P* < 0.001, saline vs. SNI; *t* = 3.94, *P* = 0.006, SNI vs. SNI + MLA; *t* = 5.58, *P* = 0.002, saline vs. SNI + MLA. PWT in Chrna7. *F*_(1.723, 12.06)_ = 47.91, *P* < 0.001; *t* = 10.98, *P* < 0.001, saline vs. SNI; *t* = 7.57, *P* < 0.001, SNI vs. SNI + MLA; *t* = 1.95, *P* = 0.09, saline vs. SNI + MLA. **(N)** PWL in eGFP: *F*_(1.517, 10.62)_ = 134.0, *P* < 0.001; *t* = 12.02, *P* < 0.001, saline vs. SNI; *t* = 1.73, *P* = 0.13, SNI vs. SNI + MLA; *t* = 13.8, *P* < 0.001, saline vs. SNI + MLA. PWL in Chrna7: *F*_(1.395, 9.766)_ = 21.33, *P* < 0.001; *t* = 6.52, *P* < 0.001, saline vs. SNI; *t* = 6.71, *P* < 0.001, SNI vs. SNI + MLA; *t* = 1.36, *P* = 0.22, saline vs. SNI + MLA. **(O, P)** PWT and PWL in eGFP and Chrna7 SNI mice before and after injection of MLA into the VP and in*t*raperitoneal injec*t*ion of naloxone (Nal). **(O)** PWT in eGFP: *F*_(1.823, 12.76)_ = 10.75, *P* = 0.002; *t* = 3.94, *P* = 0.011, SNI vs. SNI + MLA; *t* = 1.35, *P* = 0.22, SNI + MLA vs. SNI + MLA + Nal. PWT in Chrna7: *F*_(1.769, 12.39)_ = 36.16, *P* < 0.001; *t* = 7.57, *P* < 0.001, SNI vs. SNI + MLA; *t* = 0.82, *P* = 0.44, SNI + MLA vs. SNI + MLA + Nal. **(P)** PWL in eGFP: *F*_(1.390, 9.729)_ = 3.61, *P* = 0.08; *t* = 1.73, *P* = 0.24, SNI vs. SNI + MLA; *t* = 1.57, *P* = 0.24, SNI + MLA vs. SNI + MLA + Nal. PWT in Chrna7: *F*_(1.301, 9.109)_ = 38.50, *P* < 0.001; *t* = 6.71, *P* < 0.001, SNI vs. SNI + MLA; *t* = 1.57, *P* = 0.29, SNI + MLA vs. SNI + MLA + Nal. **(Q, R)** PWT and PWL in eGFP and Chrna7 mice before and af*t*er SNI surgery and injection of PNU-282987 (PNU) in*t*o *t*he VP. **(Q)** PWT in eGFP: *F*_(1.005, 7.034)_ = 67.99, *P* < 0.001; *t* = 8.37, *P* < 0.001, Control vs. SNI; *t* = 1.94, *P* = 0.18, SNI vs. SNI + PNU. PWT in Chrna7: *F*_(1.011, 7.080)_ = 116.5, *P* < 0.001; *t* = 10.98, *P* < 0.001, Con*t*rol vs. SNI; *t* = 0.86, *P* = 0.66, SNI vs. SNI + PNU. **(R)** PWL in eGFP: *F*_(1.550, 10.85)_ = 217.4, *P* < 0.001; *t* = 12.02, *P* < 0.001, Control vs. SNI; *t* = 6.16, *P* < 0.001, SNI vs. SNI + PNU. PWL in Chrna7: *F*_(1.074, 7.517)_ = 37.42, *P* < 0.001; *t* = 6.52, *P* < 0.001, Control vs. SNI; *t* = 2.18, *P* = 0.13, SNI vs. SNI + PNU. * *P* < 0.05; ** *P* < 0.01; ns not significant. Two-way ANOVAs wi*t*h Bonferroni *t*ests for **(D)**. Two-way repea*t*ed measures ANOVAs for **(E, F)**. Two-way repeated measures ANOVAs for **(G**–**J)**. One-way repea*t*ed measuremen*t*s ANOVAs wi*t*h Bonferroni tes*t*s for **(K**–**R)**. Data are available in S1 Data as a part of Supporting information.

To determine whether downregulation of α7 nAChR subunit in VP glutamatergic neurons in SNI mice is associated with hyperalgesia, we constructed a Cre-dependent viral vector carrying genes of *α7* nAChR subunit (AAV-hSyn-DIO-Chrna7-eGFP) (Chrna7). We injected this viral vector into the VP of Vglut2-Cre mice to overexpress α7 nAChRs in VP glutamatergic neurons and injected AAV-hSyn-DIO-eGFP into the VP of Vglut2-Cre mice as a control (Fig 8A). We confirmed that the great majority of eGFP-positive neurons in the VP were labeled by CaMKII antibody (Fig 8B and 8C). One month after the injection of the viral vector into the VP, *Chrna7* mRNA in VP glutamatergic neurons was dramatically upregulated without affecting *Chrna4* mRNA, relative to those in eGFP neurons (Fig 8D). Using brain slice patch-clamp technique, we observed that PNU282987-evoked inward currents in Chrna7-eGFP-positive neurons were larger than those in eGFP-positive neurons (S13K–S13O Fig). These data indicate that AAV-hSyn-DIO-Chrna7-eGFP is capable of upregulating α7 nAChR subunit in Cre-positive neurons.

After injecting AAV-hSyn-DIO-Chrna7-eGFP (Chrna7) or AAV-hSyn-DIO-eGFP (eGFP) into the VP of Vglut2-Cre mice, we measured mechanical and thermal thresholds in Chrna7 and eGFP mice, and observed that Chrna7 mice did not show reduction in either mechanical or thermal threshold up to 6 weeks (Fig 8E and 8F). Although administration of α7 nAChR antagonist, MLA (2 μM, 200 nl), into the VP did not change either mechanical or thermal threshold in eGFP mice (Fig 8G–8J), it elevated both mechanical (Fig 8G and 8H) and thermal (Fig 8I and 8J) thresholds in Chrna7 mice. In eGFP and Chrna7 mice, capsaicin injection onto the lower part of the leg (Fig 8K and 8L) and SNI surgery (Fig 8M and 8N) reduced PWT and PWL to a similar level, but the analgesic effect of MLA injection into the VP was enhanced in Chrna7 mice subjected to SNI surgery, relative to eGFP mice subjected to SNI surgery. This is consistent with the upregulation of α7 nAChR subunit in VP glutamatergic neurons in Chrna7 mice subjected to SNI surgery (S13O Fig). Additionally, naloxone (5 mg/kg) did not block analgesic effect of MLA injection into the VP (Fig 8O and 8P), while injection of PNU282987 (300 μM 200 nl) into the VP did not further reduce pain thresholds in Chrna7 and eGFP SNI mice (Fig 8Q and 8R).

These data suggest that α7 nAChRs in VP glutamatergic neurons may not alter the severity of hyperalgesia in inflammatory pain and neuropathic pain states, but upregulation of α7 nAChR in VP glutamatergic neurons may provide a means to enhance analgesic effects of α7 nAChR antagonist administered into the VP on mechanical allodynia and thermal hypersensitivity.

In preceding paragraphs, our data suggest that upregulation of α4 nAChRs in GABAergic neurons may contribute to pathophysiology in neuropathic pain; downregulation of α4 and α7 nAChRs in glutamatergic neurons may not alter the severity of hyperalgesia in pain states. Therefore, in neuropathic pain, hyperexcitability in VP cholinergic neurons may lead to increased ACh release in the VP which may activate the upregulated α4 nAChRs on GABAergic neurons and confer both mechanical and thermal hyperalgesia, while downregulation of α4 nAChRs on VP glutamatergic neurons may act as a compensatory mechanism to reduce severity of hyperalgesia.

### Contralateral projections from VP glutamatergic and GABAergic neurons contribute to ipsilateral hypersensitivity following activation of VP cholinergic neurons

Our previous study [19] and data in the present study revealed that VP cholinergic neurons regulate pain thresholds on both hind paws. Although we demonstrated that the VP-basolateral amygdala cholinergic projection modulates pain thresholds on the contralateral side [19], it remains unknown which downstream components of VP cholinergic neurons modulate mechanical and thermal thresholds on the ipsilateral side. Similar to our previous study [19], we observed that stimulation of VP cholinergic neurons on one side resulted in excitation of cholinergic and non-cholinergic neurons in the VP on both sides (Fig 1J–1L). We wondered whether there exist contralateral projections from VP neurons. To prove this, we injected AAV-hSyn-DIO-mGFP-Synaptophysin-mRuby into the right VP of ChAT-Cre, Vgat-Cre, and Vglut2-Cre mice to respectively label cholinergic, GABAergic, and glutamatergic neuronal somata and fibers with mGFP and their synaptic terminals with mRuby (S14–S16 Figs). We observed most downstream nuclei of the labeled VP neurons were in the ipsilateral hemisphere; VP cholinergic neurons did not project to the contralateral side (S14A–S14D Fig); VP GABAergic (S15A–S15D Fig) and glutamatergic (S16A–S16D Fig) neurons projected to several nuclei on the contralateral side, including the contralateral VP (S15 and S16 Figs).

To confirm that VP neurons innervate neurons in the contralateral VP, we injected an anterograde transsynaptic viral vector (AAV1-hSyn-DIO-Flp) into the right VP and AAV-hSyn-fDIO-mCherry into the left VP in Vglut2-Cre (S17A Fig) and Vgat-Cre (S17F Fig) mice. The combination of these viral vectors enables mCherry-labeling of Cre-positive neurons in the left VP that receive innervation from the right VP. Our data showed that 88.24% and 82.52% of mCherry-positive neurons in the VP in Vglut2-Cre and Vgat-Cre mice were, respectively, stained by CaMKII- and GABA-antibody (S17B, S17C, S17G and S17H Fig). Meanwhile, mCherry-positive neurons in the VP distributed on several parasagittal planes (S17D, S17E, S17I, and S17J Fig). These data suggest that VP glutamatergic and GABAergic neurons receive direct innervation from the contralateral VP.

The above evidence suggests that VP cholinergic neurons may regulate contralateral VP neurons likely through non-cholinergic neurons projecting to the contralateral side. To address whether stimulation of VP cholinergic neurons modulates non-cholinergic neurons, we applied optogenetic stimulation of VP cholinergic neurons and examined its effects on the activity of VP non-cholinergic neurons (S18A and S18B Fig). We observed that either 5 and 10 s blue light stimulation (460 nm, 5 ms, 20 Hz, 2 mW) increased spontaneous firing rate in most VP non-cholinergic neurons (S18C–S18H Fig). We wondered whether contralateral projection of non-cholinergic (glutamatergic and GABAergic) neurons mediate part of function of VP cholinergic neurons on modulating mechanical and thermal thresholds. To address this issue, we combined optogenetic stimulation of VP cholinergic neurons and chemogenetic inhibition of the contralateral projections from VP glutamatergic and GABAergic neurons, and examined pain thresholds on either hind paw (S19A Fig). To fulfill this goal, we injected AAV-EF1α-DIO-ChR2-eYFP and implanted an optical fiber into the right VP of ChAT-Cre mice for optogenetic stimulation of VP cholinergic neurons (S19B, S19C, and S19E Fig). In these mice, we also injected viral vector (AAV-CaMKII-hM4Di-mCherry or AAV-GAD67-hM4Di-mCherry) into the right VP to transfect hM4Di into CaMKII neurons or GABAergic neurons (S19D and S19F Fig). By implanting a cannula above the left VP and injecting CNO through the cannula, we may achieve chemogenetic inhibition of contralateral projections of VP glutamatergic or GABAergic neurons (S19C and S19E Fig). These three viral vectors transfected cholinergic neurons, glutamatergic neurons or GABA neurons in the VP with high specificity (> 80%) (S19B, S19D, and S19F–S19J Fig). We observed that optogenetic stimulation (5 ms, 20 Hz, 21.72 mW/mm^2^) of VP cholinergic neurons in the right hemisphere reduced both mechanical and thermal thresholds on either hind paw (S19K–S19R Fig). After injection of CNO (3 μM 200 nl) into the left VP to inhibit the contralateral projections of either glutamatergic neurons (S19K–S19N Fig) or GABAergic neurons (S19O–S19R Fig) in the right VP, optogenetic stimulation of cholinergic neurons (5 ms, 20 Hz, 21.72 mW/mm^2^) in the right VP reduced mechanical and thermal thresholds on the contralateral side, but not those on the ipsilateral side (S19K–S19R Fig). Interestingly, in SNI mice, chemogenetic inhibition of the contralateral projection of VP glutamatergic (S19S and S19T Fig) and GABAergic neurons (S19U and S19V Fig) robustly elevated mechanical and thermal thresholds on the ipsilateral hind paw in mice subjected to SNI surgery on the ipsilateral side. Therefore, the contralateral projections from VP glutamatergic and GABAergic neurons may be among major contributors mediating pain modulation on the ipsilateral side by VP cholinergic neurons.

## Discussion

Our previous studies showed that STN neurons and VP cholinergic neurons are involved in modulation of acute and chronic pain states [19,22,23]. Optogenetic stimulation of either neurons reduces pain thresholds on both sides [19,22,23]. These neurons also respond to mechanical and thermal stimulation with enhanced activity [19,23]. However, these studies did not address whether STN glutamatergic neurons innervate VP cholinergic neurons and whether these synaptic connections contribute to pain signaling and modulation. In this study, we delineated a monosynaptic glutamatergic projection from STN glutamatergic neurons to VP cholinergic neurons. Furthermore, STN neurons control pain responses in VP cholinergic neurons, and VP cholinergic neurons significantly contributed to pain modulation by the STN-VP projection. These data provide a pain modulation pathway connecting the basal ganglia with the forebrain and add VP cholinergic neurons as a downstream neuron type of the STN in mediating nociception and chronic pain. Collectively, activation and hyperexcitability of STN neurons and VP cholinergic neurons in nociception and chronic pain [19,23] suggest that the pathway consisting of STN glutamatergic neurons, VP cholinergic neurons, and the STN^Glu^-VP^ChAT^ projection may be a promising target for the treatment of chronic pain.

Although nicotinic receptor subunits distribute broadly in the central nervous system [29–31], the cell-specific expression profile of these receptors has been poorly addressed. Several studies defined the existence of α7β2 nAChRs on cholinergic neurons in the medial septum, NBM, and diagonal band of Broca [32–37], but did not mention nAChR receptor subtypes on non-cholinergic neurons. In the present study, we utilized a cell-specific technique to characterize nAChRs in VP neurons. We revealed that nAChRs in VP cholinergic, glutamatergic, and GABAergic neurons contain α4 and α7 subunits. Although β3 and β4 subunit mRNAs were detected in these neurons, their quantities were much less and more variable. Because of technical limitation of the present study, we could not deduce how nAChR pentamers were assembled in each neuron types. Nevertheless, we provided functional evidence showing that these nAChRs were able to be activated by widely used nAChR agonists with selectivity to α4-containing and α7-containing nAChRs. The evidence demonstrates that α4 and α7 nAChRs can be targets to regulate VP neurons.

In the physiological condition, α4 and α7 nAChRs in the VP may not contribute to the gating mechanism of pain because blocking these receptors altered neither mechanical nor thermal thresholds in mice. But several changes in VP cholinergic circuitry may differentially perturb the gating of responses to mechanical and thermal stimulation. For instance, activation of α4 nAChRs reduced both mechanical and thermal thresholds on either hind paw, activation of α7 nAChRs reduced only mechanical threshold on either hind paw. In neuropathic pain, VP cholinergic neurons became hyperactive and released more ACh into the VP in response to mechanical stimulation. This may be relevant to hyperalgesia because activation of VP cholinergic neurons caused reduction in mechanical and thermal threshold, and this effect was blocked by nicotinic receptor antagonists. Although upregulation of α4 and α7 nAChRs in VP glutamatergic neurons and downregulation of α7 nAChRs in VP GABAergic neurons did not change pain thresholds, downregulation of α4 nAChRs on VP GABAergic neurons moderately reduced mechanical and thermal thresholds and mitigated hypersensitivity to mechanical and thermal stimulation in SNI mice. These data are inconsistent with our finding that blocking α4 nAChRs in the VP did not change pain thresholds in naive mice. Longer time period of neural circuit perturbation following downregulation of α4 nAChRs may be followed by neuroadaptation, which can lead to hypersensitivity in physiological condition, but counteract development of hyperalgesia in neuropathic pain. Further investigations are needed to elucidate the underlying mechanisms.

In the VP, both cholinergic and non-cholinergic neurons are hyperactive in chronic pain [19]. In the present study, we divided non-cholinergic neurons into GABAergic and glutamatergic neurons and found that cholinergic and GABAergic neurons were hyperactive, while glutamatergic neurons were hypoactive in SNI mice. We observed that most non-cholinergic neurons expressed nicotinic receptors, and activation of VP cholinergic neurons increased firing rate in most non-cholinergic neurons. Additionally, mechanical stimulation on hind paws increased ACh release in the VP in SNI mice more than that in sham mice. These may be inconsistent with hypoactivity in VP glutamatergic neurons in SNI mice. With qRT-PCR assay and whole-cell patch-clamp recording, we revealed that in SNI mice, α4 subunit was downregulated in VP glutamatergic neurons, but upregulated in GABAergic neurons; α7 subunit was downregulated in both cholinergic and glutamatergic neurons. These may explain at least partially the hyperactivity of GABAergic neurons and hypo-activity of glutamatergic neurons in the VP in SNI mice. As in the VP, GABAergic and glutamatergic neurons account for 70% and 15% of neuronal population, it is reasonable to assume that in our previous study, more GABAergic neurons were sampled than glutamatergic neurons during patch-clamp recording [19]. Therefore, hyperactive GABAergic neurons may overbalance hypoactive glutamatergic neurons, resulting in hyperactivity in non-cholinergic neurons. In fact, modifications in VP circuitry should not be limited to nicotinic receptors. These may explain why nicotinic receptor antagonists differentially affect spontaneous and evoked firing in VP cholinergic, GABAergic and glutamatergic neurons in SNI mice.

To understand the potential contribution of distinct modifications of α4 and α7 nAChRs in VP GABAergic and glutamatergic neurons to neuropathic pain, we used a cell-specific gene delivery strategy combining Cre-dependent AAV-viral vectors with Cre-mouse lines to reverse these changes. Interestingly, downregulation of α4, but not α7, nAChRs in VP GABAergic neurons significantly reduced pain thresholds, while SNI did not further reduce pain thresholds. The resulting pain thresholds in these SNI mice were significantly higher than SNI mice in which α4 nAChRs in VP GABAergic neurons were not downregulated. Moreover, injection of α4 nAChR agonists (RJR-2403 and varenicline) into the VP did not further reduce mechanical and thermal thresholds in neuropathic pain state. These data suggest that α4 nAChRs in VP GABAergic neurons determine the severity of hyperalgesia in neuropathic pain. After α4 nAChRs in VP GABAergic neurons were downregulated, injection of α4 nAChR antagonist still elevated pain thresholds in neuropathic pain state to an extent seen in mice whose α4 nAChRs in VP GABAergic neurons were intact. This fact suggests that α4 nAChRs in the VP other than those in GABAergic neurons may be targets for α4 nAChR antagonist to confer analgesic effects. α4 nAChRs in VP glutamatergic neurons may be among these receptors because upregulation of α4 nAChRs in VP glutamatergic neurons enhanced the analgesic effect of α4 nAChR antagonist in the VP.

In contrast to α4 nAChRs in VP GABAergic neurons, reversal (upregulation) of α4 and α7 nAChR deficiency in VP glutamatergic neurons leads to several consequences. Although it did not alter mechanical and thermal thresholds in SNI mice, it enhanced analgesic effect of α4 and α7 nAChR antagonist in SNI mice, which is independent of the endogenous opioid system. Interestingly, after α4 nAChRs in VP glutamatergic neurons were upregulated, α4 nAChR agonist further reduced pain thresholds in SNI mice; while after α7 nAChRs in VP glutamatergic neurons were upregulated, α7 nAChR agonist did not change pain thresholds in SNI mice. These data suggest that α4 nAChRs but not α7 nAChRs in VP glutamatergic neurons may be involved in hyperalgesia following enhanced ACh release in the VP in SNI mice. Downregulation of α4 nAChRs in VP glutamatergic neurons may be a compensatory mechanism in neuropathic pain.

This study reveals that cholinergic circuit in the VP in one hemisphere modulates mechanical and thermal thresholds on hind paws on both sides. As basal forebrain cholinergic neurons send projections to other cortical and subcortical nuclei [5,12,13,17,25,38], pain modulation effects of VP cholinergic neurons may be mediated by their long-range projections and local circuits. Our previous study reports that the VP-BLA cholinergic projection modulate pain thresholds on the contralateral side [19]. In the present study, we demonstrated that in the VP of one hemisphere, some non-cholinergic neurons innervated by cholinergic neurons projected to the contralateral VP, and activation of these non-cholinergic neurons was required for stimulation of cholinergic neurons to reduce mechanical and thermal thresholds on the ipsilateral side. Therefore, VP cholinergic neurons may modulate pain thresholds on both sides, while their long-range projection to the BLA and innervation to VP noncholinergic neurons projecting to the contralateral VP regulates pain thresholds on the contralateral and ipsilateral side, respectively.

There are several limitations in this study. We focused on the most abundant nAChR subunits (α4 and α7 subunits). But other subunits may be incorporated with these subunits to form functional receptors. Further sophisticated biophysical and molecular biological investigations are needed to clarify these puzzles. Although the viral vector possesses cell type- and receptor subtype-specificity, the manipulation of receptor subunit takes actions in several weeks, and in this process, unknown neuroadaptation in the VP might occur. This may be related to decrease in pain thresholds in mice whose α4 subunit in VP GABAergic neurons were downregulated. Solving this puzzle is essential to address why the moderate reduction of pain thresholds curbs severe reduction of pain thresholds in neuropathic pain.

In summary, this study demonstrates that VP cholinergic neurons are controlled by the STN-VP glutamatergic projection and are involved in representation and modulation of pain. By activating α4 and α7 nAChRs, VP cholinergic neurons innervate glutamatergic and GABAergic neurons in the VP. In neuropathic pain, these nAChRs are cell-specifically modified, among which upregulation of α4 nAChRs on VP GABAergic neurons importantly contributes to hyperalgesia. Therefore, the STN^Glu^-VP^ChAT^-VP^nonChAT^ pathway may play an important role in neuropathic pain, and α4 nAChRs on VP GABAergic neurons may be potential therapeutic targets for the treatment of neuropathic pain.

## Materials and methods

### Ethics statement

The experimental protocols in this study were approved by the Institutional Animal Care and Use Committee (IACUC) of Xuzhou Medical University under the Regulations for the Administration of Affairs Concerning Experimental Animals (1988) in China. The approval number is 202208S120. The use and care of animals were performed in the animal facility of Xuzhou Medical University managed by the Office of Laboratory Animal Resources of Xuzhou Medical University.

### Animals

C57BL/6J background ChAT-IRES-Cre (stock no. 006410), Vgat-Cre (stock no. 028862), and Vglut2-Cre (stock no. 016963) mice were purchased from the Jackson Laboratory. These transgenic mice were crossed with C57BL/6J mice and were bred in the animal facility of Xuzhou Medical University. The C57BL/6L mice and transgenic mice were group housed (no more than 4 per cage) with free access to water and food on a 12-hour light/dark cycle in an environment with stable temperature (21–23 °C) and humidity (40%–70%). Mice at least 8 weeks old were used for the experiments conducted during the light cycle. Efforts were made to minimize animal suffering and to reduce the number of mice used.

### Viral vectors

The AAV serotype 2/9 viral vectors were used for neuronal tracing, neuromodulation, ACh detection and gene regulation. AAV-EF1α-DIO-ChR2(H134R)-eYFP, AAV-EF1α-DIO-GCaMP6s, AAV-EF1α-DIO-eGFP, AAV-EF1α-DIO-eYFP, AAV-EF1α-DIO-hM3Dq-mCherry, AAV-EF1α-DIO-hM4Di-mCherry, AAV retro-hSyn-Flp, AAV-hSyn-fDIO-hM4Di-mCherry, AAV-EF1α-DIO-TVA-eGFP, AAV-EF1α-DIO-oRVG, RV-CVS-EnvA(∆G)-tdTomato, AAV-CMV-DIO-(EGFP-U6)-shRNA(Chrna4)-WPRE-hGH polyA, AAV-CMV-DIO-(EGFP-U6)-shRNA(Chrna7)-WPRE-hGH polyA, AAV-CMV-DIO-(eGFP-U6)-shRNA(scramble)-WPRE-hGH polyA, AAV-CMV-DIO-Chrna4-P2A-eGFP-WPREs, and AAV-CMV-DIO-Chrna7-P2A-eGFP-WPREs were purchased from Brain VTA (Wuhan, China). AAV1/2-hSyn-DIO-Flp, AAV2-hSyn-fDIO-mCherry, AAV-hSyn-DIO-mGFP-T2A-synaptophysin-mRuby was purchased from Brain Case (Wuhan, China). AAV2-hSyn-GACh3.0 were purchased from WZ Biosciences (Jinan, China). The titers of the AAV viral vectors are between 1 × 10^12^ to 5 × 10^12^ vg/ml. The titer of CVS is 3 × 10^8^ vg/ml. The titer of AAV1/2 is 3 × 10^13^ vg/ml.

### Mouse survival surgeries

Male mice were anesthetized with inhaled isoflurane (3% for induction, 1.5% for maintenance) and stabilized in a stereotaxic apparatus (RWD Life Science Co., Shenzhen, China) with a heating pad. The surgery was performed as described previously [22,23,39,40]. Viral vectors (0.2 µl) or drugs (0.2 µl) were injected with a microinjection pump (KD Scientific, Holliston, MA, USA) at a rate of 50 nl/min. The needle was kept in the injection site for 5–10 min after injection stopped, then, was slowly removed. The coordinates for viral injection were as follows: VP (AP, 0.5 mm; ML, ± 1.5 mm; DV, 4.8 mm). The optical fiber implants (200 μm in diameter, NA 0.37) (Inper, Hangzhou, China) were placed 200 µm above the injection site for optogenetic stimulation, but at the injection site for fiber photometry recording. The optical fiber implants were fixed to the skull with dental cement. For pharmacological manipulation, as the needle (30 G) for drug delivery is 500 μm longer than stainless steel guide cannulas (26 G) (RWD Life Science, Shenzhen, China), the cannula was implanted 500 μm above the viral injection site in the VP.

Meloxicam (4 mg/kg) was subcutaneously administered once per day for 3 days’ postoperative pain relief. The mice subjected to viral injection were allowed to recover for at least 3 weeks before the behavioral tests, optogenetic or pharmacological manipulations, electrophysiological recording, and morphological assays. The mice subjected to cannula implantation were allowed to recover for at least one week.

### Fiber photometry

Fiber photometry was performed with an instrument (ThinkerTech, Nanjing, China) to monitor GCaMP6 (a green-fluorescent calcium sensor) or eYFP signal in VP ChAT neurons and GACh (a green-fluorescent sensor for acetylcholine) or eYFP signal in the VP [41,42]. 5–6 mice in each group were examined. We set 470 nm excitation light for green fluorescence to 20–40 μW and an appropriate gain to a level that gave a background signal of 3 units without any light inputs. After we connected an input cable to the optical fiber implanted in the VP with a ceramic sleeve, the instrument read the total fluorescent signals. The difference between the total signal and the background signal was used as the baseline GCaMP6 signal. At the meantime, a 405 nm light was used to excite the isosbestic signal to correct motion artifacts and drift in fluorescence intensity. To evaluate responses of VP ChAT neurons and ACh release to mechanical (2 g von Frey filament) stimuli, and locomotion, we designated 3 s GCaMP6, eYFP, or GACh signal just prior to the stimulation or movement as the baseline value (*F*_0_), whose mean and standard deviation (SD) were calculated. These values were used to transform each point in the GCaMP6, GACh, and eYFP traces into the z-score ((*F* − Mean)/SD). We then measured the peak z-score and AUC of the z-score plot to quantify the response to mechanical stimulation or alterations upon paw-lifting at the beginning of voluntary movement. In each mouse, data from 3 to 5 trials of mechanical paw withdrawal or paw lifting when locomotion was initiated were collected for analysis. We included peak z-scores or/and peak AUC of all trials for statistical analysis.

### Optogenetic manipulation

For optogenetic manipulations [23,43], 472 nm laser generators (Inper, Hangzhou, China) were used to deliver light to activate ChR2 transfected into the STN-VP projection and VP cholinergic neurons with stimulation parameters indicated in the figure legends or the Results section.

### Pharmacological modulation

Mice with cannula implants in the VP were anesthetized with 1% isoflurane and 0.2 μl saline with or without drugs (agonists and antagonists of nicotinic receptors, or clozapine-N-oxide (CNO)) was injected into the VP with a microinjector (KD Scientific, Holliston, MA, USA) at the speed of 100 nl/min [43]. After drug injection, the needle was remained for 5 min in injection site before it was removed. Behavioral tests for mice received injection of nicotinic drugs or CNO were performed 15 min or 45 min after the mice recovered from anesthesia, respectively.

In some circumstances, CNO or naloxone was intraperitoneally administered as indicated in the Results section.

### Establishment of pain-like mouse models

#### Inflammatory pain mouse model.

Capsaicin (0.01%, 20 µl in 10% DMSO/saline) (From MedChemExpress) was injected in the lower hind limb. Pain thresholds were measured 30 min later.

#### Neuropathic pain mouse model.

SNI was performed according to previous reports [23,40]. Mice were anesthetized with the inhalation of isoflurane (3% for induction, 1.5% for maintenance), the fur from the knee to the hip was shaved, and the skin was sterilized three times with 75% alcohol. After a longitudinal incision on the skin of the hip was made, the biceps femoris muscle was separated with a pair of blunt forceps to expose the sciatic nerve and its branches (sural, common peroneal, and tibial nerves). The common peroneal and tibial nerves were tightly ligated with a nylon suture and a section of the distal nerve bundle (2 mm) in each nerve was removed, but the sural nerve was kept intact. After the muscle and skin incisions were subsequently sutured, the mice were allowed to recover on a heating pad. Some mice that were not subjected to nerve ligation and severing were used as sham controls. Mechanical and thermal pain thresholds in the skin area innervated by the sural nerve were measured in SNI and sham mice.

### Pain threshold tests

Mechanical threshold was measured by using a series of *von Frey* filaments with fiber forces ranging from 0.008*g* to 4*g*, and calculated according to an up-down method [23,40,44]. In brief, the mice were acclimatized in a test chamber (8 × 8 × 8 cm^3^) with a bottom made of meshed wires. The first fiber (0.4*g*) was applied perpendicular to the hind paw until the fiber bent, and then held in place for 2 s. If this did not elicit a withdrawal response, the next fiber with a higher force was applied. This process continued until a withdrawal response was observed. If the mice responded, the fiber with a lower force was applied on the subsequent trial. A cut-off value of 3.0*g* in fiber force was assigned because this filament lifts the hind paw before it bends. The 50% PWT was calculated using Dixon’s up-down method [44].

A plantar anesthesia tester (Boerni, Tianjin, China) was used to measure thermal threshold on both hind paws, which were quantified with thermal PWLs [22,40,45]. In brief, the mice were acclimatized in a test compartment on a glass surface. A heat light source positioned over the plantar surface of the hind paw was turned on until the mouse withdrew its paw. The duration of the light stimulation was recorded as thermal PWL. To prevent potential tissue damage to the surface of the paw, a 20 s cut-off time was set in the light source.

### Open field test

Each mouse was placed in the center of a square open field arena (30 × 30, 20 cm in height) and allowed to explore freely for 6 min, while their activities were recorded from a video camera above the arena, which was controlled by Ethovision XT 14.0 software (Noldus Information Technology, Wageningen, the Netherlands) [43]. The total distance and moving velocity were analyzed to evaluate locomotor activity. Time in the center zone (15 × 15 cm central area) of the arena in the first 6 min was analyzed to evaluate anxiety level of the mice.

For the examination of motor control by the STN-VP projection, the mice were placed in an open field arena, allowed for 10 min free roaming, then received 3 min bilateral or unilateral optogenetic stimulation of the STN-VP projection, and recovered for 7 min. The data were analyzed with EthoVision XT 14.0 software online or offline. The effect of optogenetic stimulation on motor function was evaluated by comparing total distance per min within 5 min before optogenetic stimulation and 3 min during optogenetic stimulation. We also compared time in the center zone of the arena 3 min before and 3 min during optogenetic stimulation to evaluate the changes in anxiety-like behavior.

### Elevated plus maze

EPM is a method to test anxiety levels [46,47]. A Plexiglas maze in form of a plus was elevated 50 cm above the ground. The maze consists of a central platform (5 × 5 cm) and four arms: two open arms (30 × 5 cm) and two closed arms (30 × 5, 20 cm tall walls) with open roofs. Same as the two closed arms, the two open arms are opposite each other and converge into the central platform. A video camera was mounted above the maze to record mouse behaviors for 6 min. The data were analyzed with EthoVision XT 14.0 software online or offline.

### Tail suspension test

TST is a behavioral task used for assessment of depression-like state [48]. Each mouse was hung from a bar about 40 cm above the ground by its tail using an adhesive tape that was placed approximately 1 cm away from the tip of the tail. Each mouse was tested only once for 6 min. The mice’s behavior was recorded with a video camera from the side, and the duration of immobility was measured in the last 5 min.

### Forced swim test

FST is a method to assess depression-like state [49]. Each mouse was placed in a glass cylinder (30 cm height and 20 cm diameter) that filled with water (25 ± 1 °C). The water level was 15 cm from the bottom. Each mouse was tested only once for 6 min. The animal’s behavior was recorded for 5 min with a video camera from the side, and the duration of immobility of each mouse during the last 4 min of the test was analyzed.

### Brain-slice patch-clamp recordings

A vibratome (VT-1200S, Leica Microsystems, Nussloch, Germany) was used to prepare parasagittal slices (300 µm) in an ice-cold modified sucrose-based artificial cerebral spinal fluid (sACSF) [22,40,50,51], saturated with 95% O_2_/ 5% CO_2_ (carbogen). The sACSF contains (in mM) 85 NaCl, 75 sucrose, 2.5 KCl, 1.25 NaH_2_PO_4_, 4.0 MgCl_2_, 0.5 CaCl_2_, 24 NaHCO_3_, and 25 glucose. Slices containing the VP or the STN were incubated in sACSF at 32°C for at least 75 min, then transferred to and incubated in carbogenated normal ACSF (26°C, containing (in mM) 125 NaCl, 2.5 KCl, 1.25 NaH_2_PO_4_, 1.2 MgCl_2_, 2.4 CaCl_2_, 26 NaHCO_3_, and 11 glucose. During experiments, brain slices were placed in a recording chamber, whose temperature was kept at 32 ± 0.5 °C with a thermostat (Warner Instrument, Hamden, CT, USA), and were perfused with ACSF at a speed of 1.5 ml/min.

Neurons in brain slices (3−5 slices from each mouse) were visualized and recorded in a patch-clamp recording rig, consisting of an upright microscope (FN-1, Nikon) equipped with a CCD-camera (Flash 4.0 LTE, Hamamatsu), a MultiClamp 700B amplifier, a Digidata 1550B analog-to-digital converter, and a pClamp 10.7 software (Molecular Devices, San Jose, CA). The patch electrodes used for recording had a tip with a diameter between 1 and 1.5 μm. The electrode had a resistance of 4‒6 MΩ when filled with a low-chloride intrapipette solution containing (in mM) 135 K gluconate, 5 KCl, 0.2 EGTA, 0.5 CaCl_2_, 10 HEPES, 2 Mg-ATP, and 0.1 GTP [22,50]. The pH value of the intrapipette solution was adjusted to 7.2 with Tris-base and its osmolarity was adjusted to 300 mOsm with sucrose.

To confirm the function of ChR2-eYFP transfected into STN neurons, STN and VP neurons were recorded with the whole-cell patch-clamp technique, and blue light (460 nm, 5 ms, 2.0 mW, to activate ChR2) was delivered through an optical fiber (200 µm in diameter, NA 0.37, 200 μm away from the recorded neuron) connected to a PlexBright LED light source (Plexon Inc, Hongkong, China). To characterize pharmacological properties of inward currents in VP cholinergic neurons evoked by optogenetic stimulation of the STN-VP projection, 20 μM CNQX or 0.5 μM TTX + 0.1 mM 4-aminopyridine (4-AP) was bath applied for 5–10 min.

To record currents mediated by nAChRs, acetylcholine (0.3 mM), RJR-2403 (50 μM), PNU282987 (30 μM) or varenicline (100 μM) in ACSF was individually filled in a patch pipette, which as placed 20 μm away from the voltage-clamped neuron, and a 0.5 s air pressure (6 pSi) was applied to puff the solution onto the soma of a VP neuron with a Pico-liter injector (Warner Instrument, Hamden, CT, USA). To evaluate the pharmacological properties of ACh-induced inward currents, 10 μM MEC, 0.5 μM TTX, or 20 μM CNQX + 10 μM GABAzine, was bath-applied for 5–10 min.

### Histology

Mice were sacrificed in a CO_2_ chamber and then subjected to cardiac perfusion with 10 ml phosphate-buffered saline (PBS), followed by 10 ml 4% paraformaldehyde (PFA) in PBS. Mouse brains were removed, post-fixed in 4% PFA overnight at 4°C for 12–16 h, and dehydrated in 30% of sucrose until sank for cryoprotection. After trimmed and marked the left hemisphere, the brain samples were imbedded in OCT, frozen below −20 °C and cut into 30 μm coronal sections with a CM1950 cryostat (Leica, Nussloch, Germany) at −17 °C. The sections were mounted onto glass slides. For immunostaining [22,52], brain sections were incubated in a blocking buffer containing 5% donkey serum and 0.1% Triton X-100 for 90 min at room temperature. Then the sections were incubated with primary antibody diluted in blocking buffer for 48 h at 4°C.

Primary antibodies include (1) rabbit anti-c-Fos IgG, 1:2000, c-Fos (9F6) Rabbit mAb, Cell Signaling Technology; (2) Goat anti-ChAT IgG, 1:200, Millipore; (3) rat anti-SP IgG, 1:200, Sigma-Aldrich; (4) Mouse anti-CaMKII IgG, 1:300, Cell signaling Technology; (5) Rabbit anti-GABA, 1:250, Sigma-Aldrich.

After washing three times (10 min each) with PBS, the sections were incubated with secondary antibodies (Jackson ImmunoResearch, West Grove, PA, USA) for 90–120 min at room temperature.

Secondary antibodies include (1) Donkey anti-rabbit Alexa 488, 1:500; (2) Donkey anti-rabbit Cy3, 1:500; (3) Donkey anti-rabbit Alexa 647, 1:500; (4) Donkey anti-goat Alexa 647, 1:500; (5) Donkey anti-rat Alexa 647, 1:500; (6) Donkey anti-mouse Alexa 647, 1:500.

The sections were washed three times (10 min each) with PBS, dried in the dark, cover-slipped in mounting medium, and then were imaged with a confocal microscope (Fv-1000, Olympus, Tokyo, Japan). The images were processed with ImageJ (NIH, Bethesda, MD, USA).

### Quantitative real-time polymerase chain reaction

#### RNA extraction.

ChAT-Cre, Vgat-Cre and Vglut2-Cre mice subjected to AAV-EF1α-DIO-eYFP injection into the VP were euthanized in a CO_2_ chamber and decapitated. The brains were removed, washed with ice-cold artificial cerebrospinal fluid (ACSF), trimmed off the olfactory bulb and brain stem, frozen in liquid nitrogen, and then stored at −80 °C. The brain blocks were imbedded into the OCT and sectioned into 15 μm thick slices using a Cryostat (Leica CM 1950, Nussloch, Germany), and the slices were mounted onto pretreated nuclease-free glass slides. A laser capture microdissection system (Zeiss, Wetzlar, Germany) was used to collect eYFP-labeled VP cholinergic, GABAergic, or glutamatergic neurons into caps of 200 μl nuclease-free Eppendorf tubes filled with 5 μl lysis buffer (30 neurons/tube), and the tubes were stored at −80 °C for further assays.

#### cDNA synthesis.

Single Cell Sequence Specific Amplification Kit was used to reversely transcribe mRNA samples into cDNA according to the manufacturer’s manual (VazymE, China). To improve detection sensitivity, target genes were pre-amplified using a multiplex PCR pre-amplification protocol. The reaction program was as follows: reverse transcription at 50 °C for 60 min; initial denaturation at 95 °C for 3 min; followed by 20 cycles of denaturation at 95 °C for 15 s and annealing/extension at 60 °C for 15 min in a PCR machine (GE-4852T, BIO-GENER, Hangzhou, China). Following amplification, 20 μl of nuclease-free water was added to each reaction tube to achieve a 1:5 dilution. The resulting cDNA samples were kept at 4 or −20 °C for further analysis.

#### Real-time PCR.

A 20 μl reaction system was made with the mixture of cDNA samples, SYBR Green qPCR Master Mix (Low ROX), primers (S1 Table), and ddH_2_O in a 200 μl Eppendorf tube. An ABI QuatStudio7 Flex (LifeTechnologies, Carlsbad, CA) was used to amplify the cDNA samples with an optimized protocol (95 °C for 15 s, 60 °C for 20 s, and then 72 °C for 30 s) for 40 cycles. A melt curve was obtained by heating the sample at 95 °C for 15 s, at 60 °C for 60 s, and then at 95 °C for 15 s. Each sample was amplified in two duplicated tubes. The level of a particular gene was normalized to that of *Gapdh*.

### Chemicals

Drugs, purchased from MedChemExpress (Monmouth Junction, NJ, USA), include 4-aminopyridine (4-AP), acetylcholine chloride, clozapine-N-oxide (CNO), dihydro-β-erythroidine hyrobromide (DHβE), mecamylamine hydrochloride (MEC), methyllycaconitine citrate (MLA), naloxone hydrochloride, PNU-282987, Rivanicline hemioxalate (RJR-2403), tetrodotoxin (TTX), and varenicline hydrochloride.

### Statistics and reproducibility

Data analysis and presentation complied with a previously published guideline [53].

For each experiment, we did an initial test with a sample size of 5, calculated the variation (standard deviation) in the parameters, and used the power analysis function in SigmaPlot 14.0 software (*α* = 0.05, *β* = 0.85) to estimate the sample sizes needed to obtain reliable statistics. Sample sizes are all greater than these values. In experiments with positive results, SigmaPlot 14.0 gives P values and power values at alpha = 0.05. Power value > 0.80 means that it is likely to detect the actual difference. In experiments with negative results, we made sure that the sample sizes were close to those in experiments with positive results.

For optogenetic and behavioral experiments, mice were randomly assigned to control/sham and experimental groups. All summarized data are expressed as the mean ± standard error of the mean (SEM). The error bars in histogram, time courses, and input-output curves are SEM. Two-tailed paired or unpaired *t*-tests, two-way ANOVAs, one-way ANOVAs followed by post-hoc Bonferroni tests, and one-way repeated-measures ANOVAs with post-hoc Bonferroni tests and Geisser-Greenhouse correction were used to compare the behavioral, electrophysiological, and histological data, as indicated in the figure legends. If equal-variance and normal distribution assumptions were not valid, statistical significance was evaluated by the Mann-Whitney rank sum test or by two-way or one-way ANOVA rank tests. Source data in all figures are provided in an excel data file named S1 Data submitted with this paper. In all tests, a value of two-tailed *P* < 0.05 was considered statistically significant. GraphPad Prism (version 8.0) was used for all statistical analyses.

After we finished each experiment, we sacrificed every mouse for histological assay to confirm the expression of viral vectors and localization of cannula for drug delivery and optical fiber for optogenetic modulation and fiber photometry recording. The images in Figures are representative ones among all from mice we collected data in individual experiments.

## Supporting information

S1 Fig Profile of upstream nuclei of VP cholinergic neurons. **(A)** Schematic diagram and timeline of retrograde tracing of upstream neurons innervating VP cholinergic neurons with AAV2/9-EF1α-DIO-TVA-GFP, AAV2/9- EF1α-DIO-oRVG, and RV-EnvA-tdTomoato. **(B)** Representative images showing GFP(+) neurons (green), ChAT(+) (purple), and tdTomato(+) (red) neurons in the VP. **(C)** Representative images showing nuclei containing tdTomato(+) neurons (red) in hemispheres ipsilateral to the starter VP cholinergic neurons. Blue, DAPI. **(D, E)** Summary showing % total inputs and density of tdTomato(+) neurons in hemispheres ipsilateral to the starter VP cholinergic neurons. *n* = 5 from 5 mice. Data are available in S1 Data as a part of Supporting information. Acb: nucleus accumbens; ACC: anterior cinglulate cortex; BLA: basolateral amygdala; BLP: basolateral amygdala posterior part; CeA: central amygdala; DR: dorsal raphe nucleus; LS: lateral septum; PBN: parabrachial nucleus; PF: parafascicular nucleus of the thalamus; PIC: posterior insular cortex; PPTg: pedunculopontine tegmental nucleus; PVA: paraventricular area of the hypothalamus; Re: reunion nucleus of the thalamus; PV/MD: Paraventricular/mediodorsal area in the thalamus; SNc: substantia nigra pars compacta; VP: ventral pallidum; VTA: ventral tegmental area; mPFC: medial prefrontal cortex.
(TIF)

S2 Fig STN glutamatergic neurons send monosynaptic projections onto VP cholinergic neurons. **(A)** Schematic diagram for virus injection to fulfill optogenetic stimulation of the STN-VP glutamatergic projection (AAV2/9-CaMKII-ChR2-eYFP in the STN) and cell-specific fluorescence labeling of cholinergic neurons (AAV2/9-EF1α-DIO-mCherry in the VP) in ChAT-Cre mice. **(B)** Representative images showing transfection of CaMKII-ChR2-eYFP and mCherry into the STN and VP. **(C)** Diagram for patch-clamp recording of VP cholinergic neurons upon optogenetic stimulation of the STN-VP glutamatergic projection. **(D)** Representative traces showing blue light-evoked inward currents sensitive to CNQX (20 μM) and APV (50 μM). **(E)** Summary of amplitude and paired-pulse (with 50 ms interval) ratio of photo-EPSCs in VP cholinergic neurons (*n* = 22–25). **(F)** Representative traces and summary of postsynaptic currents evoked by paired blue light pulses (50 ms apart) in VP cholinergic neurons before and during incubation of 1 μM tetrodotoxin (TTX) and 0.3 mW 4-aminopyridine (4-AP). *n* = 8. **(G, H)** Representative traces of postsynaptic currents evoked by blue light pulses (5, 10, 20, and 50 Hz) and success rate for light-evoked currents, *n* = 10. Data from 3 mice. Data are available in S1 Data as a part of Supporting information.
(TIF)

S3 Fig Unilateral stimulation of the STN-VP projection does not affect locomotion and emotion in mice. **(A)** Schematic diagram for injecting AAV-CaMKII-ChR2-eYFP (ChR2) or AAV-CaMKII-eYFP (eYFP) and implanting optical fiber into the STN in ChAT-Cre mice. **(B)** Representative images showing eYFP-labeled STN neurons and their projections to the VP, and optical fiber track targeting the VP. **(C, D)** Total distance and velocity in the open field tests in ChR2 (*n* = 9) and eYFP (*n* = 9) mice before, during, and after bilateral blue light illumination of the VP. **(C)** Distance. ChR2: *F*_(1.371, 12.34)_ = 9.69, *P* = 0.005; *t* = 3.37, *P* = 0.02, control vs. light. eYFP: *F*_(1.901, 11.41)_ = 5.22, *P* = 0.03. *t* = 2.82, *P* = 0.09, control vs. light. **(D)** Velocity. ChR2: *F*_(1.901, 11.41)_ = 5.22, *P* = 0.006; *t* = 3.35, *P* = 0.03, control vs. light. eYFP: *F*_(1.916, 11.50)_ = 5.62, *P* = 0.02. *t* = 2.89, *P* = 0.08, control vs. light. **(E)** Time in the center zone in the open field test in ChR2 and eYFP mice before, during, and after bilateral blue light illumination of the VP. ChR2: *F*_(1.656, 14.90)_ = 1.82, *P* = 0.20. eYFP: *F*_(1.123, 8.987)_ = 2.83, *P* = 0.12. **(F, G)** Total distance and time in the center zone in the open field tests in ChR2 (*n* = 9) and eYFP (*n* = 9) mice before, during, and after unilateral blue light illumination of the VP. **(F)** Total distance. ChR2: *F*_(1.232, 11.09)_ = 0.08, *P* = 0.83. eYFP: *F*_(1.806, 14.45)_ = 1.06, *P* = 0.36. **(G)** Time in the central zone. ChR2: *F*_(1.806, 16.26)_ = 2.67, *P* = 0.10. eYFP: *F*_(1.297, 10.37)_ = 1.06, *P* = 0.35. **(H)** % of contralateral rotation in the open field tests in ChR2 (*n* = 9) and eYFP (*n* = 9) mice before, during, and after unilateral blue light illumination of the VP. * *P* < 0.05. ns not significant. One-way repeated measures ANOVAs for **(C-H)**. Data are available in S1 Data as a part of Supporting information.
(TIF)

S4 Fig Responses of VP cholinergic neurons to mechanical stimulation on hind paws with and without chemogenetic inhibition of the STN-VP projection. This figure presents decay time and area under the curve (AUC) of responses in VP cholinergic neurons to mechanical stimulation on hind paws using the same datasets for Fig 2F–2O. **(A, B)** Decay time and area under the curve of responses of of VP cholinergic neurons to mechanical stimulation on the contralateral hind paw before (saline) and during (CNO) chemogenetic inhibition of the STN-VP projection. **(A)** Decay time. *F*_(3, 76)_ = 21.08, *P* < 0.001. Saline: *t* = 4.74, *P* < 0.001, Naive vs. SNI. CNO: *t* = 6.3, *P* < 0.001, Naive vs. SNI. *n* = 20. **(B)** AUC. *F*_(4, 95)_ = 24.86, *P* < 0.001. Saline: *t* = 4.83, *P* < 0.001, Naive vs. SNI. CNO: *t* = 2.39, *P* = 0.01, Naive vs. SNI. *n* = 20. **(C, D)** Decay time and area under the curve of responses of of VP cholinergic neurons to mechanical stimulation on the ipsilateral hind paw before (saline) and during (CNO) chemogenetic inhibition of the STN-VP projection. **(C)** Decay time. *F*_(3, 76)_ = 19.45, *P* < 0.001. Saline: *t* = 2.93, *P* = 0.005, Naive vs. SNI. CNO: *t* = 6.99, *P* < 0.001, Naive vs. SNI. *n* = 20. **(D)** AUC. *F*_(4, 95)_ = 28.82, *P* < 0.001. Saline: *t* = 3.28, *P* = 0.003, Naive vs. SNI. CNO: *t* = 2.78, *P* = 0.006, Naive vs. SNI. *n* = 20. ** *P* < 0.01. One-way ANOVAs for **(A–D)**. Data are available in S1 Data as a part of Supporting information.
(TIF)

S5 Fig VP-projecting STN neurons controls mechanical responses in VP cholinergic neurons in mice. **(A, B)** Schematic diagram and representative images for injecting AAV-EF1α-DIO-GCaMP6s or AAV-EF1α-DIO-eYFP and implanting optical fiber into the VP in ChAT-Cre mice. Meanwhile, AAV retro-hSyn-Flp was injected into the VP, and AAV-fDIO-hM4Di-mCherry was injected into the STN to transfect hM4Di into VP-projecting STN neurons. **(C**–**H)** Heat maps, averaged traces, and summary showing changes in GCaMP6s (*n* = 5 mice) and eYFP (*n* = 5 mice) in response to suprathreshold von Frey filament stimulation onto the contralateral hind paw of naïve and SNI mice before and after intraperitoneal injection of CNO (3 mg/kg). **(G)** Contralateral stimulation evoked peak z-score. Naive, *F*_(2, 42)_ = 32.37, *P* < 0.001; *t* = 5.41, *P* < 0.001, Saline vs. CNO. SNI, *F*_(2, 42)_ = 44.76, *P* < 0.001; *t* = 6.82, *P* < 0.001, Saline vs. CNO. **(H)** Contralateral stimulation evoked peak AUC. Naive, *F*_(2, 42)_ = 22.47, *P* < 0.001; *t* = 4.51, *P* < 0.001, Saline vs. CNO. SNI, *F*_(2, 42)_ = 31.74, *P* < 0.001; *t* = 5.34, *P* < 0.001, Saline vs. CNO. **(I**–**N)** Heat maps, averaged traces, and summary showing changes in GCaMP6s and eYFP in response to suprathreshold von Frey filament stimulation on the ipsilateral hind paw of naïve and SNI mice before and after intraperitoneal injection of CNO (3 mg/kg). **(M)** Ipsilateral stimulation evoked peak z-score. Naive, *F*_(2, 42)_ = 84.62, *P* < 0.001; *t* = 7.79, *P* < 0.001, Saline vs. CNO. SNI, *F*_(2, 42)_ = 67.72, *P* < 0.001; *t* = 7.60, *P* < 0.001, Saline vs. CNO. **(N)** Ipsilateral stimulation evoked peak AUC. Naïve, *F*_(2, 41)_ = 25.11, *P* < 0.001; *t* = 5.06, *P* < 0.001, Saline vs. CNO. SNI, *F*_(2, 42)_ = 34.95, *P* < 0.001; *t* = 5.73, *P* < 0.001, Saline vs. CNO. **(O**–**R)** Heat maps, representative traces, averaged traces, and summary showing changes of GCaMP6 signal during episodes of voluntary movement before and after intraperitoneal administration of CNO (3 mg/kg). **(Q)** Peak. *t* = 3.57, *P* = 0.002, GCaMP6 vs. eYFP. **(P)** AUC. *t* = 2.86, *P* = 0.008, GCaMP6 vs. eYFP. ** *P* < 0.01; ns not significant. One-way ANOVA for **(G, H, M, N)**. Two-tailed *t* test for **(Q, R)**. Data are available in S1 Data as a part of Supporting information.
(TIF)

S6 Fig The STN-VP projection onto VP cholinergic neurons is enhanced in SNI mice. **(A)** Schematic diagram for virus injection to fulfill optogenetic stimulation of the STN-VP glutamatergic projection (AAV2/9-CaMKII-ChR2-eYFP in the STN) and cell-specific fluorescence labeling of cholinergic neurons (AAV2/9-EF1α-DIO-mCherry in the VP) in ChAT-Cre mice. **(C, D)** Representative traces and summary of amplitude of 5 ms blue light-evoked currents in VP cholinergic neurons in Sham and SNI mice. *n* = 25 neurons from 3 mice in each group. *t* = 6.99, *P* < 0.0001, SNI vs. sham. **(E, F)** Representative traces and summary of postsynaptic currents evoked by paired blue light pulses (50 ms apart) on VP cholinergic neurons from sham and SNI mice. *n* = 25 cholinergic neurons from each group, *t* = 3.09, *P* = 0.003, Sham vs. SNI. ** *P* < 0.01. Two-tailed *t* test for **(D, E)**. Data are available in S1 Data as a part of Supporting information.
(TIF)

S7 Fig Pain stimulation induces ACh release in the VP in sham and SNI mice. **(A**–**C)** Heat maps, averaged fluorescent intensity of GACh and eYFP in the VP during episode of voluntary movement. Sixteen trials from 8 GACh mice and 5 eYFP mice. Peak. *t* = 2.47, *P* = 0.02. AUC. *t* = 2.41, *P* = 0.02, GACh vs. eYFP. Twenty trials from 5 mice in each group. Two-tailed *t*-tests for **(C)**. **(D**–**G)** Schematic diagram **(D**) for viral injection strategy and representative images showing transfection of GACh into the VP **(E)** and hM4Di-mCherry **(F)** into VP cholinergic neurons in ChAT-Cre mice. **(G)** ChAT neurons accounted for 93.9% of hM4Di-mCherry neurons. 77.78% of ChAT neurons were labeled with hM4Di-mCherry. Data from 3 slices (1 from each mouse) were summarized. **(H–L)** Heat maps, averaged traces, and summary showing changes in GACh (*n* = 6 mice) and eYFP (*n* = 5 mice) in response to suprathreshold von Frey filament stimulation on the contralateral hind paw of naïve and SNI mice before and after intraperitoneal injection of CNO (3 mg/kg). Naïve: *F*_(2, 39)_ = 31.35, *P* < 0.001; *t* = 6.19, *P* < 0.001, saline vs. CNO. SNI: *F*_(2, 39)_ = 42.30, *P* < 0.001; *t* = 6.49, *P* < 0.001, saline vs. CNO. **(M**–**Q)** Heat maps, averaged traces, and summary showing changes in GACh (*n* = 6) and eYFP (*n* = 5) in response to suprathreshold von Frey filament stimulation on the ipsilateral hind paw of naïve and SNI mice before and after intraperitoneal injection of CNO (3 mg/kg). Naïve: *F*_(2, 39)_ = 23.54, *P* < 0.001; *t* = 4.10, *P* < 0.001, saline vs. CNO. SNI: *F*_(2, 39)_ = 51.58, *P* < 0.001; *t* = 7.77, *P* < 0.001, saline vs. CNO. **(R**–**S)** Peak AUC of contralateral VP, ipsilateral VP in Naive and SNI and eYFP mice, before and after intraperitoneal administration of CNO (3 mg/kg). **(R)** Naive: *F*_(2, 39)_ = 23.13, *P* < 0.001.; *t* = 4.76, *P* < 0.001, saline vs. CNO. SNI: *F*_(2, 39)_ = 12.94, *P* < 0.001; *t* = 3.88, *P* < 0.001, saline vs. CNO. **(S)** Naive: *F*_(2, 39)_ = 18.84, *P* < 0.001; *t* = 4.97, *P* < 0.001, saline vs. CNO. SNI: *F*_(2, 39)_ = 17.67, *P* < 0.001; *t* = 4.41, *P* < 0.001, saline vs. CNO. * *P* < 0.05; ** *P* < 0.01; ns not significant. One-way ANOVAs for **(L, Q, R, S)**. Data are available in S1 Data as a part of Supporting information.
(TIF)

S8 Fig ACh-evoked currents in VP neurons do not depend on presynaptic neurotransmitter release. **(A)** Schematic diagram for labeling of VP neuron types with viral vector and Cre-mouse lines. **(B)** Representative images showing eYFP-labeled cholinergic, glutamatergic, and GABAergic neurons in the VP. **(C, D)** Schematic diagram and representative fluorescent and bright-field images showing collection of eYFP-labeled neurons in the VP under a laser capture microscope. **(E)** qRT-PCR was performed to analyze the mRNA of *ChAT*, *Vglut2*, and *Vgat* genes in eYFP-labeled ChAT, Vglut2, and Vgat neurons. ChAT neurons: *F*_(2, 21)_ = 7.21, *P* = 0.004; *t* = 3.21, *P* = 0.008, ChAT vs. Vglut2; *t* = 3.36, *P* = 0.006, ChAT vs. Vgat). *n* = 8 samples. Vglut2 neurons: *F*_(2, 21)_ = 24.58, *P* < 0.001; *t* = 6.15, *P* < 0.001, ChAT vs. Vglut2; *t* = 5.98, *P* < 0.001, Vglut2 vs. Vgat. *n* = 8 samples. Vgat neurons: Kruskal-Wallis *U* = 20.48, *P* < 0.001; *t* = 2.26, *P* = 0.047, ChAT vs. Vgat; *t* = 4.52, *P* < 0.001, Vglut2 vs. Vgat. *n* = 8 samples from 3 mouse for each type of neurons. **(F)** Images of gel electrophoresis results of qRT-PCR products from eYFP-labeled neurons in ChAT-Cre, Vglut2-Cre, and Vgat-Cre mice. **(G, H)** Representative traces and summary of amplitudes of 0.3 mM ACh-evoked inward currents in the absence and presence of 0.5 μM tetrodotoxin (TTX) or 20 μM + 10 μM GABAzine in VP ChAT neurons. *F*_(1.812, 19.93)_ = 0.54, *P* = 0.58. *n* = 10. **(I, J)** Representative traces and summary of amplitudes of 0.3 mM ACh-evoked inward currents in the absence and presence of 0.5 μM TTX or 20 μM + 10 μM GABAzine in VP Vgat neurons. *F*_(1.812, 19.93)_ = 0.54, *P* = 0.58. *n* = 10. **(K, L)** Representative traces and summary of amplitudes of 0.3 mM ACh-evoked inward currents in the absence and presence of 0.5 μM TTX or 20 μM + 10 μM GABAzine in VP Vglut2 neurons. *F*_(1.293, 14.22)_ = 1.12, *P* = 0.33. *n* = 10. * *P* < 0.05, ** *P* < 0.01, ns not significant. One-way ANOVAs with Bonferroni tests for **(E)**. One-way repeated measures ANOVAs with Bonferroni tests for **(H, J, L)**. Data are available in S1 Data as a part of Supporting information. Images in **(F)** were cropped from S1_raw_image.pdf submitted as supporting information.
(TIF)

S9 Fig Spontaneous and evoked firing in VP neurons are differentially modulated by blockers of nAChRs, glutamate receptors, and GABA_A_ receptors. **(A, B)** Representative traces and summary of spontaneous firing in VP cholinergic neurons from sham and SNI mice (*n* = 15 neurons from 3 mice in each group) in the absence and presence of CNQX+GABAzine or MEC. Sham: *F*_(1.798, 25.17)_ = 2.77, *P* = 0.08; *t* = 2.77, *P* = 0.04, CNQX+GABAzine vs. MEC. SNI: *F*_(1.393, 19.51)_ = 12.93, *P* = 0.0008; *t* = 2.11, *P* = 0.05, baseline vs. CNQX+GABAzine; *t* = 4.42, *P* = 0.0014, CNQX+GABAzine vs. MEC; *t* = 4.51, *P* = 0.0014, baseline vs. MEC. **(C, D)** Representative traces and summary of firing evoked by depolarizing current injections in VP ChAT neurons from sham and SNI mice. Currents, *F*_(2.083, 175.0)_ = 491.4, *P* < 0.001; Group, *F*_(5, 84)_ = 3.34, *P* = 0.008; Interaction, *F*_(50, 840)_ = 2.42, *P* < 0.001. Data from 15 neurons from 3 mice in each group. **(E)** 160 pA current-evoked firing. Drugs, *F*_(1, 28)_ = 6.30, *P* = 0.018; Group, *F*_(2, 56)_ = 1.87, *P* = 0.16; Interaction, *F*_(2, 56)_ = 1.10, *P* = 0.33. Baseline, *t* = 2.89, *P* = 0.005, Sham vs. SNI. MEC, *t* = 2.03, *P* = 0.045, sham vs. SNI. *n* = 15 neurons from 3 mice in each group. **(F, G)** Representative traces and summary of spontaneous firing in VP GABAergic neurons from sham and SNI mice (*n* = 15 neurons from 3 mice in each group) in the absence and presence of CNQX+GABAzine or MEC. Sham: *F*_(1.421, 19.90)_ = 0.59, *P* = 0.50. SNI: *F*_(1.959, 27.43)_ = 8.49, *P* = 0.0014; *t* = 4.41, *P* = 0.0018, baseline vs. CNQX+GABAzine; *t* = 2.32, *P* = 0.07, CNQX+GABAzine vs. MEC; *t* = 1.67, *P* = 0.11, baseline vs. MEC. *n* = 15 neurons from 3 mice in each group. **(H, I)** Representative traces and summary of firing evoked by depolarizing current injections in VP GABA neurons from sham (*n* = 15) and SNI (*n* = 14) mice (3 in each group). Currents, *F*_(2.327, 188.5)_ = 274.6, *P* < 0.001; Group, *F*_(5, 81)_ = 5.53, *P* = 0.0002; Interaction, *F*_(50, 810)_ = 1.26, *P* = 0.011. **(J)** 160 pA current-evoked firing. Drugs, *F*_(1, 27)_ = 8.43, *P* = 0.007; Group, *F*_(2, 54)_ = 3.55, *P* = 0.035; Interaction, *F*_(2, 54)_ = 3.20, *P* = 0.048. MEC, *t* = 3.82, *P* = 0.0003, Sham vs. SNI. sham; *t* = 3.29, *P* = 0.005, Baseline vs. MEC. **(K, L)** Representative traces and summary of firing evoked by depolarizing current injections in VP glutamatergic neurons from sham (*n* = 17) and SNI (*n* = 16) mice (3 in each group). Sham, *F*_(1.338, 21.41)_ = 16.65, *P* < 0.001; *t* = 2.82, *P* = 0.01, baseline vs. CNQX+GABAzine; *t* = 4.41, *P* = 0.0016, CNQX+GABAzine vs. MEC; *t* = 5.21, *P* < 0.001, baseline vs. MEC. SNI, *F*_(1.537, 23.05)_ = 2.21, *P* = 0.14. **(M, N)** Representative traces and summary of firing evoked by depolarizing current injections in VP vglut2 neurons from sham (*n* = 16 neurons) and SNI (*n* = 16 neurons) mice (3 in each group). Currents, *F*_(2.447, 220.3)_ = 309.1, *P* < 0.001; Group, *F*_(5, 90)_ = 2.49, *P* = 0.04; Interaction, *F*_(50, 900)_ = 0.88, *P* = 0.70. **(O)** 160 pA current-evoked firing. Drugs, *F*_(1, 15)_ = 1.105; Group, *F*_(2, 30)_ = 4.92, *P* = 0.01; Interaction, *P* = 0.31. *t* = 2.65, *P* = 0.012, Sham vs. SNI. *t* = 3.21, *P* = 0.009, sham-Baseline vs. sham-MEC. * *P* < 0.05, ** *P* < 0.01, ns not significant. One-way repeated measures ANOVAs with Bonferroni tests for **(B, E, G, J, L, O)**. Two-way repeated measures ANOVAs with Bonferroni tests for **(D, I, N)**. CNQX-GABA: CNQX + GABAzine. CN-GA: CNQX + GABAZine. Data are available in S1 Data as a part of Supporting information.
(TIF)

S10 Fig Acetylcholine receptor subunits on VP neurons are differentially modified in neuropathic pain. qRT-PCR was performed to analyze the acetylcholine receptor mRNAs in eYFP-labeled ChAT, glutamatergic and GABAergic neurons. The levels of nAChR and mAChR subunit genes were normalized to that of *Gapdh*. **(A, B)** Schematic diagram **(A)** and representative images **(B)** for specific labeling of cholinergic, glutamatergic, and GABAergic neurons in the VP with viral vector (AAV-EF1α-DIO-eYFP) in ChAT-Cre, Vglut2-Cre, and Vgat-Cre mice. **(C, D)** PWT and PWL on hind paws of mice (*n* = 8) before and after injection of DHβE into the VP. **(C)** PWT. *F*_(2.600, 18.20)_ = 0.23, *P* = 0.85. Contralateral: *t* = 0.32, *P* = 0.76; Ipsilateral: *t* = 0.70, *P* = 0.76. **(D)** PWL. *F*_(2.144, 15.01)_ = 2.19, *P* = 0.14. Contralateral: *t* = 0.35, *P* = 0.73; Ipsilateral: *t* = 0.91, *P* = 0.63. **(E, F)** PWT and PWL on hind paws of mice (*n* = 8) before and after injection of MLA into the VP. **(E)** PWT. *F*_(2.421, 16.95)_ = 0.39, *P* = 0.14. Contralateral: *t* = 1.11, *P* = 0.51; Ipsilateral: *t* = 0.21, *P* = 0.84. **(F)** PWL. *F*_(2.226, 15.58)_ = 1.23, *P* = 0.32. Contralateral: *t* = 2.19, *P* = 0.13; Ipsilateral: *t* = 0.33, *P* = 0.75. **(G)** Comparison of mRNA levels of acetylcholine receptor subunit genes in ChAT neurons between sham and SNI mice. α2: *t* = 1.53, *P* = 0.15. α3: *t* = 1.49, *P* = 0.17. α5: *t* = 0.68, *P* = 0.50. α6: *t* = 1, *P* = 0.33. β2: *t* = 0.007, *P* = 0.99. β3: *t* = 2.94, *P* = 0.01. β4: *t* = 0.90, *P* = 0.38I. M1: *t* = 2.93, *P* = 0.01. M2: *t* = 1.27, *P* = 0.23. M3: *t* = 1.64, *P* = 0.12. M4: *t* = 3.55, *P* = 0.003. M5: *t* = 6.35, *P* < 0.001. n = 8 for each type of receptor subunits in sham and mice. **(H)** Comparison of mRNA levels of acetylcholine receptor subunit genes in glutamatergic neurons between sham and SNI mice. α2: *t* = 1.49, *P* = 0.17. α3: *t* = 0.38, *P* = 0.71. α5: *t* = 1.07, *P* = 0.30. α6: *t* = 0.95, *P* = 0.36. β2: *t* = 1.96, *P* = 0.07. β3: *t* = 2.13, *P* = 0.05. β4: *t* = 1.47, *P* = 0.16. M1: *t* = 0.32, *P* = 0.75. M2: *t* = 1.67, *P* = 0.12. M3: *t* = 1.67, *P* = 0.11. M4: *t* = 2.15, *P* = 0.04. M5: *t* = 0.94, *P* = 0.35. *n* = 7–8 for each type of receptor subunits in sham and mice. **(I)** Comparison of mRNA levels of acetylcholine receptor subunit genes in GABAergic neurons between sham and SNI mice. α2: *t* = 0.68, *P* = 0.51. α3: *t* = 1.91, *P* = 0.08. α5: *t* = 2.33, *P* = 0.03. α6: *t* = 1.60, *P* = 0.13. β2: *t* = 0.02, *P* = 0.98. β3: *t* = 3.28, *P* = 0.005. β4: *t* = 1.14, *P* = 0.27. M1: *t* = 2.24, *P* = 0.04. M2: *t* = 2.54, *P* = 0.02. M3: *t* = 0.77, *P* = 0.45. M4: SNI *t* = 0.39, *P* = 0.70. M5: *t* = 0.67, *P* = 0.51. *n* = 7–8 for each type of receptor subunits in sham and mice. * *P* < 0.05, ** *P* < 0.01, ns not significant. Two-way repeated measures ANOVAs with for **(C**–**F)**. Two-sided t-tests for **(G**–**I)**. Data are available in S1 Data as a part of Supporting information.
(TIF)

S11 Fig Chrna4-ShRNA downregulates α4 nAChR function in VP ChAT neurons. **(A, B)** Schematic diagram and representative image for injection of viral vector carrying *Chrna4* gene shRNA into the VP of Vgat-Cre mice and patch-clamp recording of puff of RJR-2403- or varenicline-evoked nAChR currents on GABAergic neurons. **(C, D)** Representative traces and summary showing downregulation of α4 nAChR-mediated currents in 10 neurons from 5 shRNA mice relative to those in 10 neurons from 5 scrRNA mice. RJR-2403: *t* = 5.62, *P* < 0.001. Varenicline: *t* = 5.71, *P* < 0.001. **(E**–**H)** Mice with downregulation of α4 nAChR in VP performed locomotion, anxiety, Tail suspension test and Forced swimming test. **(E)** Open field tests. Total Distance: *t* = 0.94, *P* = 0.36, scrRNA vs. shRNA. Center time: *t* = 0.17, *P* = 0.86, scrRNA vs. shRNA. Center distance: *t* = 0.5, *P* = 0.62, scrRNA vs. shRNA. **(F)** Elevated plus maze. Open Distance: *t* = 2.08, *P* = 0.05, scrRNA vs. shRNA. Time in open arms: *t* = 2.38, *P* = 0.028, scrRNA vs. shRNA. Entries in open arms: *t* = 3.15, *P* = 0.005, scrRNA vs. shRNA. **(G)** Immobility time in the tail suspension tests (TST). *t* = 0.28, *P* = 0.77, scrRNA vs. shRNA. **(H)** Immobility time in the forced swim tests (FST). *t* = 2.17, *P* = 0.05, scrRNA vs. shRNA. **(I)** Quantification of mRNA levels of nAChR α4 and α7 subunits on VP GABAergic neurons from shRNAα4 (*n* = 5) and scrRNA (*n* = 5) mice. *α4*: *t* = 3.74, *P* = 0.001, shRNA-*α4* (*n* = 8) vs. scrRNA (*n* = 8). *α7*: *t* = 0.41, *P* = 0.90, shRNA-*α4* (*n* = 8) vs. scrRNA (*n* = 8). * *P* < 0.05, ** *P* < 0.01; ns, not significant. Two-tailed *t*-tests for **(E**–**I)**. Two-way ANOVAs with Bonferroni tests for **(D)**. OFT: open field test. EPM: elevated plus maze. TST: tail suspension test. FST: forced swim test. Data are available in S1 Data as a part of Supporting information.
(TIF)

S12 Fig Down-regulation of α7 nAChR subunit in VP GABAergic neurons attenuates modulation of mechanical and thermal thresholds by α7 nAChRs. **(A)** Schematic diagram for injection of AAV-CMV-DIO-EGFP-shRNA(Chrna7) (shRNA) or AAV-CMV-DIO-eGFP-shRNA(scramble) (scrRNA) and implantation of a cannula into the right VP of Vgat-Cre mice. **(B, C)** Representative images and summary showing eGFP expression (green) in GABAergic neurons (red) 3 weeks after virus injection. GABA and shRNA co-expression neurons accounted for 88.35% of shRNA neurons. Summary data were from 6 slices of 3 mice. **(D)** The levels of α4 and α7 mRNA in VP GABAergic neurons in shRNAa7 and scrRNA mice were quantified with qRT-PCR assay. α4: *t* = 0.27, *P* = 0.78; α7: *t* = 2.68, *P* = 0.024, scrRNA (*n* = 8) vs. shRNA (*n* = 8). **(E, F)** Time courses of PWT and PWL on either hind paw in mice subjected to injection of AAV-CMV-DIO-shRNA-α7-eGFP (shRNAa7) or AAV-CMV-DIO-scramble RNA-eGFP (scrRNA) into the VP of Vgat-Cre mice. **(E)** PWT. Interaction, *F*_(21, 252)_ = 0.87, *P* = 0.63; Group, *F*_(3, 36)_ =1.61, *P* = 0.2; Time, *F*_(6.006, 216.2)_ = 2.98, *P* = 0.008. **(F)** PWL. Interaction, *F*_(21, 252)_ = 1.04, *P* = 0.41; Group, *F*_(3, 36)_ = 1.15, *P* = 0.34; Time, *F*_(4.426, 159.3)_ = 2.12, *P* = 0.073. **(G**–**J)** PWT and PWL on either hind paw before and after microinjection of an α7 nAChR agonist, PNU-282987, into the VP of shRNAa7 (*n* = 10) and scrRNA (*n* = 10) mice. **(G)** Contralateral PWT. scrRNA: *t* = 5.39, *P* < 0.001, saline vs. PNU. shRNAa7: *t* = 4.77, *P* < 0.001, saline vs. PNU. **(H)** Ipsilateral PWT. scrRNA: *t* = 2.57, *P* = 0.038, saline vs. PNU. shRNAa7: *t* = 1.27, *P* = 0.21, saline vs. PNU. **(I)** Contralateral PWL. scrRNA: *t* = 4.38, *P* < 0.001, saline vs. PNU. shRNAa7: *t* = 0.38, *P* = 0.7, saline vs. PNU. **(J)** Ipsilateral PWL. scrRNA: *t* = 4.41, *P* < 0.001, saline vs. PNU. shRNAa7: *t* = 2.38, *P* = 0.02, saline vs. PNU. **(K**–**N)** PWT and PWL in scrRNA (*n* = 10) and shRNAa7 (*n* = 10) mice before and after injection of MLA into the contralateral VP in sham or SNI mice. **(K)** PWT in sham mice. scrRNA: *F*_(1.606, 14.45)_ = 1.76, *P* = 0.21. shRNAa7: *F*_(1.868, 16.82)_ = 1.64, *P* = 0.22. **(L)** PWL in sham mice. scrRNA: *F*_(1.761, 15.85)_ = 0.68, *P* = 0.50. shRNAa7: *F*_(1.777, 15.99)_ = 2.13, *P* = 0.15. **(M)** PWT in SNI mice. scrRNA: *F*_(1.381, 12.43)_ = 222.0, *P* < 0.001; *t* = 15.44, *P* < 0.001, Baseline vs. SNI; *t* = 5.17, *P* < 0.001, SNI vs. SNI + MLA. shRNAa7: *F*_(1.010, 9.089)_ = 65.27, *P* < 0.001; *t* = 8.47, *P* < 0.001, Baseline vs. SNI; *t* = 4.08, *P* = 0.005, SNI vs. SNI + MLA. **(N)** PWL in SNI mice. scrRNA: *F*_(1.527, 13.74)_ = 39.77, *P* < 0.001; *t* = 8.86, *P* < 0.001, Baseline vs. SNI. *t* = 3.13, *P* = 0.01, SNI vs. SNI + MLA. shRNAa7: *F*_(1.257, 11.32)_ = 61.95, *P* < 0.001; *t* = 8.38, *P* < 0.001, Baseline vs. SNI; *t* = 0.02, *P* = 0.98, SNI vs. SNI + MLA. **(O**–**R)** Mice with downregulation of α7 nAChR in VP performed locomotion, anxiety, Tail suspension test and Forced swimming test. **(O)** Total distance: *t* = 1.9, *P* = 0.073, scrRNA vs. shRNAa7. Center time: *t* = 1.46, *P* = 0.16, scrRNA vs. shRNAa7. Center distance: *t* = 1.05, *P* = 0.31, scrRNA vs. shRNAa7. **(P)** Open distance: *t* = 0.9, *P* = 0.38, scrRNA vs. shRNAa7. Time in open arms: *t* = 2.54, *P* = 0.03, scrRNA vs. shRNAa7. Entries in open arms: *t* = 1.16, *P* = 0.25, scrRNA vs. shRNAa7. **(Q)** Immobility time in TST. *t* = 1.88, *P* = 0.07, scrRNA vs. shRNAa7. **(R)** Immobility time in FST. *t* = 0.53, *P* = 0.61, scrRNA vs. shRNAa7. * *P* < 0.05; ** *P* < 0.01; ns not significant. Two-tailed t-tests for **(D, O**–**R)**. Two-way repeated measures ANOVAs for **(E, F)**. Two-tailed paired t-tests for **(G**–**J)**. One-way repeated measurements ANOVAs with Holm-Šídák’s multiple comparisons test for **(K**–**N)**. Two-tailed *t*-tests for **(O**–**R)**. Data are available in S1 Data as a part of Supporting information.
(TIF)

S13 Fig Functional verification of modulation of *Chrna4* and *Chrna7* genes in VP neurons. **(A, B)** Schematic diagram for injection of viral vector carrying *shrna7* gene into the VP of Vgat-Cre mice and patch-clamp recording of puff of PNU-282987-evoked nAChR currents on glutamatergic neurons. **(C, D)** Representative traces and summary showing downregulation of α7 nAChR-mediated currents in 25 GABAergic neurons from 5 Chrna7 mice relative to those in 25 neurons from 5 scrRNA mice. *t* = 4.16, *P* < 0.001. **(E)** Quantification of mRNA levels of nAChR α4 and α7 subunit on VP glutamatergic neurons from shrna7 (*n* = 4) and scrRNA (*n* = 4) mice (2 samples from each mouse). *α4*: *t* = 0.78, *P* = 0.44, scrRNA vs. shrna7; *α7*: *t* = 2.75, *P* = 0.02, scrRNA vs. shrna7. **(F, G)** Schematic diagram for injection of viral vector carrying *Chrna4* gene into the VP of Vglut2-Cre mice and patch-clamp recording of puff of RJR-2403-evoked nAChR currents on glutamateregic neurons. **(H, I)** Representative traces and summary showing upregulation of α4 nAChR-mediated currents in 31 neurons from 5 Chrna4 mice relative to those in 31 neurons from 5 eGFP mice. *t* = 4.71, *P* < 0.001. **(J)** Quantification of mRNA levels of nAChR α4 and α7 subunit on VP glutamatergic neurons from Chrna4 (*n* = 8) and eGFP (*n* = 8) mice. Interaction: *F*_(1, 28)_ = 31.40, *P* < 0.001; Row Factor: *F*_(1, 28)_ = 30.45, *P* < 0.001; Column Factor: *F*_(1, 28)_ = 31.31, *P* < 0.001. *α4*: *t* = 7.92, *P* < 0.001, eGFP vs. Chrna4; *α7*: *t* = 0.005, *P* > 0.99, eGFP vs. Chrna4. **(K, L)** Schematic diagram for injection of viral vector carrying *Chrna7* gene into the VP of Vglut2-Cre mice and patch-clamp recording of puff of RJR-2403-evoked nAChR currents on glutamatergic neurons. **(M, N)** Representative traces and summary showing upregulation of α4 nAChR-mediated currents in 30 neurons from 5 Chrna7 mice relative to those in 30 neurons from 5 eGFP mice. *t* = 8.47, *P* < 0.001. **(O)** Quantification of mRNA levels of nAChR α4 and α7 subunit on VP glutamatergic neurons from Chrna7 (*n* = 8) and eGFP (*n* = 8) mice. Interaction: F_(1, 28)_ = 150.7, *P* < 0.001; Row Factor F_(1, 28)_ = 120.0, *P* < 0.001; Column Factor: F_(1, 28)_ = 132.3, *P* < 0.001. *α4*: *t* = 0.55, *P* = 0.83, eGFP vs. Chrna4; *α7*: *t* = 16.81, *P* < 0.001, eGFP vs. Chrna4. ** *P* < 0.01; ns, not significant. Two-tailed *t*-tests for **(D, I)**. Two-way ANOVAs with Holm-Sidak multiple comparison tests for **(E, J)**. Data are available in S1 Data as a part of Supporting information.
(TIF)

S14 Fig Anterograde tracing of the downstream nuclei of VP cholinergic neurons. **(A)** Schematic diagram for viral vector-assisted anterograde tracing of VP cholinergic neurons by injecting AAV-EF1α-DIO-mGFP-Synaptophysin-mRuby into the VP of ChAT-Cre mice. **(C)** The VP was labeled with mGFP, mRuby, and ChAT-antibody (cyan). **(B, D)** Representative images and summary of downstream nuclei of VP cholinergic neurons. Summary data were from 3 slices of 3 mice. Data are available in S1 Data as a part of Supporting information. Acbc: the core of the nucleus accumbens: APir: anterior piriform cortex; BLA: basolateral amygdala; BLP: basolateral amygdala posterior part; mPFC: medial prefrontal cortex.
(TIF)

S15 Fig Anterograde tracing of the downstream nuclei of VP GABAergic neurons. **(A)** Schematic diagram for viral vector-assisted anterograde tracing of VP GABAergic neurons by injecting AAV-EF1α-DIO-mGFP-Synaptophysin-mRuby into the VP of Vgat-Cre mice. **(B)** VP neurons were labeled with mGFP, mRuby, and GABA-antibody (cyan). **(C**–**E)** Summary and representative images showing the ipsilateral and contralateral downstream nuclei of VP GABAergic neurons. Summary data were from 3 slices of 3 mice. Fluorescence intensity was normalized to that of the whole brain. Data are available in S1 Data as a part of Supporting information. ACb: nucleus accumbens; ACC: anterior cingulate cortex; AMV/LD: anterior medioventral/ lateral dorsal nuclei of the thalamus; CeA: central amygdala; DR: dorsal raphe; LC: locus coerulus; LS: lateral septum; MD: medial dorsal nucleus of the thalamus; mPFC: medial prefrontal cortex; MS/LS: medial/ lateral septum; PAG: periaqueductal gray; PB: parabrachial nucleus; PF: parafascicular nucleus of the thalamus; PIC: posterior insular cortex; PVA: paraventricular area in the hypothalamus; Re: reunion nucleus of the thalamus; SNc: substantia nigra pars compacta; STN: subthalamic nucleus; SuMM: supramammillary nucleus, medial part; VTA: ventral tegmental area. Fluorescence intensity was normalized to that of the whole brain.
(TIF)

S16 Fig Anterograde tracing of the downstream nuclei of VP glutamatergic neurons. **(A)** Schematic diagram for viral vector-assisted anterograde tracing of VP glutamatergic neurons by injecting AAV-EF1α-DIO-mGFP-Synaptophysin-mRuby into the VP of CaMKII-Cre mice. **(B)** The VP was labeled with mGFP, mRuby and CaMKII-antibody (cyan). **(C**–**E)** Summary and representative images showing the ipsilateral and contralateral downstream nuclei of VP glutamatergic neurons. Summary data were from 3 slices of 3 mice. Fluorescence intensity was normalized to that of the whole brain. Data are available in S1 Data as a part of Supporting information. ACb: nucleus accumbens; ACC: anterior cingulate cortex; AMV/LD: anterior medioventral/ lateral dorsal nuclei of the thalamus; CeA: central amygdala; DR: dorsal raphe; LC: locus coerulus; MD: medial dorsal nucleus of the thalamus; mPFC: medial prefrontal cortex; MS/LS: medial/ lateral septum; PAG: periaqueductal gray; PB: parabrachial nucleus; PF: parafascicular nucleus of the thalamus; PVA: paraventricular area in the hypothalamus; Re: reunion nucleus of the thalamus; SNc: substantia nigra pars compacta; STN: subthalamic nucleus; SuMM: supramammillary nucleus, medial part; VTA: ventral tegmental area. Fluorescence intensity was normalized to that of the whole brain.
(TIF)

S17 Fig VP neurons project to glutamatergic and GABAergic neurons in the contralateral VP. **(A)** Schematic diagrams for microinjection of transsynaptic anterograde viral vector (AAV1/2-DIO-Flp) into the left VP and AAV-hSyn-fDIO-mCherry into the right VP of Vglut2-Cre mice. **(B)** Representative images showing overlapping of mCherry(+) and CaMKII-antibody-stained neurons. **(C)** Representative images showing the overlapping of mCherry(+) neurons and CaMKII(+) neurons. Summary data were from 6 slices of 3 mice. **(D, E)** Representative images and summary showing the distribution of mCherry(+) neurons in the right VP. Summary data were from 9 slices of 3 mice. **(F)** Schematic diagrams for microinjection of transsynaptic anterograde viral vector (AAV1/2-DIO-Flp) into the left VP and AAV-hSyn-fDIO-mCherry into the right VP of Vgat-Cre mice. **(G)** Representative images showing overlapping of mCherry(+) and GABA(+) neurons. **(H)** Representative images showing the overlapping of mCherry(+) neurons and GABA(+) neurons. Summary data were from 8 slices of 4 mice. **(I, J)** Representative images and summary showing the distribution of mCherry(+) neurons in the right VP. Summary data were from 12 slices of 3 mice. Data are available in S1 Data as a part of Supporting information.
(TIF)

S18 Fig Stimulation of VP cholinergic neurons increases firing in VP GABAergic neurons. **(A)** Schematic diagram images for injection of AAV-DIO-ChR2-eYFP and AAVA-GAD67-mCherry into the VP of ChAT-Cre mice. **(B)** Representative images showing eYFP- (green) and mCherry- (red) labeled neurons in the VP 3 weeks after virus injection. **(C**–**E)** Representative traces and summary of firing in VP GABAergic neurons before, during, and after 5 s 20 Hz optogenetic stimulation of VP cholinergic neurons. **(D)** Increase firing. *F*_(1.696, 86.51)_ = 41.31, *P* < 0.001, *n* = 52; *t* = 7.35, *P* < 0.001, before vs. during light; *t* = 6.88, *P* < 0.001, during vs. after light. **(E)** No effect. *F*_(1.756, 35.12)_ = 0.14, *n* = 21, *P* = 0.85. **(F**–**H)** Representative traces and summary of firing in VP GABAergic neurons before, during, and after 10 s 20 Hz optogenetic stimulation of VP cholinergic neurons. **(G)** Increase firing. *F*_(1.629, 52.12)_ = 14.34, *n* = 33, *P* < 0.001; *t* = 4.39, *P* < 0.001, before vs. after light; *t* = 3.31, *P* = 0.003, during s. after light; *t* = 2.69, *P* = 0.01, before vs. after light. **(H)** No effect. *F*_(1.376, 24.77)_ = 2.39, *n* = 19, *P* = 0.13. * *P* < 0.05, ** *P* < 0.01, ns not significant. One-way repeated-measures ANOVAs for **(D, E, G, H)**. Data are available in S1 Data as a part of Supporting information.
(TIF)

S19 Fig VP cholinergic neurons modulate ipsilateral pain thresholds through the contralateral projections from local glutamatergic and GABAergic neurons. **(A)** Schematic diagram and timeline for optogenetic stimulation of VP cholinergic neurons and chemogenetic inhibition of contralateral projections from VP glutamatergic or GABAergic neurons. AAV-EF1α-DIO-ChR2-eYFP/AAV-EF1α-DIO-eYFP and AAV-CaMKII-hM4Di-mCherry/ AAV-GAD67-hM4Di-mCherry was injected into the right VP, and an optical fiber and a cannula were respectively implanted into the right VP and left VP. **(B**–**F)** Representative images showing ChR2-eYFP labeled cholinergic neurons in the VP **(B)**, locations of cannula above the left VP (**C**, left panel) for chemogenetic inhibition of CaMKII-hM4Di-mCherry-labeled glutamatergic terminals and optical fiber in the right VP (**C**, right panel) for optogenetic stimulation of cholinergic neurons, transfection of hM4Di-mCherry in CaMKII neurons in the right VP **(D)**. **(E)** Representative images showing locations of cannula above the left VP (left panel) for chemogenetic inhibition of GAD67-hM4Di-mCherry-labeled GABAergic terminals and optical fiber in the right VP (right panel) for optogenetic stimulation of cholinergic neurons. **(F)** Representative images showing transfection of GAD67-hM4Di-mCherry in GABAergic neurons in the right VP. **(G, H)** The specificity of viral vectors for labeling VP cholinergic neurons with ChR2-eYFP and VP CaMKII neurons with hM4Di-mCherry. **(G)** 86.28% of neurons transfected with ChR2 were cholinergic neurons. **(H)** 86.54% of neurons transfected with hM4Di were CaMKII neurons. **(I, J)** The specificity of viral vectors for labeling VP cholinergic neurons with ChR2-eYFP and VP GABAergic neurons with hM4Di-mCherry. **(I)** 83.89% of neurons transfected with ChR2 were cholinergic neurons. **(J)** 86.69% of neurons transfected with hM4Di were GABAergic neurons. Summary data were from 8 slices of 4 mice in each experiment. **(K**–**N)** PWT and PWL on either hind paw before and after blue light illumination in ChR2 and eYFP mice when saline or CNO was injected into the contralateral VP to inhibit hM4Di-labeled glutamatergic inputs. **(K)** PWT in ChR2 mice. Contralateral: *F*_(1.468, 10.28)_ = 55.74, *P* < 0.001; *t* = 7.13, *P* < 0.001, control vs. light; *t* = 1.03, *P* = 0.33, light vs. light+CNO. Ipsilateral: *F*_(1.903, 13.32)_ = 14.01, *P* < 0.001; *t* = 5.05, *P* = 0.003, control vs. light; *t* = 3.49, *P* = 0.01, light vs. light+CNO. **(L)** PWT in eYFP mice. Contralalteral: *F*_(1.958, 13.71)_ = 0.42, *P* = 0.66. Ipsilateral: *F*_(1.953, 13.67)_ = 1.12, *P* = 0.35. **(M)** PWL in ChR2 mice. Contralateral: *F*_(1.171, 8.195)_ = 134.1, *P* < 0.001; *t* = 13.11, *P* < 0.001, control vs. light; *t* = 1.76, *P* = 0.17, light vs. light+CNO. Ipsilateral: *F*_(1.727, 12.09)_ = 60.55, *P* < 0.001; *t* = 8.17, *P* < 0.001, control vs. light; *t* = 12.47, *P* < 0.001, light vs. light+CNO. **(N)** PWL in eYFP mice. Contralateral: *F*_(1.701, 11.90)_ = 1.38, *P* = 0.28. Ipsilateral: *F*_(1.626, 11.38)_ = 3.76, *P* = 0.06. **(O**–**R)** PWT and PWL on either hind paw before and after blue light illumination in ChR2 and eYFP mice when saline or CNO was injected into the contralateral VP to inhibit hM4Di-labeled GABAergic inputs. **(O)** PWT in ChR2 mice. Contralateral: *F*_(1.072, 7.501)_ = 98.19, *P* < 0.001; *t* = 10.16, *P* < 0.001, control vs. light; *t* = 2.25, *P* = 0.59, light vs. light+CNO. Ipsilateral: *F*_(1.975, 13.82)_ = 22.26, *P* < 0.001; *t* = 5.03, *P* = 0.0015, control vs. light; *t* = 6.49, *P* < 0.001, light vs. light+CNO. **(P)** PWT in eYFP mice. Contralateral: *F*_(1.590, 11.13)_ = 2.15, *P* = 0.17. Ipsilateral: *F*_(1.645, 11.52)_ = 1.57, *P* = 0.24. **(Q)** PWL in ChR2 mice. Contralateral: *F*_(1.163, 8.141)_ = 86.13, *P* < 0.001; *t* = 11.67, *P* < 0.001, control vs. light; *t* = 1.99, *P* = 0.115, light vs. light+CNO. Ipsilateral: *F*_(1.734, 12.14)_ = 32.34, *P* < 0.001; *t* = 7.61, *P* < 0.001, control vs. light; *t* = 6.98, *P* < 0.001, light vs. light+CNO. **(R)** PWL in eYFP mice. Contralateral: *F*_(1.903, 13.32)_ = 0.43, *P* = 0.64. Ipsilateral: *F*_(1.928, 13.50)_ = 0.75, *P* = 0.49. **(S, T)** PWT and PWL on the SNI side in hM4Di (*n* = 8) and mCherry (*n* = 8) mice before and after SNI surgery and injection of CNO into the contralateral VP to inhibit terminals transfected with CaMKII-hM4Di-mCherry. **(S)** Ipsilateral PWT. hM4Di: *F*_(1.149, 8.043)_ = 49.17, *P* < 0.001; *t* = 8.69, *P* < 0.001, Baseline vs. SNI; *t* = 11.56, *P* < 0.001, SNI vs. SNI + CNO. mCherry: *F*_(1.006, 7.040)_ = 47.30, *P* = 0.002; *t* = 6.79, *P* < 0.001, Baseline vs. SNI; *t* = 1.49, *P* = 0.32, SNI vs. SNI + CNO. **(T)** Ipsilateral PWL. hM4Di: *F*_(1.997, 13.98)_ = 83.64, *P* < 0.001; *t* = 12.0, *P* < 0.001, Baseline vs. SNI; *t* = 10.50, *P* < 0.001, SNI vs. SNI + CNO. mCherry: F_(1.151, 8.055)_ = 54.72, *P* = 0.0002; *t* = 7.7, *P* < 0.001, Baseline vs. SNI; *t* = 0.55, *P* = 0.84, SNI vs. SNI + CNO. **(U, V)** PWT and PWL on the SNI side in hM4Di (*n* = 8) and mCherry (*n* = 8) mice before and after SNI surgery and injection of CNO into the contralateral VP to inhibit terminals transfected with GAD67-hM4Di-mCherry. **(U)** Ipsilateral PWT. hM4Di: *F*_(1.644, 11.51)_ = 39.32, *P* < 0.001; *t* = 7.32, *P* < 0.001, Baseline vs. SNI; *t* = 7.34, *P* < 0.001, SNI vs. SNI + CNO. mCherry: *F*_(1.003, 7.019)_ = 124.0, *P* < 0.001; *t* = 11.26, *P* < 0.001, Baseline vs. SN; *t* = 0.46, *P* = 0.88, SNI vs. SNI + CNO. **(V)** Ipsilateral PWL. hM4Di: *F*_(1.746, 12.22)_ = 42.02, *P* < 0.001; *t* = 10.94, *P* < 0.001, Baseline vs. SNI; *t* = 6.42, *P* < 0.001, SNI vs. SNI + CNO. mCherry: *F*_(1.252, 8.764)_ = 74.43, *P* < 0.001; *t* = 9.65, *P* < 0.001, Baseline vs. SNI; *t* = 0.8, *P* = 0.69, SNI vs. SNI + CNO. * *P* < 0.05; ** *P* < 0.01; ns not significant. One-way repeated measures ANOVAs with Bonferroni methods for **(K**–**V)**. Data are available in S1 Data as a part of Supporting information.
(TIF)

S1 Table Primer sequences for genes measured in qRT-PCR analysis. (DOCX)

S1 Data Source data for main figures and supporting figures. (XLSX)

S1 Raw Images The raw image from which images in S8F Fig were cropped with minimal adjustment. (PDF)

## Abbreviations

ACh: acetylcholine
ACSF: artificial cerebrospinal fluid
AUC: area under the curve
CNO: clozapine-N-oxide
DB: diagonal band of Broca
EPM: Elevated plus maze
EPSCs: excitatory postsynaptic currents
FST: forced swim test
Glu: glutamatergic
MEC: mecamylamine
nAChRs: nicotinic acetylcholine receptors
NBM: nucleus basalis of Meynert
OFT: open field tests
PBS: phosphate-buffered saline
PPN: pedunculopontine tegmental nucleus
PWL: paw withdrawal latency
PWT: paw withdrawal threshold
qRT-PCR: quantitative real-time PCR
sACSF: sucrose-based artificial cerebral spinal fluid
SD: standard deviation
SEM: standard error of the mean
SNI: Spared nerve injury
STN: subthalamic nucleus
TST: tail suspension test
TTX: tetrodotoxin
VP: ventral pallidum
4-AP: 4-aminopyridine
