# Supplementary figures and images for: The subthalamic nucleus-ventral pallidum projection targeting cholinergic circuits modulates chronic pain
Source: PLoS Biol. 2026 Aug 3;24(8):e3003923. doi: 10.1371/journal.pbio.3003923 (PMC13432146; doi:10.1371/journal.pbio.3003923)

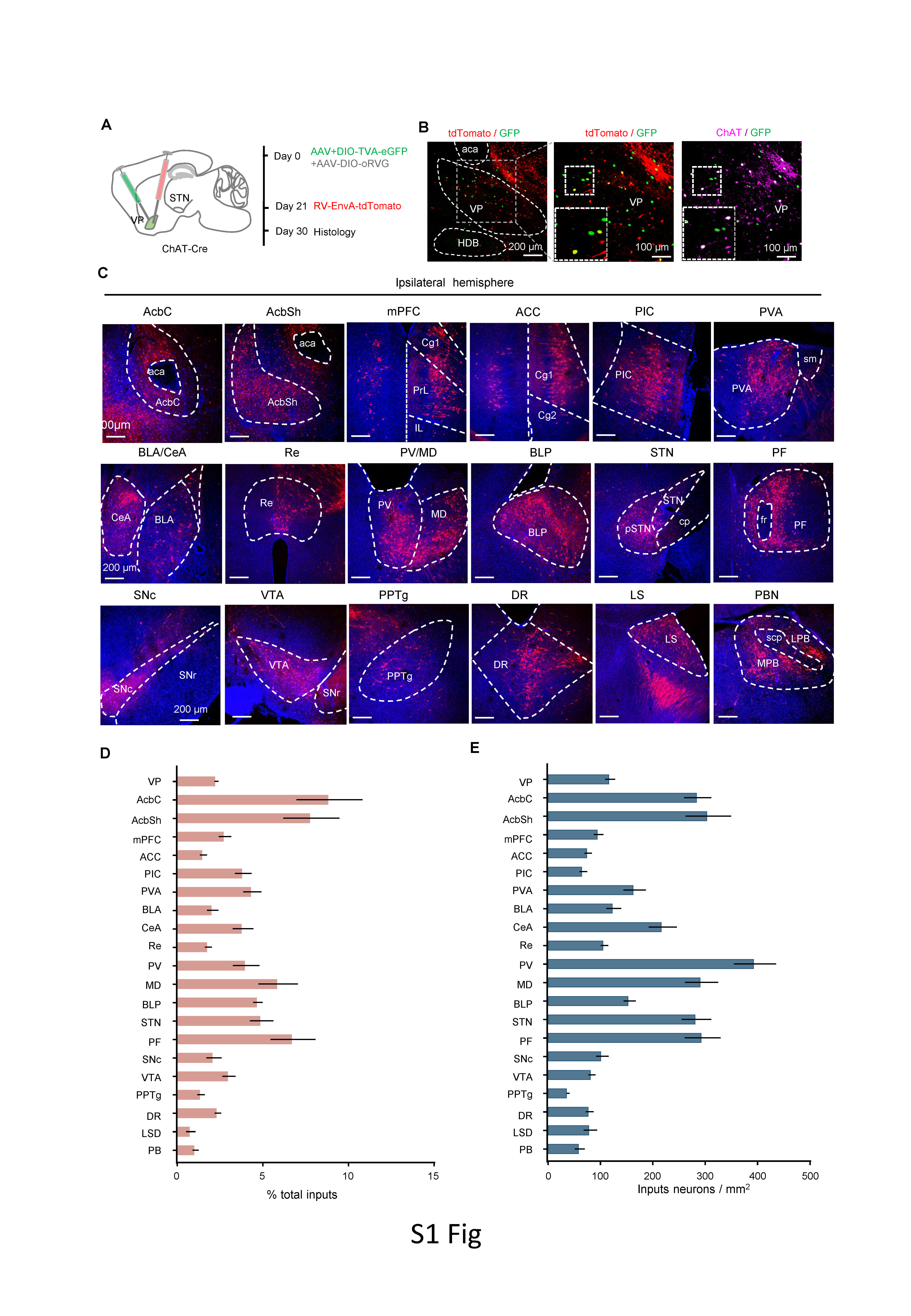

Supplement: S1 Fig — (A) Schematic diagram and timeline of retrograde tracing of upstream neurons innervating VP cholinergic neurons with AAV2/9-EF1α-DIO-TVA-GFP, AAV2/9- EF1α-DIO-oRVG, and RV-EnvA-tdTomoato. (B) Representative images showing GFP(+) neurons (green), ChAT(+) (purple), and tdTomato(+) (red) neurons in the VP. (C) Representative images showing nuclei containing tdTomato(+) neurons (red) in hemispheres ipsilateral to the starter VP cholinergic neurons. Blue, DAPI. (D, E) Summary showing % total inputs and density of tdTomato(+) neurons in hemispheres ipsilateral to the starter VP cholinergic neurons. n = 5 from 5 mice. Data are available in S1 Data as a part of Supporting information. Acb: nucleus accumbens; ACC: anterior cinglulate cortex; BLA: basolateral amygdala; BLP: basolateral amygdala posterior part; CeA: central amygdala; DR: dorsal raphe nucleus; LS: lateral septum; PBN: parabrachial nucleus; PF: parafascicular nucleus of the thalamus; PIC: posterior insular cortex; PPTg: pedunculopontine tegmental nucleus; PVA: paraventricular area of the hypothalamus; Re: reunion nucleus of the thalamus; PV/MD: Paraventricular/mediodorsal area in the thalamus; SNc: substantia nigra pars compacta; VP: ventral pallidum; VTA: ventral tegmental area; mPFC: medial prefrontal cortex. (TIF) [file pbio.3003923.s001.tif]

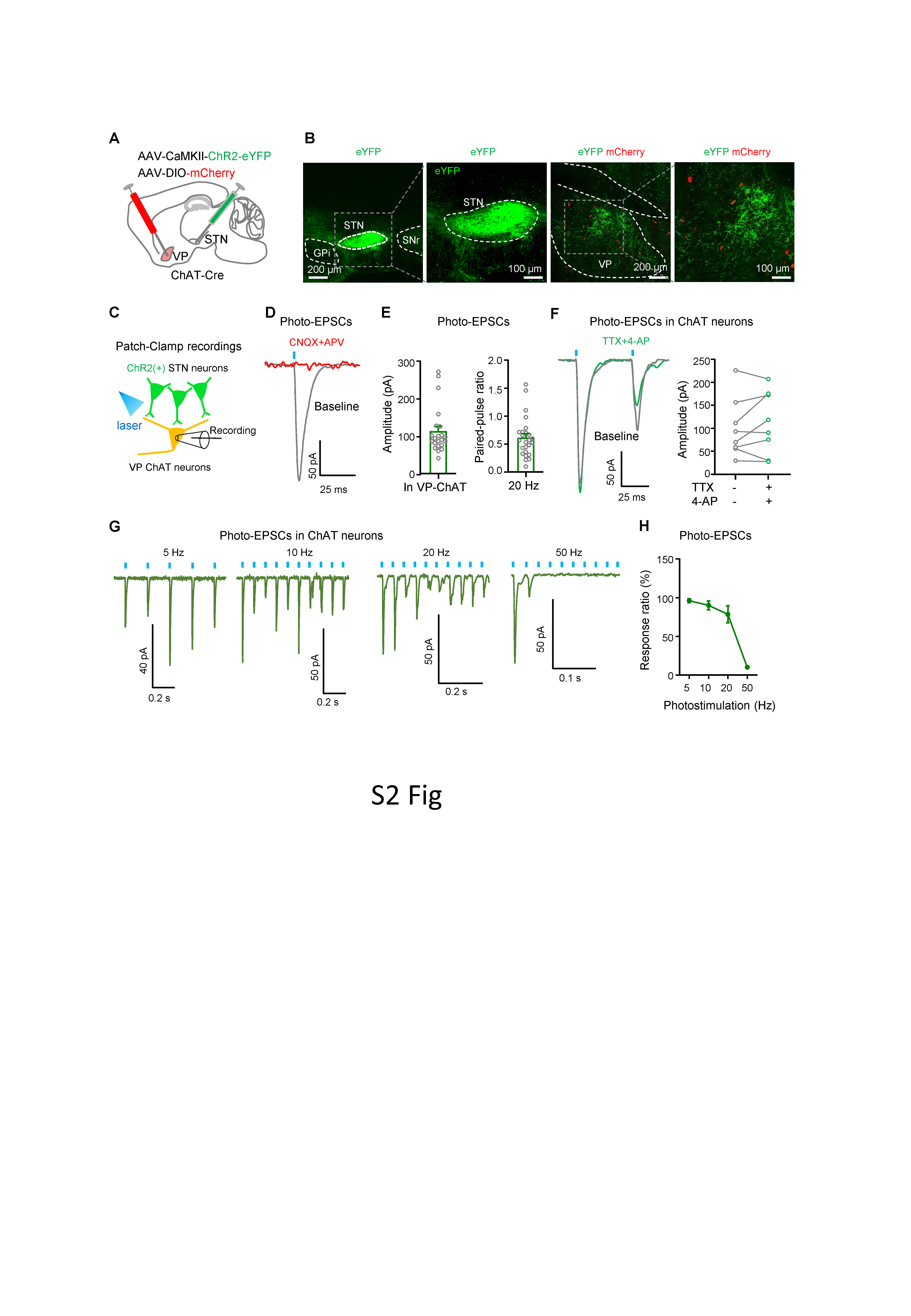

Supplement: S2 Fig — (A) Schematic diagram for virus injection to fulfill optogenetic stimulation of the STN-VP glutamatergic projection (AAV2/9-CaMKII-ChR2-eYFP in the STN) and cell-specific fluorescence labeling of cholinergic neurons (AAV2/9-EF1α-DIO-mCherry in the VP) in ChAT-Cre mice. (B) Representative images showing transfection of CaMKII-ChR2-eYFP and mCherry into the STN and VP. (C) Diagram for patch-clamp recording of VP cholinergic neurons upon optogenetic stimulation of the STN-VP glutamatergic projection. (D) Representative traces showing blue light-evoked inward currents sensitive to CNQX (20 μM) and APV (50 μM). (E) Summary of amplitude and paired-pulse (with 50 ms interval) ratio of photo-EPSCs in VP cholinergic neurons (n = 22–25). (F) Representative traces and summary of postsynaptic currents evoked by paired blue light pulses (50 ms apart) in VP cholinergic neurons before and during incubation of 1 μM tetrodotoxin (TTX) and 0.3 mW 4-aminopyridine (4-AP). n = 8. (G, H) Representative traces of postsynaptic currents evoked by blue light pulses (5, 10, 20, and 50 Hz) and success rate for light-evoked currents, n = 10. Data from 3 mice. Data are available in S1 Data as a part of Supporting information. (TIF) [file pbio.3003923.s002.tif]

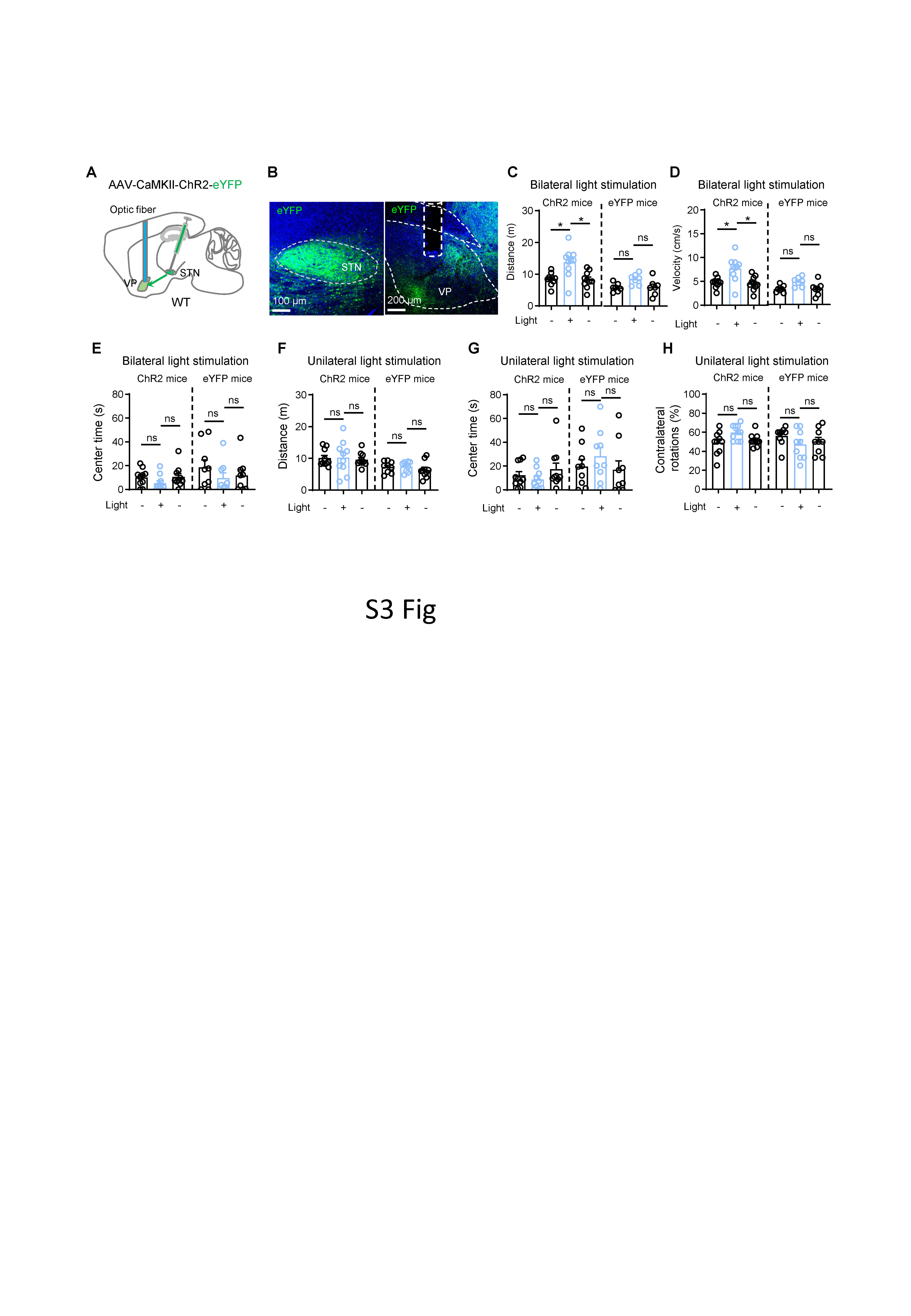

Supplement: S3 Fig — (A) Schematic diagram for injecting AAV-CaMKII-ChR2-eYFP (ChR2) or AAV-CaMKII-eYFP (eYFP) and implanting optical fiber into the STN in ChAT-Cre mice. (B) Representative images showing eYFP-labeled STN neurons and their projections to the VP, and optical fiber track targeting the VP. (C, D) Total distance and velocity in the open field tests in ChR2 (n = 9) and eYFP (n = 9) mice before, during, and after bilateral blue light illumination of the VP. (C) Distance. ChR2: F(1.371, 12.34) = 9.69, P = 0.005; t = 3.37, P = 0.02, control vs. light. eYFP: F(1.901, 11.41) = 5.22, P = 0.03. t = 2.82, P = 0.09, control vs. light. (D) Velocity. ChR2: F(1.901, 11.41) = 5.22, P = 0.006; t = 3.35, P = 0.03, control vs. light. eYFP: F(1.916, 11.50) = 5.62, P = 0.02. t = 2.89, P = 0.08, control vs. light. (E) Time in the center zone in the open field test in ChR2 and eYFP mice before, during, and after bilateral blue light illumination of the VP. ChR2: F(1.656, 14.90) = 1.82, P = 0.20. eYFP: F(1.123, 8.987) = 2.83, P = 0.12. (F, G) Total distance and time in the center zone in the open field tests in ChR2 (n = 9) and eYFP (n = 9) mice before, during, and after unilateral blue light illumination of the VP. (F) Total distance. ChR2: F(1.232, 11.09) = 0.08, P = 0.83. eYFP: F(1.806, 14.45) = 1.06, P = 0.36. (G) Time in the central zone. ChR2: F(1.806, 16.26) = 2.67, P = 0.10. eYFP: F(1.297, 10.37) = 1.06, P = 0.35. (H) % of contralateral rotation in the open field tests in ChR2 (n = 9) and eYFP (n = 9) mice before, during, and after unilateral blue light illumination of the VP. * P < 0.05. ns not significant. One-way repeated measures ANOVAs for (C-H). Data are available in S1 Data as a part of Supporting information. (TIF) [file pbio.3003923.s003.tif]

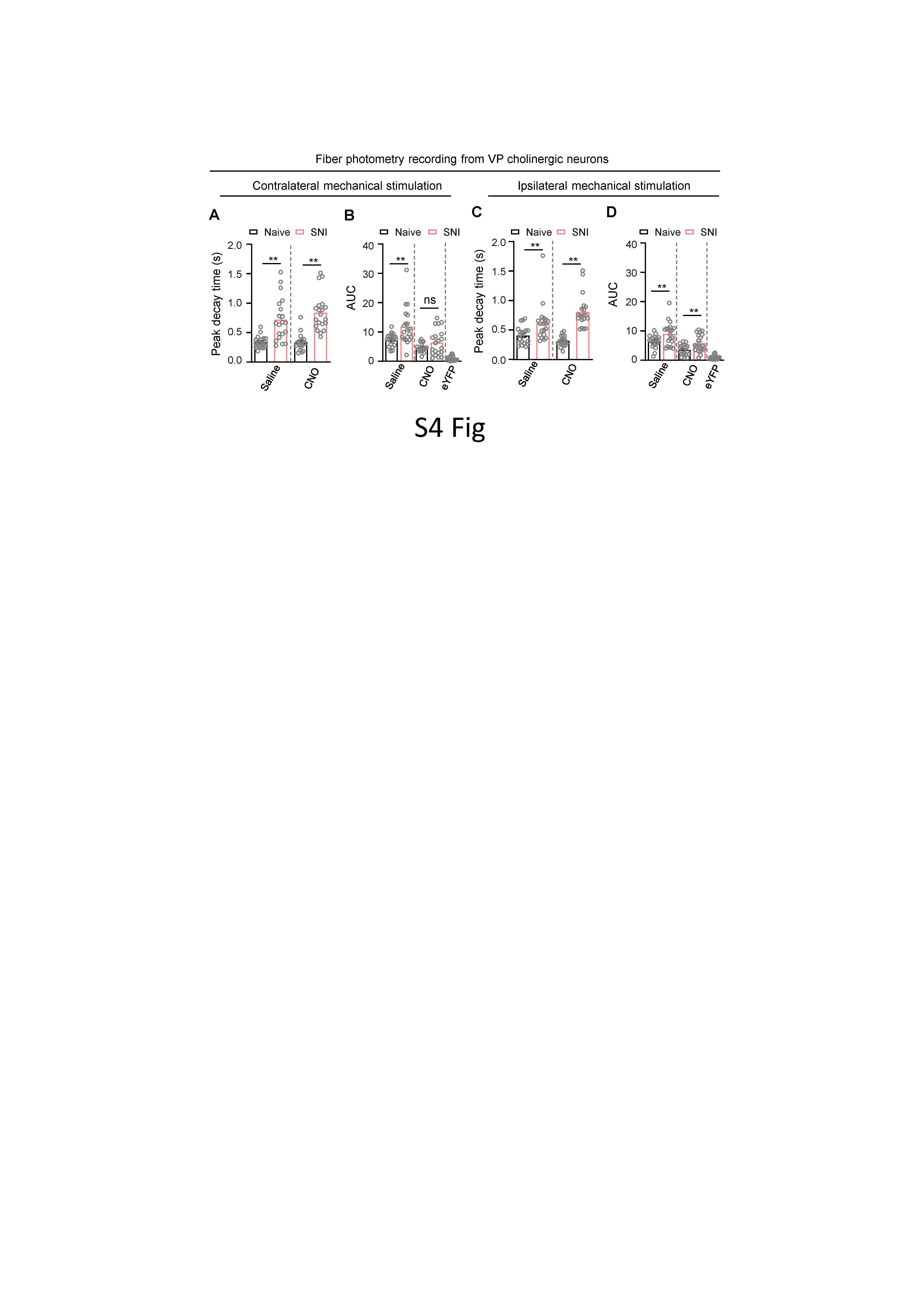

Supplement: S4 Fig — This figure presents decay time and area under the curve (AUC) of responses in VP cholinergic neurons to mechanical stimulation on hind paws using the same datasets for Fig 2F–2O. (A, B) Decay time and area under the curve of responses of of VP cholinergic neurons to mechanical stimulation on the contralateral hind paw before (saline) and during (CNO) chemogenetic inhibition of the STN-VP projection. (A) Decay time. F(3, 76) = 21.08, P < 0.001. Saline: t = 4.74, P < 0.001, Naive vs. SNI. CNO: t = 6.3, P < 0.001, Naive vs. SNI. n = 20. (B) AUC. F(4, 95) = 24.86, P < 0.001. Saline: t = 4.83, P < 0.001, Naive vs. SNI. CNO: t = 2.39, P = 0.01, Naive vs. SNI. n = 20. (C, D) Decay time and area under the curve of responses of of VP cholinergic neurons to mechanical stimulation on the ipsilateral hind paw before (saline) and during (CNO) chemogenetic inhibition of the STN-VP projection. (C) Decay time. F(3, 76) = 19.45, P < 0.001. Saline: t = 2.93, P = 0.005, Naive vs. SNI. CNO: t = 6.99, P < 0.001, Naive vs. SNI. n = 20. (D) AUC. F(4, 95) = 28.82, P < 0.001. Saline: t = 3.28, P = 0.003, Naive vs. SNI. CNO: t = 2.78, P = 0.006, Naive vs. SNI. n = 20. ** P < 0.01. One-way ANOVAs for (A–D). Data are available in S1 Data as a part of Supporting information. (TIF) [file pbio.3003923.s004.tif]

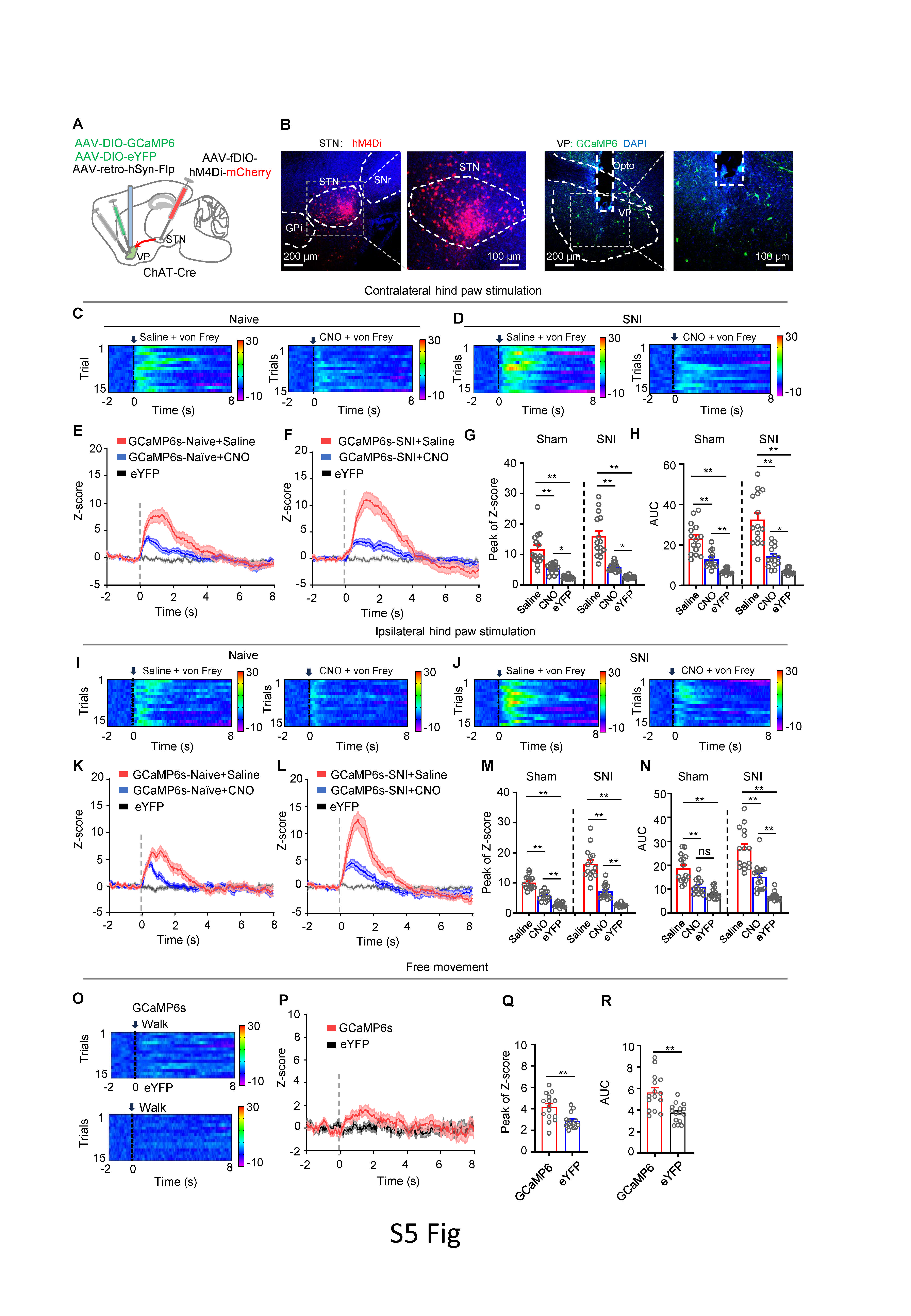

Supplement: S5 Fig — (A, B) Schematic diagram and representative images for injecting AAV-EF1α-DIO-GCaMP6s or AAV-EF1α-DIO-eYFP and implanting optical fiber into the VP in ChAT-Cre mice. Meanwhile, AAV retro-hSyn-Flp was injected into the VP, and AAV-fDIO-hM4Di-mCherry was injected into the STN to transfect hM4Di into VP-projecting STN neurons. (C–H) Heat maps, averaged traces, and summary showing changes in GCaMP6s (n = 5 mice) and eYFP (n = 5 mice) in response to suprathreshold von Frey filament stimulation onto the contralateral hind paw of naïve and SNI mice before and after intraperitoneal injection of CNO (3 mg/kg). (G) Contralateral stimulation evoked peak z-score. Naive, F(2, 42) = 32.37, P < 0.001; t = 5.41, P < 0.001, Saline vs. CNO. SNI, F(2, 42) = 44.76, P < 0.001; t = 6.82, P < 0.001, Saline vs. CNO. (H) Contralateral stimulation evoked peak AUC. Naive, F(2, 42) = 22.47, P < 0.001; t = 4.51, P < 0.001, Saline vs. CNO. SNI, F(2, 42) = 31.74, P < 0.001; t = 5.34, P < 0.001, Saline vs. CNO. (I–N) Heat maps, averaged traces, and summary showing changes in GCaMP6s and eYFP in response to suprathreshold von Frey filament stimulation on the ipsilateral hind paw of naïve and SNI mice before and after intraperitoneal injection of CNO (3 mg/kg). (M) Ipsilateral stimulation evoked peak z-score. Naive, F(2, 42) = 84.62, P < 0.001; t = 7.79, P < 0.001, Saline vs. CNO. SNI, F(2, 42) = 67.72, P < 0.001; t = 7.60, P < 0.001, Saline vs. CNO. (N) Ipsilateral stimulation evoked peak AUC. Naïve, F(2, 41) = 25.11, P < 0.001; t = 5.06, P < 0.001, Saline vs. CNO. SNI, F(2, 42) = 34.95, P < 0.001; t = 5.73, P < 0.001, Saline vs. CNO. (O–R) Heat maps, representative traces, averaged traces, and summary showing changes of GCaMP6 signal during episodes of voluntary movement before and after intraperitoneal administration of CNO (3 mg/kg). (Q) Peak. t = 3.57, P = 0.002, GCaMP6 vs. eYFP. (P) AUC. t = 2.86, P = 0.008, GCaMP6 vs. eYFP. ** P < 0.01; ns not significant. One-way ANOVA for (G, H, M, N). Two- [file pbio.3003923.s005.tif]

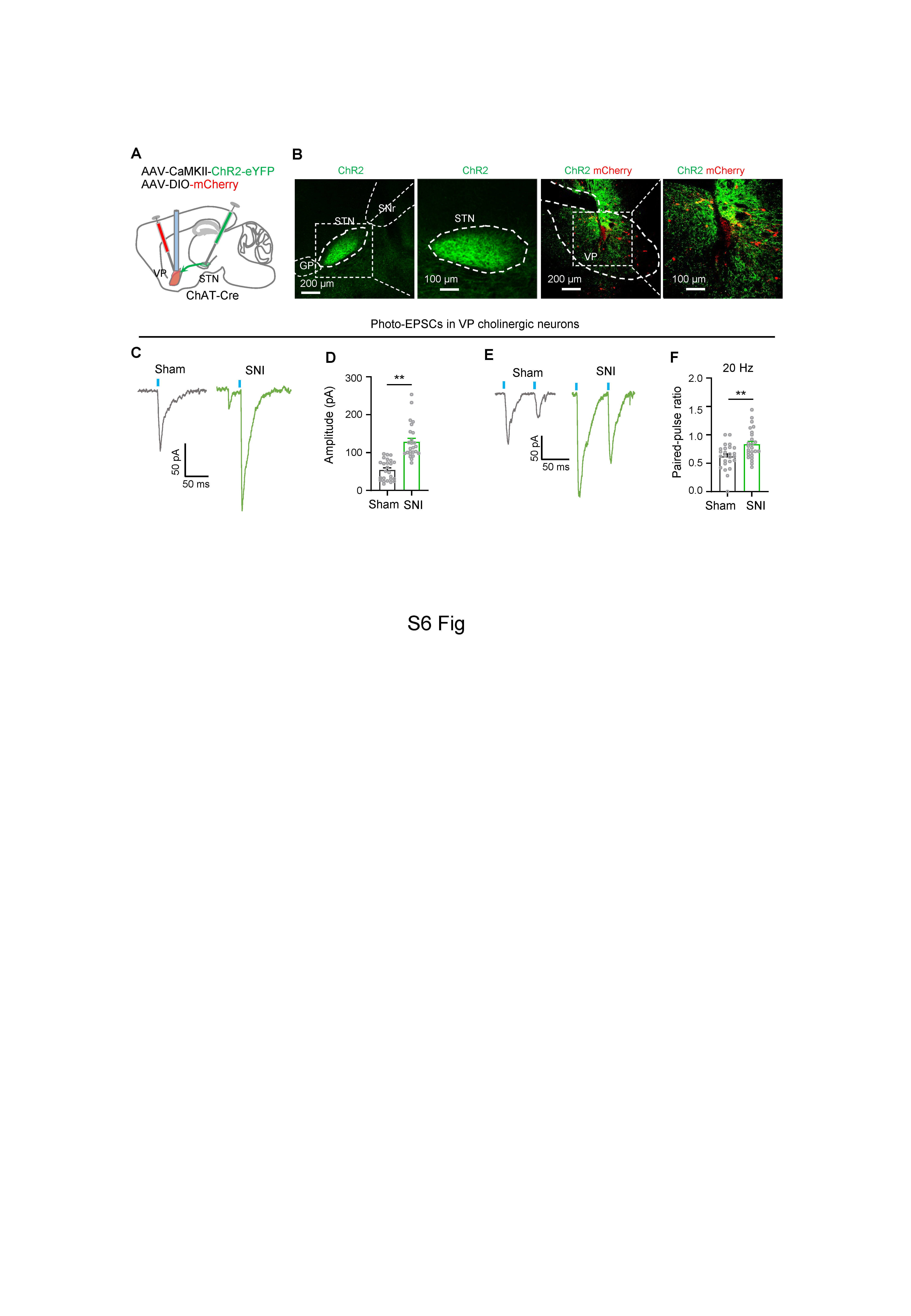

Supplement: S6 Fig — (A) Schematic diagram for virus injection to fulfill optogenetic stimulation of the STN-VP glutamatergic projection (AAV2/9-CaMKII-ChR2-eYFP in the STN) and cell-specific fluorescence labeling of cholinergic neurons (AAV2/9-EF1α-DIO-mCherry in the VP) in ChAT-Cre mice. (C, D) Representative traces and summary of amplitude of 5 ms blue light-evoked currents in VP cholinergic neurons in Sham and SNI mice. n = 25 neurons from 3 mice in each group. t = 6.99, P < 0.0001, SNI vs. sham. (E, F) Representative traces and summary of postsynaptic currents evoked by paired blue light pulses (50 ms apart) on VP cholinergic neurons from sham and SNI mice. n = 25 cholinergic neurons from each group, t = 3.09, P = 0.003, Sham vs. SNI. ** P < 0.01. Two-tailed t test for (D, E). Data are available in S1 Data as a part of Supporting information. (TIF) [file pbio.3003923.s006.tif]

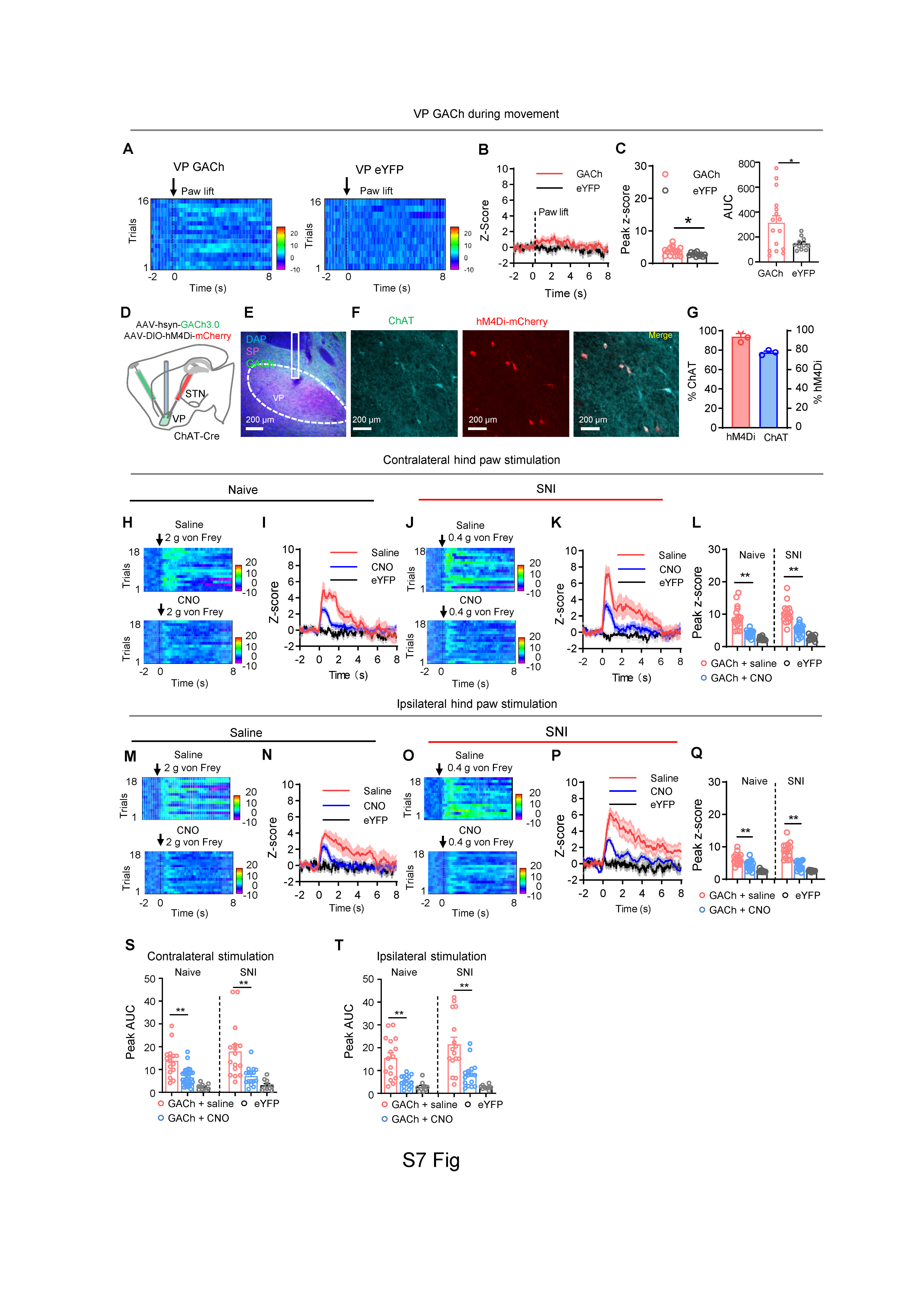

Supplement: S7 Fig — (A–C) Heat maps, averaged fluorescent intensity of GACh and eYFP in the VP during episode of voluntary movement. Sixteen trials from 8 GACh mice and 5 eYFP mice. Peak. t = 2.47, P = 0.02. AUC. t = 2.41, P = 0.02, GACh vs. eYFP. Twenty trials from 5 mice in each group. Two-tailed t-tests for (C). (D–G) Schematic diagram (D) for viral injection strategy and representative images showing transfection of GACh into the VP (E) and hM4Di-mCherry (F) into VP cholinergic neurons in ChAT-Cre mice. (G) ChAT neurons accounted for 93.9% of hM4Di-mCherry neurons. 77.78% of ChAT neurons were labeled with hM4Di-mCherry. Data from 3 slices (1 from each mouse) were summarized. (H–L) Heat maps, averaged traces, and summary showing changes in GACh (n = 6 mice) and eYFP (n = 5 mice) in response to suprathreshold von Frey filament stimulation on the contralateral hind paw of naïve and SNI mice before and after intraperitoneal injection of CNO (3 mg/kg). Naïve: F(2, 39) = 31.35, P < 0.001; t = 6.19, P < 0.001, saline vs. CNO. SNI: F(2, 39) = 42.30, P < 0.001; t = 6.49, P < 0.001, saline vs. CNO. (M–Q) Heat maps, averaged traces, and summary showing changes in GACh (n = 6) and eYFP (n = 5) in response to suprathreshold von Frey filament stimulation on the ipsilateral hind paw of naïve and SNI mice before and after intraperitoneal injection of CNO (3 mg/kg). Naïve: F(2, 39) = 23.54, P < 0.001; t = 4.10, P < 0.001, saline vs. CNO. SNI: F(2, 39) = 51.58, P < 0.001; t = 7.77, P < 0.001, saline vs. CNO. (R–S) Peak AUC of contralateral VP, ipsilateral VP in Naive and SNI and eYFP mice, before and after intraperitoneal administration of CNO (3 mg/kg). (R) Naive: F(2, 39) = 23.13, P < 0.001.; t = 4.76, P < 0.001, saline vs. CNO. SNI: F(2, 39) = 12.94, P < 0.001; t = 3.88, P < 0.001, saline vs. CNO. (S) Naive: F(2, 39) = 18.84, P < 0.001; t = 4.97, P < 0.001, saline vs. CNO. SNI: F(2, 39) = 17.67, P < 0.001; t = 4.41, P < 0.001, saline vs. CNO. * P < 0.05; ** P < 0.01; ns not significant. One-way A [file pbio.3003923.s007.tif]

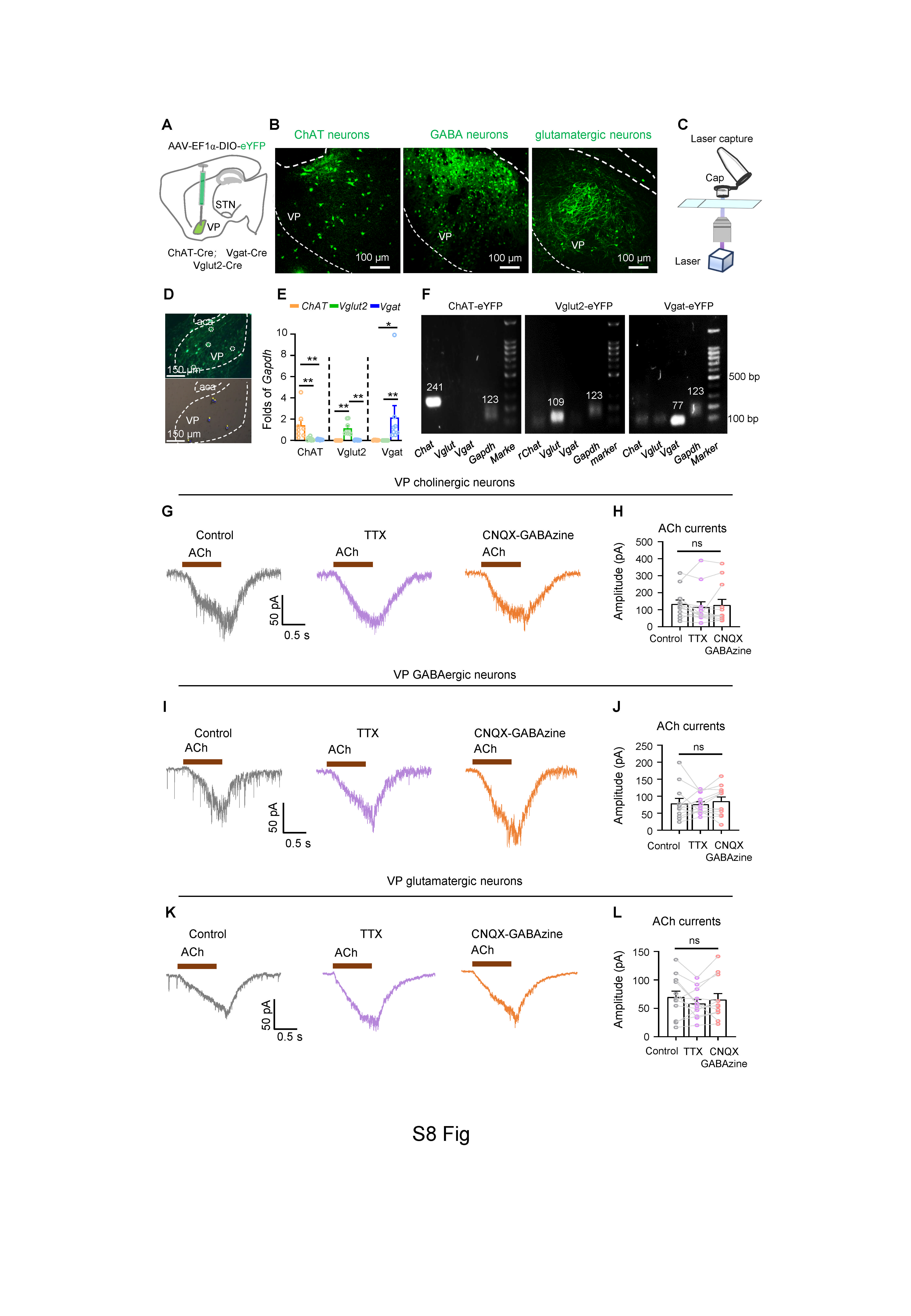

Supplement: S8 Fig — (A) Schematic diagram for labeling of VP neuron types with viral vector and Cre-mouse lines. (B) Representative images showing eYFP-labeled cholinergic, glutamatergic, and GABAergic neurons in the VP. (C, D) Schematic diagram and representative fluorescent and bright-field images showing collection of eYFP-labeled neurons in the VP under a laser capture microscope. (E) qRT-PCR was performed to analyze the mRNA of ChAT, Vglut2, and Vgat genes in eYFP-labeled ChAT, Vglut2, and Vgat neurons. ChAT neurons: F(2, 21) = 7.21, P = 0.004; t = 3.21, P = 0.008, ChAT vs. Vglut2; t = 3.36, P = 0.006, ChAT vs. Vgat). n = 8 samples. Vglut2 neurons: F(2, 21) = 24.58, P < 0.001; t = 6.15, P < 0.001, ChAT vs. Vglut2; t = 5.98, P < 0.001, Vglut2 vs. Vgat. n = 8 samples. Vgat neurons: Kruskal-Wallis U = 20.48, P < 0.001; t = 2.26, P = 0.047, ChAT vs. Vgat; t = 4.52, P < 0.001, Vglut2 vs. Vgat. n = 8 samples from 3 mouse for each type of neurons. (F) Images of gel electrophoresis results of qRT-PCR products from eYFP-labeled neurons in ChAT-Cre, Vglut2-Cre, and Vgat-Cre mice. (G, H) Representative traces and summary of amplitudes of 0.3 mM ACh-evoked inward currents in the absence and presence of 0.5 μM tetrodotoxin (TTX) or 20 μM + 10 μM GABAzine in VP ChAT neurons. F(1.812, 19.93) = 0.54, P = 0.58. n = 10. (I, J) Representative traces and summary of amplitudes of 0.3 mM ACh-evoked inward currents in the absence and presence of 0.5 μM TTX or 20 μM + 10 μM GABAzine in VP Vgat neurons. F(1.812, 19.93) = 0.54, P = 0.58. n = 10. (K, L) Representative traces and summary of amplitudes of 0.3 mM ACh-evoked inward currents in the absence and presence of 0.5 μM TTX or 20 μM + 10 μM GABAzine in VP Vglut2 neurons. F(1.293, 14.22) = 1.12, P = 0.33. n = 10. * P < 0.05, ** P < 0.01, ns not significant. One-way ANOVAs with Bonferroni tests for (E). One-way repeated measures ANOVAs with Bonferroni tests for (H, J, L). Data are available in S1 Data as a part of Supporting information. Images in (F) wer [file pbio.3003923.s008.tif]

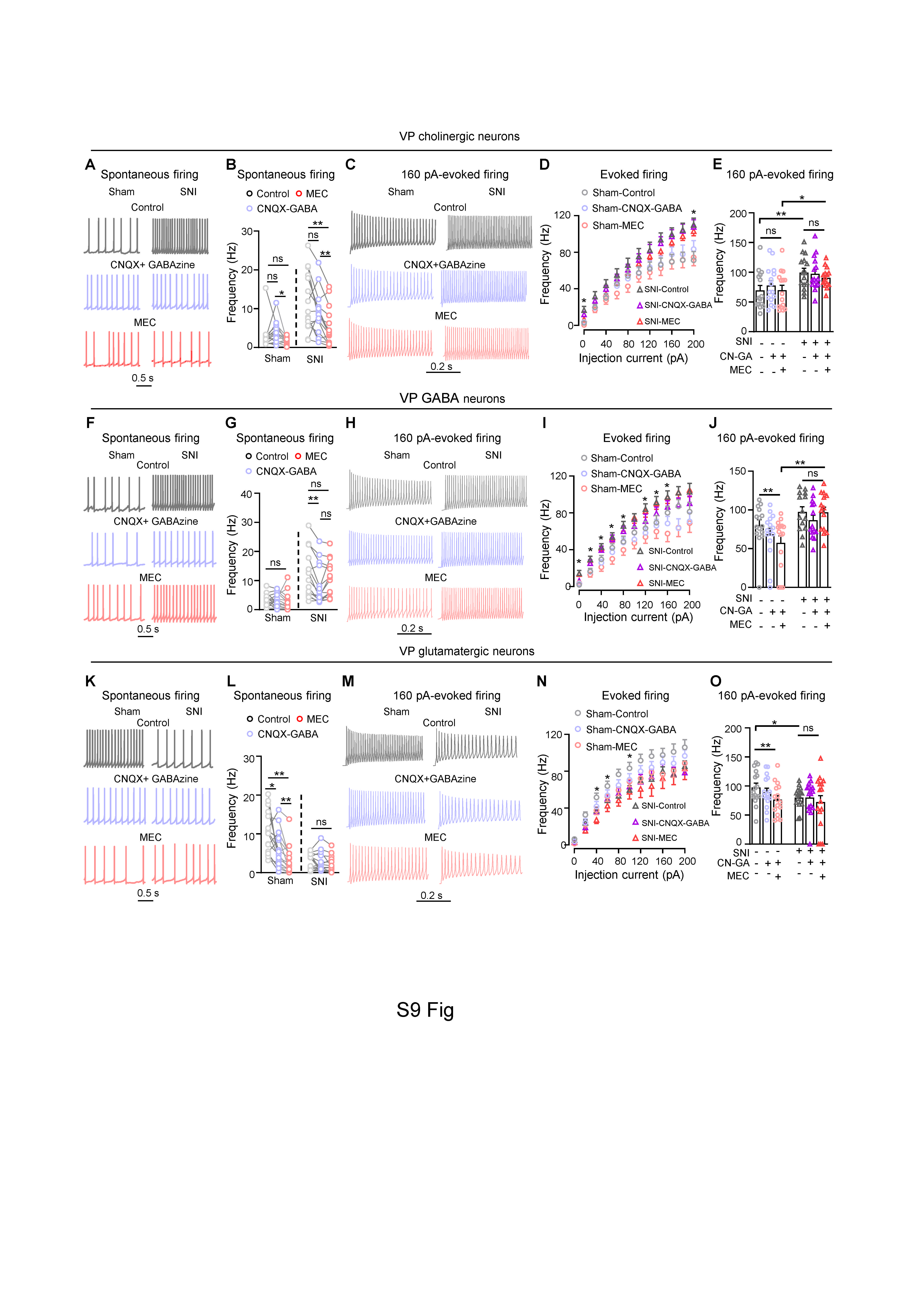

Supplement: S9 Fig — (A, B) Representative traces and summary of spontaneous firing in VP cholinergic neurons from sham and SNI mice (n = 15 neurons from 3 mice in each group) in the absence and presence of CNQX+GABAzine or MEC. Sham: F(1.798, 25.17) = 2.77, P = 0.08; t = 2.77, P = 0.04, CNQX+GABAzine vs. MEC. SNI: F(1.393, 19.51) = 12.93, P = 0.0008; t = 2.11, P = 0.05, baseline vs. CNQX+GABAzine; t = 4.42, P = 0.0014, CNQX+GABAzine vs. MEC; t = 4.51, P = 0.0014, baseline vs. MEC. (C, D) Representative traces and summary of firing evoked by depolarizing current injections in VP ChAT neurons from sham and SNI mice. Currents, F(2.083, 175.0) = 491.4, P < 0.001; Group, F(5, 84) = 3.34, P = 0.008; Interaction, F(50, 840) = 2.42, P < 0.001. Data from 15 neurons from 3 mice in each group. (E) 160 pA current-evoked firing. Drugs, F(1, 28) = 6.30, P = 0.018; Group, F(2, 56) = 1.87, P = 0.16; Interaction, F(2, 56) = 1.10, P = 0.33. Baseline, t = 2.89, P = 0.005, Sham vs. SNI. MEC, t = 2.03, P = 0.045, sham vs. SNI. n = 15 neurons from 3 mice in each group. (F, G) Representative traces and summary of spontaneous firing in VP GABAergic neurons from sham and SNI mice (n = 15 neurons from 3 mice in each group) in the absence and presence of CNQX+GABAzine or MEC. Sham: F(1.421, 19.90) = 0.59, P = 0.50. SNI: F(1.959, 27.43) = 8.49, P = 0.0014; t = 4.41, P = 0.0018, baseline vs. CNQX+GABAzine; t = 2.32, P = 0.07, CNQX+GABAzine vs. MEC; t = 1.67, P = 0.11, baseline vs. MEC. n = 15 neurons from 3 mice in each group. (H, I) Representative traces and summary of firing evoked by depolarizing current injections in VP GABA neurons from sham (n = 15) and SNI (n = 14) mice (3 in each group). Currents, F(2.327, 188.5) = 274.6, P < 0.001; Group, F(5, 81) = 5.53, P = 0.0002; Interaction, F(50, 810) = 1.26, P = 0.011. (J) 160 pA current-evoked firing. Drugs, F(1, 27) = 8.43, P = 0.007; Group, F(2, 54) = 3.55, P = 0.035; Interaction, F(2, 54) = 3.20, P = 0.048. MEC, t = 3.82, P = 0.0003, Sham vs. SNI. sham; t = 3.2 [file pbio.3003923.s009.tif]

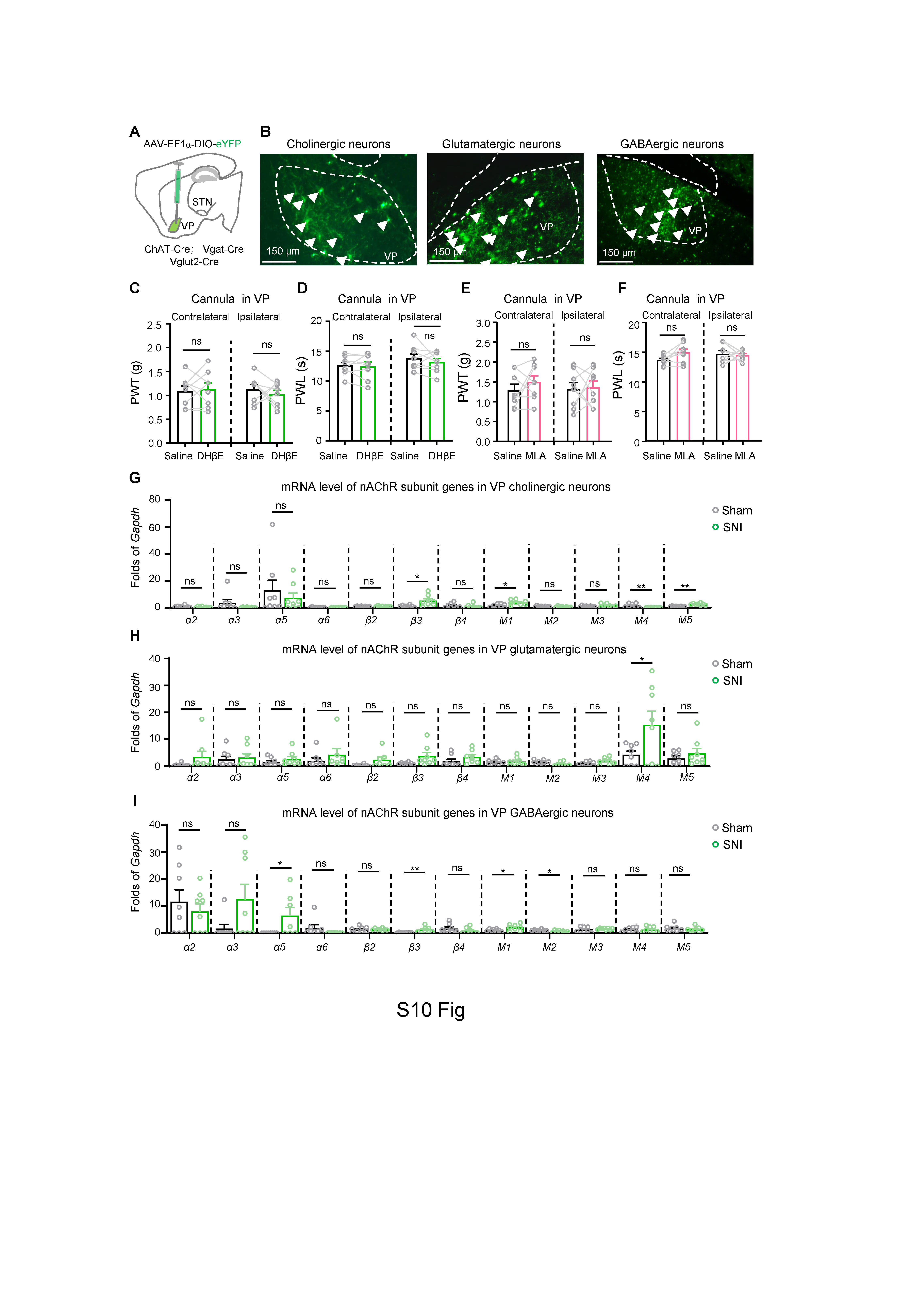

Supplement: S10 Fig — qRT-PCR was performed to analyze the acetylcholine receptor mRNAs in eYFP-labeled ChAT, glutamatergic and GABAergic neurons. The levels of nAChR and mAChR subunit genes were normalized to that of Gapdh. (A, B) Schematic diagram (A) and representative images (B) for specific labeling of cholinergic, glutamatergic, and GABAergic neurons in the VP with viral vector (AAV-EF1α-DIO-eYFP) in ChAT-Cre, Vglut2-Cre, and Vgat-Cre mice. (C, D) PWT and PWL on hind paws of mice (n = 8) before and after injection of DHβE into the VP. (C) PWT. F(2.600, 18.20) = 0.23, P = 0.85. Contralateral: t = 0.32, P = 0.76; Ipsilateral: t = 0.70, P = 0.76. (D) PWL. F(2.144, 15.01) = 2.19, P = 0.14. Contralateral: t = 0.35, P = 0.73; Ipsilateral: t = 0.91, P = 0.63. (E, F) PWT and PWL on hind paws of mice (n = 8) before and after injection of MLA into the VP. (E) PWT. F(2.421, 16.95) = 0.39, P = 0.14. Contralateral: t = 1.11, P = 0.51; Ipsilateral: t = 0.21, P = 0.84. (F) PWL. F(2.226, 15.58) = 1.23, P = 0.32. Contralateral: t = 2.19, P = 0.13; Ipsilateral: t = 0.33, P = 0.75. (G) Comparison of mRNA levels of acetylcholine receptor subunit genes in ChAT neurons between sham and SNI mice. α2: t = 1.53, P = 0.15. α3: t = 1.49, P = 0.17. α5: t = 0.68, P = 0.50. α6: t = 1, P = 0.33. β2: t = 0.007, P = 0.99. β3: t = 2.94, P = 0.01. β4: t = 0.90, P = 0.38I. M1: t = 2.93, P = 0.01. M2: t = 1.27, P = 0.23. M3: t = 1.64, P = 0.12. M4: t = 3.55, P = 0.003. M5: t = 6.35, P < 0.001. n = 8 for each type of receptor subunits in sham and mice. (H) Comparison of mRNA levels of acetylcholine receptor subunit genes in glutamatergic neurons between sham and SNI mice. α2: t = 1.49, P = 0.17. α3: t = 0.38, P = 0.71. α5: t = 1.07, P = 0.30. α6: t = 0.95, P = 0.36. β2: t = 1.96, P = 0.07. β3: t = 2.13, P = 0.05. β4: t = 1.47, P = 0.16. M1: t = 0.32, P = 0.75. M2: t = 1.67, P = 0.12. M3: t = 1.67, P = 0.11. M4: t = 2.15, P = 0.04. M5: t = 0.94, P = 0.35. n = 7–8 for each type of receptor subunits in sham and mice. (I) [file pbio.3003923.s010.tif]

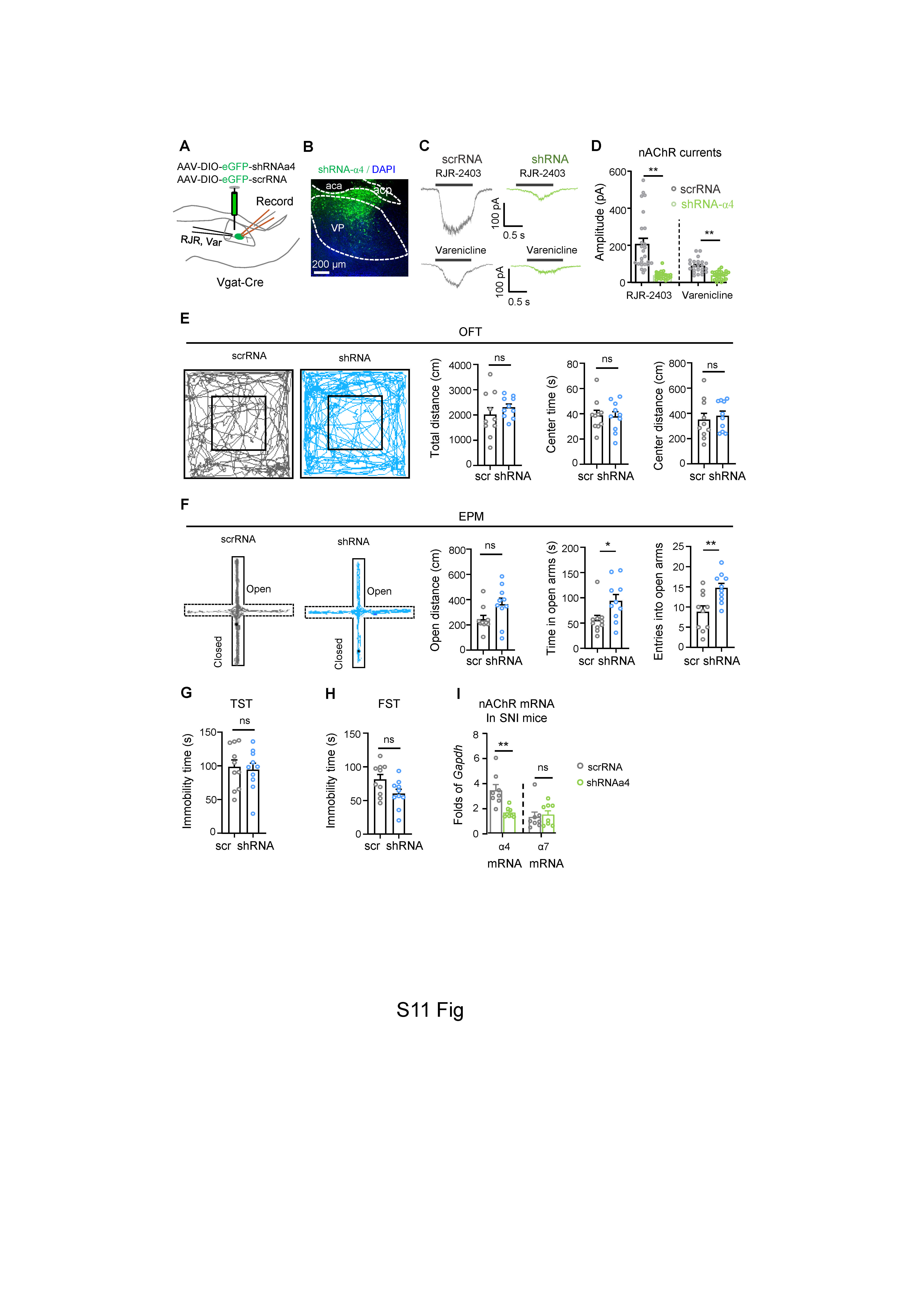

Supplement: S11 Fig — (A, B) Schematic diagram and representative image for injection of viral vector carrying Chrna4 gene shRNA into the VP of Vgat-Cre mice and patch-clamp recording of puff of RJR-2403- or varenicline-evoked nAChR currents on GABAergic neurons. (C, D) Representative traces and summary showing downregulation of α4 nAChR-mediated currents in 10 neurons from 5 shRNA mice relative to those in 10 neurons from 5 scrRNA mice. RJR-2403: t = 5.62, P < 0.001. Varenicline: t = 5.71, P < 0.001. (E–H) Mice with downregulation of α4 nAChR in VP performed locomotion, anxiety, Tail suspension test and Forced swimming test. (E) Open field tests. Total Distance: t = 0.94, P = 0.36, scrRNA vs. shRNA. Center time: t = 0.17, P = 0.86, scrRNA vs. shRNA. Center distance: t = 0.5, P = 0.62, scrRNA vs. shRNA. (F) Elevated plus maze. Open Distance: t = 2.08, P = 0.05, scrRNA vs. shRNA. Time in open arms: t = 2.38, P = 0.028, scrRNA vs. shRNA. Entries in open arms: t = 3.15, P = 0.005, scrRNA vs. shRNA. (G) Immobility time in the tail suspension tests (TST). t = 0.28, P = 0.77, scrRNA vs. shRNA. (H) Immobility time in the forced swim tests (FST). t = 2.17, P = 0.05, scrRNA vs. shRNA. (I) Quantification of mRNA levels of nAChR α4 and α7 subunits on VP GABAergic neurons from shRNAα4 (n = 5) and scrRNA (n = 5) mice. α4: t = 3.74, P = 0.001, shRNA-α4 (n = 8) vs. scrRNA (n = 8). α7: t = 0.41, P = 0.90, shRNA-α4 (n = 8) vs. scrRNA (n = 8). * P < 0.05, ** P < 0.01; ns, not significant. Two-tailed t-tests for (E–I). Two-way ANOVAs with Bonferroni tests for (D). OFT: open field test. EPM: elevated plus maze. TST: tail suspension test. FST: forced swim test. Data are available in S1 Data as a part of Supporting information. (TIF) [file pbio.3003923.s011.tif]

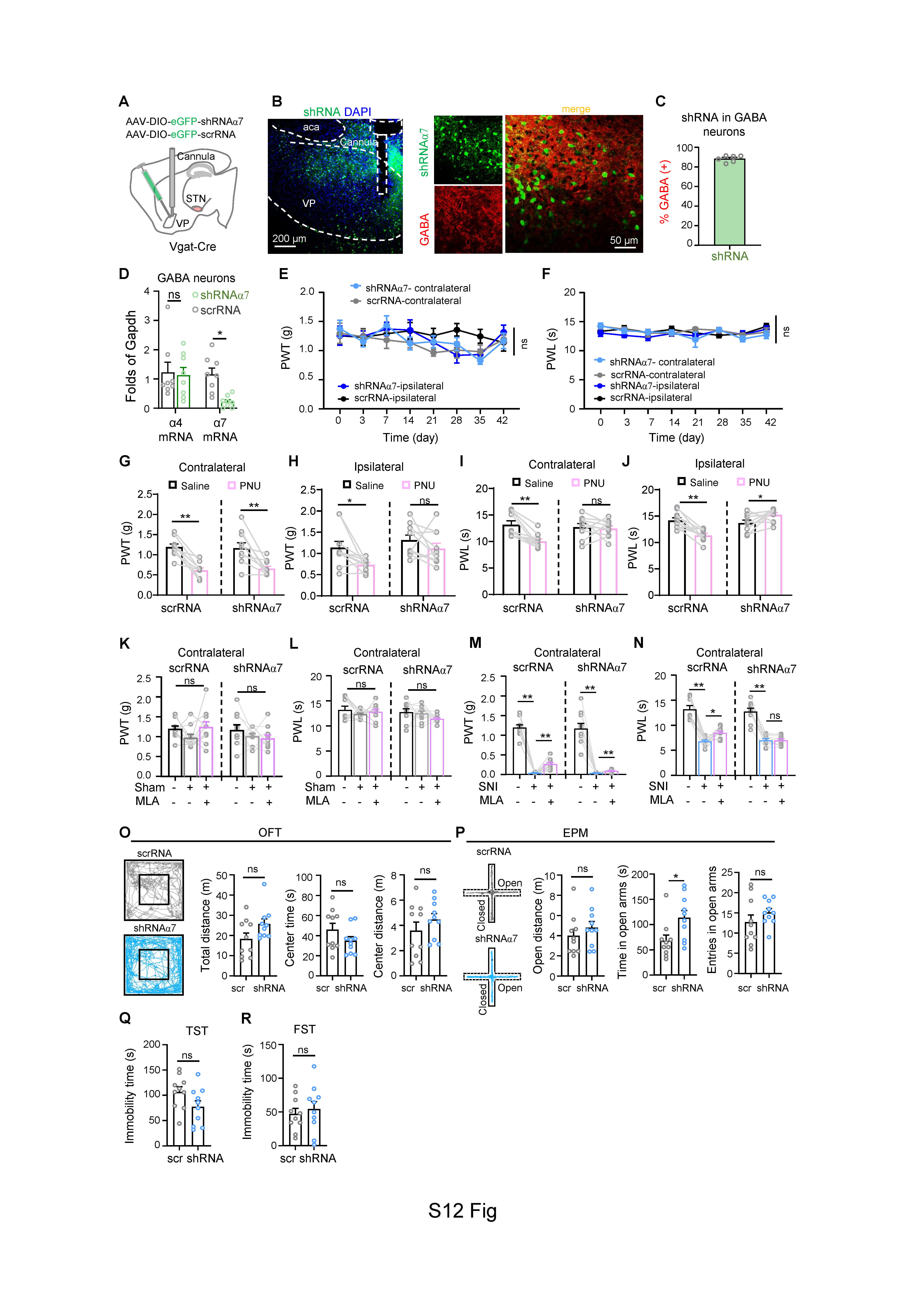

Supplement: S12 Fig — (A) Schematic diagram for injection of AAV-CMV-DIO-EGFP-shRNA(Chrna7) (shRNA) or AAV-CMV-DIO-eGFP-shRNA(scramble) (scrRNA) and implantation of a cannula into the right VP of Vgat-Cre mice. (B, C) Representative images and summary showing eGFP expression (green) in GABAergic neurons (red) 3 weeks after virus injection. GABA and shRNA co-expression neurons accounted for 88.35% of shRNA neurons. Summary data were from 6 slices of 3 mice. (D) The levels of α4 and α7 mRNA in VP GABAergic neurons in shRNAa7 and scrRNA mice were quantified with qRT-PCR assay. α4: t = 0.27, P = 0.78; α7: t = 2.68, P = 0.024, scrRNA (n = 8) vs. shRNA (n = 8). (E, F) Time courses of PWT and PWL on either hind paw in mice subjected to injection of AAV-CMV-DIO-shRNA-α7-eGFP (shRNAa7) or AAV-CMV-DIO-scramble RNA-eGFP (scrRNA) into the VP of Vgat-Cre mice. (E) PWT. Interaction, F(21, 252) = 0.87, P = 0.63; Group, F(3, 36) =1.61, P = 0.2; Time, F(6.006, 216.2) = 2.98, P = 0.008. (F) PWL. Interaction, F(21, 252) = 1.04, P = 0.41; Group, F(3, 36) = 1.15, P = 0.34; Time, F(4.426, 159.3) = 2.12, P = 0.073. (G–J) PWT and PWL on either hind paw before and after microinjection of an α7 nAChR agonist, PNU-282987, into the VP of shRNAa7 (n = 10) and scrRNA (n = 10) mice. (G) Contralateral PWT. scrRNA: t = 5.39, P < 0.001, saline vs. PNU. shRNAa7: t = 4.77, P < 0.001, saline vs. PNU. (H) Ipsilateral PWT. scrRNA: t = 2.57, P = 0.038, saline vs. PNU. shRNAa7: t = 1.27, P = 0.21, saline vs. PNU. (I) Contralateral PWL. scrRNA: t = 4.38, P < 0.001, saline vs. PNU. shRNAa7: t = 0.38, P = 0.7, saline vs. PNU. (J) Ipsilateral PWL. scrRNA: t = 4.41, P < 0.001, saline vs. PNU. shRNAa7: t = 2.38, P = 0.02, saline vs. PNU. (K–N) PWT and PWL in scrRNA (n = 10) and shRNAa7 (n = 10) mice before and after injection of MLA into the contralateral VP in sham or SNI mice. (K) PWT in sham mice. scrRNA: F(1.606, 14.45) = 1.76, P = 0.21. shRNAa7: F(1.868, 16.82) = 1.64, P = 0.22. (L) PWL in sham mice. scrRNA: F(1.761, 15.85) = 0. [file pbio.3003923.s012.tif]

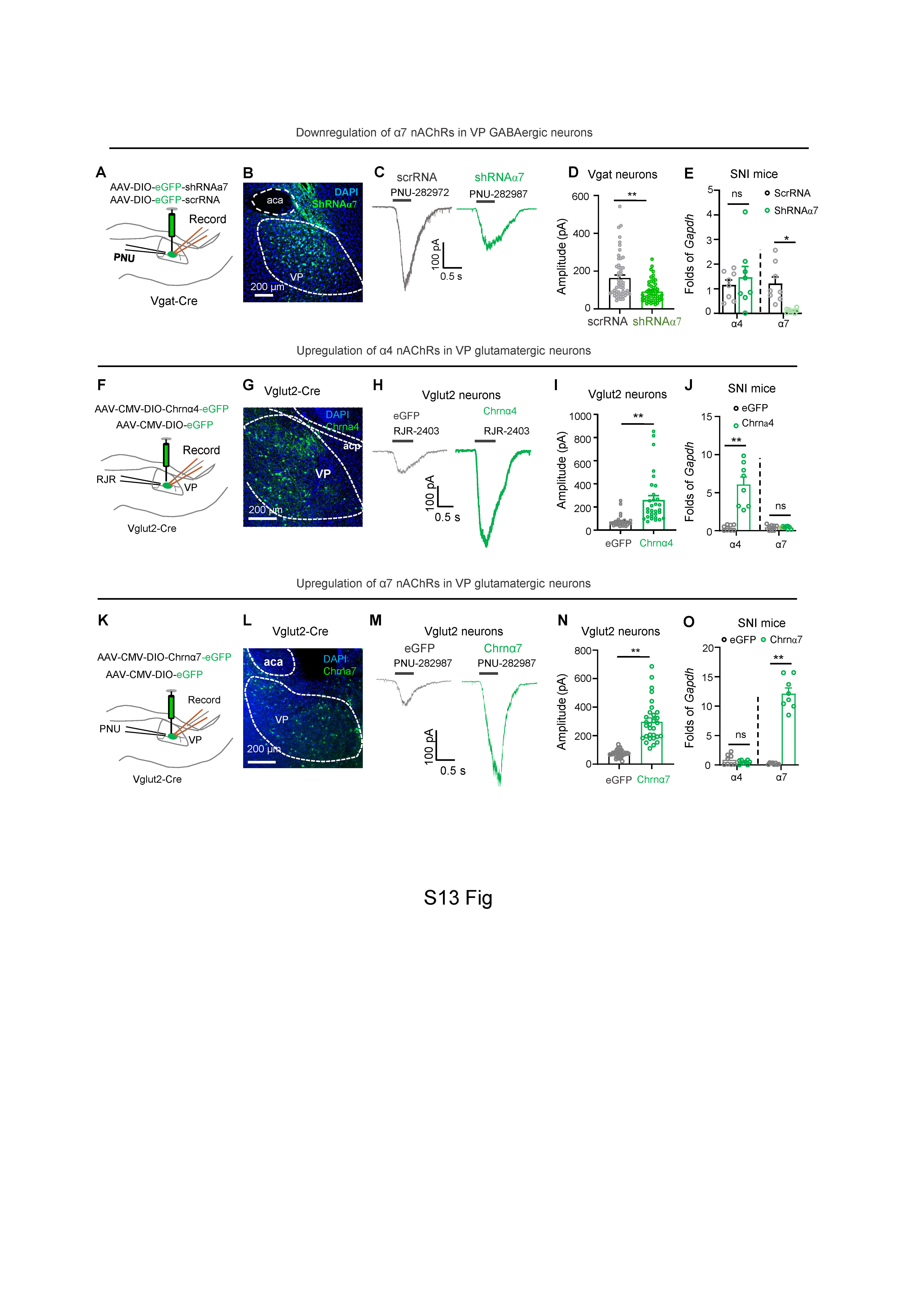

Supplement: S13 Fig — (A, B) Schematic diagram for injection of viral vector carrying shrna7 gene into the VP of Vgat-Cre mice and patch-clamp recording of puff of PNU-282987-evoked nAChR currents on glutamatergic neurons. (C, D) Representative traces and summary showing downregulation of α7 nAChR-mediated currents in 25 GABAergic neurons from 5 Chrna7 mice relative to those in 25 neurons from 5 scrRNA mice. t = 4.16, P < 0.001. (E) Quantification of mRNA levels of nAChR α4 and α7 subunit on VP glutamatergic neurons from shrna7 (n = 4) and scrRNA (n = 4) mice (2 samples from each mouse). α4: t = 0.78, P = 0.44, scrRNA vs. shrna7; α7: t = 2.75, P = 0.02, scrRNA vs. shrna7. (F, G) Schematic diagram for injection of viral vector carrying Chrna4 gene into the VP of Vglut2-Cre mice and patch-clamp recording of puff of RJR-2403-evoked nAChR currents on glutamateregic neurons. (H, I) Representative traces and summary showing upregulation of α4 nAChR-mediated currents in 31 neurons from 5 Chrna4 mice relative to those in 31 neurons from 5 eGFP mice. t = 4.71, P < 0.001. (J) Quantification of mRNA levels of nAChR α4 and α7 subunit on VP glutamatergic neurons from Chrna4 (n = 8) and eGFP (n = 8) mice. Interaction: F(1, 28) = 31.40, P < 0.001; Row Factor: F(1, 28) = 30.45, P < 0.001; Column Factor: F(1, 28) = 31.31, P < 0.001. α4: t = 7.92, P < 0.001, eGFP vs. Chrna4; α7: t = 0.005, P > 0.99, eGFP vs. Chrna4. (K, L) Schematic diagram for injection of viral vector carrying Chrna7 gene into the VP of Vglut2-Cre mice and patch-clamp recording of puff of RJR-2403-evoked nAChR currents on glutamatergic neurons. (M, N) Representative traces and summary showing upregulation of α4 nAChR-mediated currents in 30 neurons from 5 Chrna7 mice relative to those in 30 neurons from 5 eGFP mice. t = 8.47, P < 0.001. (O) Quantification of mRNA levels of nAChR α4 and α7 subunit on VP glutamatergic neurons from Chrna7 (n = 8) and eGFP (n = 8) mice. Interaction: F(1, 28) = 150.7, P < 0.001; Row Factor F(1, 28) = 120.0, [file pbio.3003923.s013.tif]

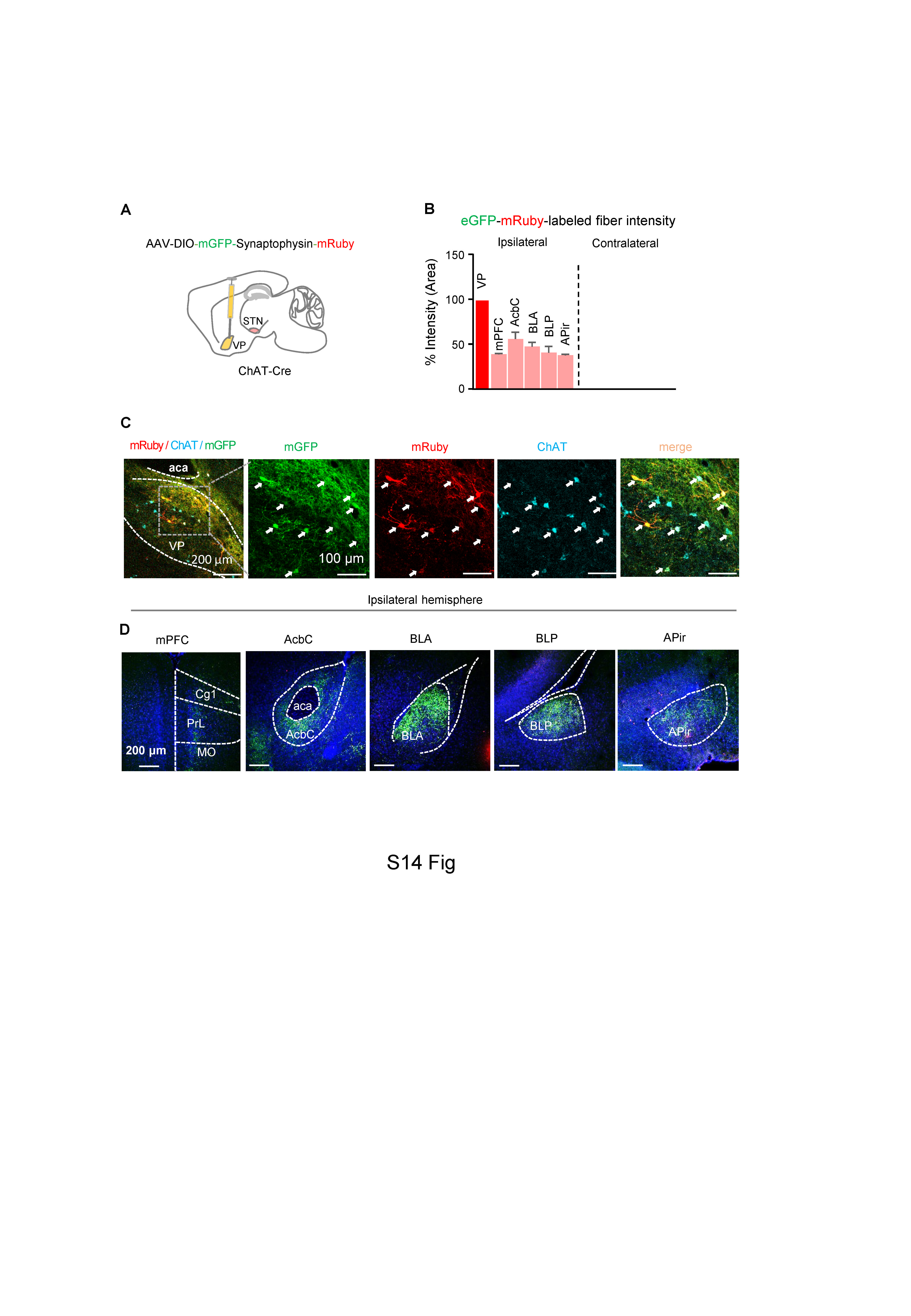

Supplement: S14 Fig — (A) Schematic diagram for viral vector-assisted anterograde tracing of VP cholinergic neurons by injecting AAV-EF1α-DIO-mGFP-Synaptophysin-mRuby into the VP of ChAT-Cre mice. (C) The VP was labeled with mGFP, mRuby, and ChAT-antibody (cyan). (B, D) Representative images and summary of downstream nuclei of VP cholinergic neurons. Summary data were from 3 slices of 3 mice. Data are available in S1 Data as a part of Supporting information. Acbc: the core of the nucleus accumbens: APir: anterior piriform cortex; BLA: basolateral amygdala; BLP: basolateral amygdala posterior part; mPFC: medial prefrontal cortex. (TIF) [file pbio.3003923.s014.tif]

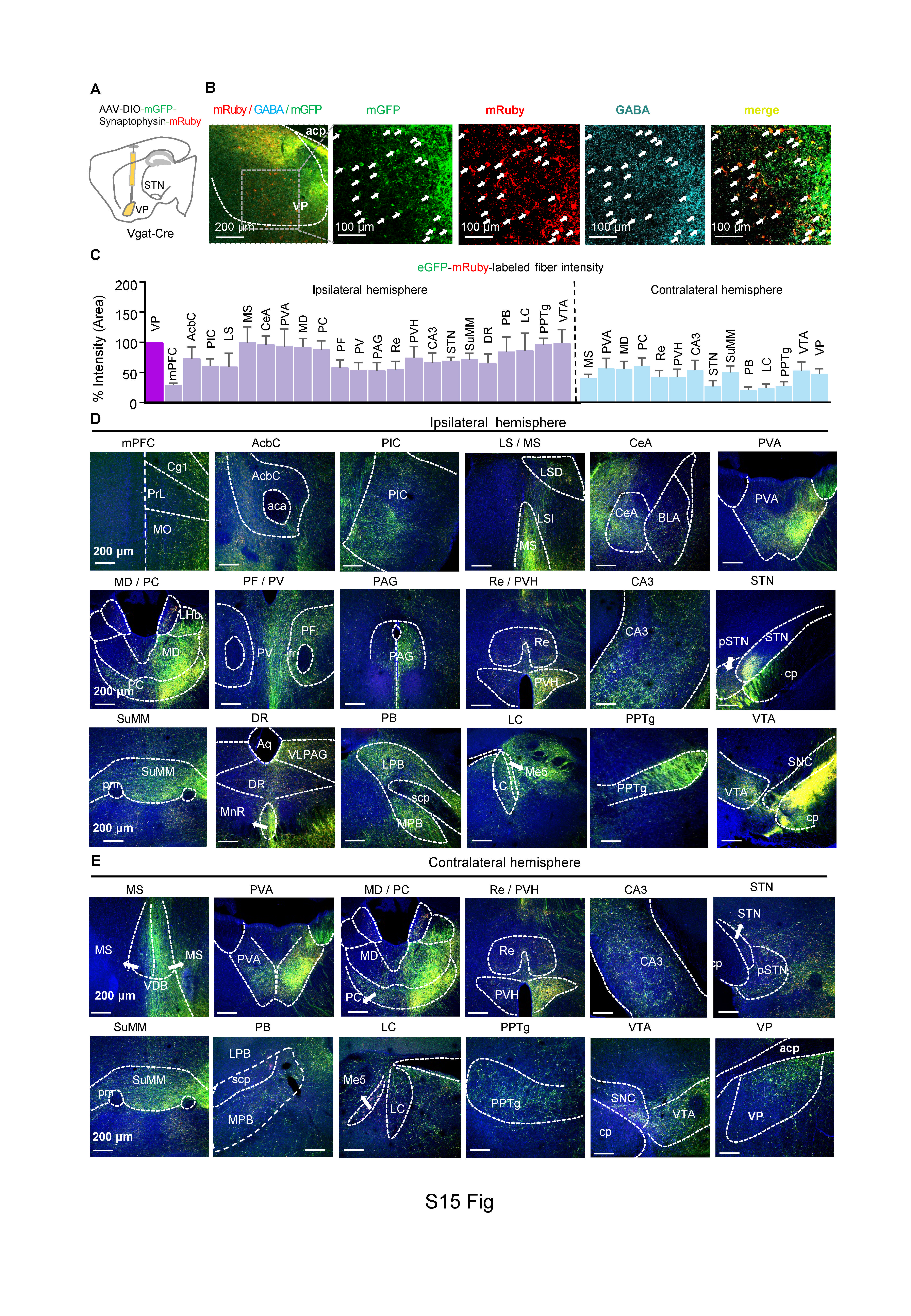

Supplement: S15 Fig — (A) Schematic diagram for viral vector-assisted anterograde tracing of VP GABAergic neurons by injecting AAV-EF1α-DIO-mGFP-Synaptophysin-mRuby into the VP of Vgat-Cre mice. (B) VP neurons were labeled with mGFP, mRuby, and GABA-antibody (cyan). (C–E) Summary and representative images showing the ipsilateral and contralateral downstream nuclei of VP GABAergic neurons. Summary data were from 3 slices of 3 mice. Fluorescence intensity was normalized to that of the whole brain. Data are available in S1 Data as a part of Supporting information. ACb: nucleus accumbens; ACC: anterior cingulate cortex; AMV/LD: anterior medioventral/ lateral dorsal nuclei of the thalamus; CeA: central amygdala; DR: dorsal raphe; LC: locus coerulus; LS: lateral septum; MD: medial dorsal nucleus of the thalamus; mPFC: medial prefrontal cortex; MS/LS: medial/ lateral septum; PAG: periaqueductal gray; PB: parabrachial nucleus; PF: parafascicular nucleus of the thalamus; PIC: posterior insular cortex; PVA: paraventricular area in the hypothalamus; Re: reunion nucleus of the thalamus; SNc: substantia nigra pars compacta; STN: subthalamic nucleus; SuMM: supramammillary nucleus, medial part; VTA: ventral tegmental area. Fluorescence intensity was normalized to that of the whole brain. (TIF) [file pbio.3003923.s015.tif]

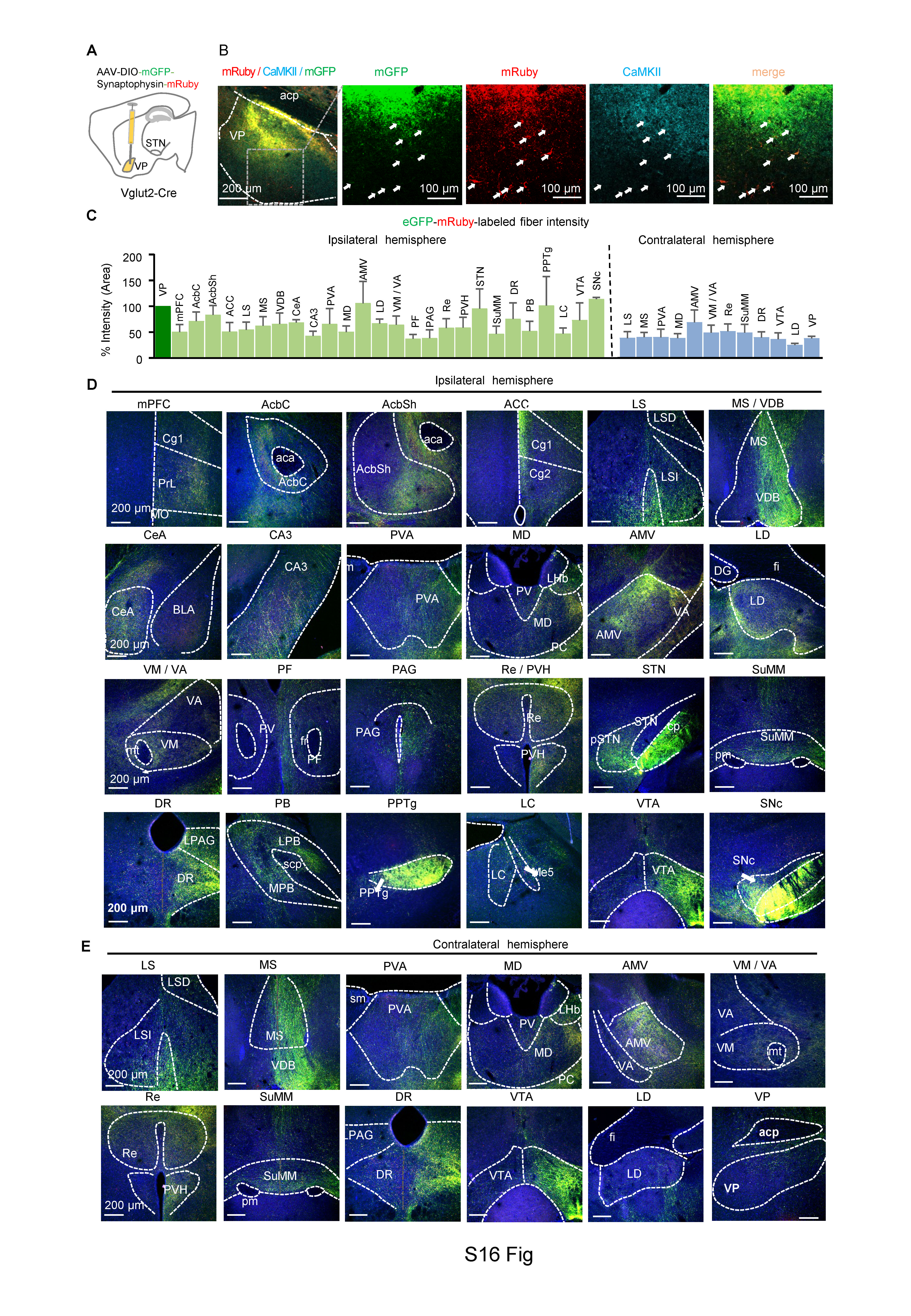

Supplement: S16 Fig — (A) Schematic diagram for viral vector-assisted anterograde tracing of VP glutamatergic neurons by injecting AAV-EF1α-DIO-mGFP-Synaptophysin-mRuby into the VP of CaMKII-Cre mice. (B) The VP was labeled with mGFP, mRuby and CaMKII-antibody (cyan). (C–E) Summary and representative images showing the ipsilateral and contralateral downstream nuclei of VP glutamatergic neurons. Summary data were from 3 slices of 3 mice. Fluorescence intensity was normalized to that of the whole brain. Data are available in S1 Data as a part of Supporting information. ACb: nucleus accumbens; ACC: anterior cingulate cortex; AMV/LD: anterior medioventral/ lateral dorsal nuclei of the thalamus; CeA: central amygdala; DR: dorsal raphe; LC: locus coerulus; MD: medial dorsal nucleus of the thalamus; mPFC: medial prefrontal cortex; MS/LS: medial/ lateral septum; PAG: periaqueductal gray; PB: parabrachial nucleus; PF: parafascicular nucleus of the thalamus; PVA: paraventricular area in the hypothalamus; Re: reunion nucleus of the thalamus; SNc: substantia nigra pars compacta; STN: subthalamic nucleus; SuMM: supramammillary nucleus, medial part; VTA: ventral tegmental area. Fluorescence intensity was normalized to that of the whole brain. (TIF) [file pbio.3003923.s016.tif]

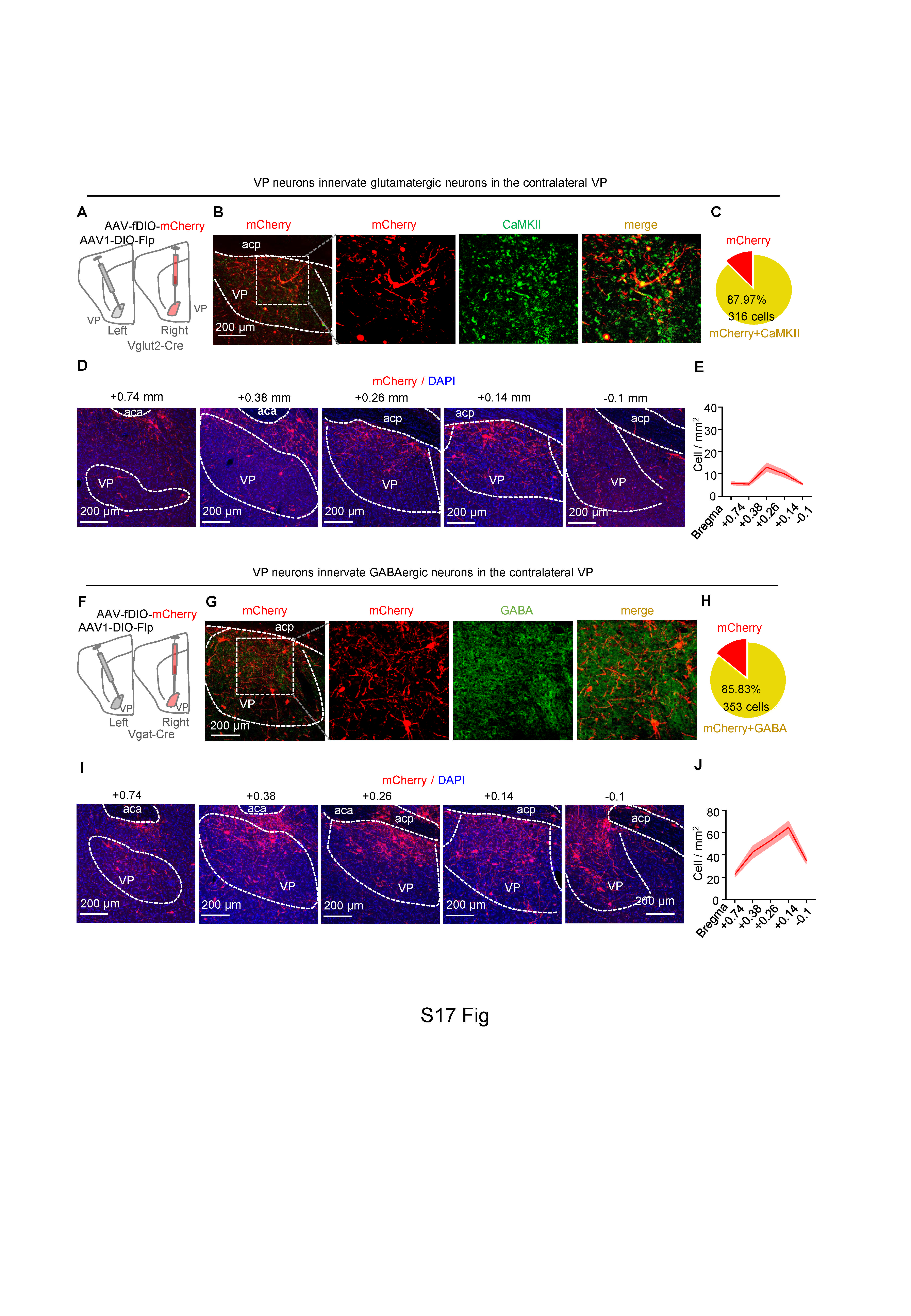

Supplement: S17 Fig — (A) Schematic diagrams for microinjection of transsynaptic anterograde viral vector (AAV1/2-DIO-Flp) into the left VP and AAV-hSyn-fDIO-mCherry into the right VP of Vglut2-Cre mice. (B) Representative images showing overlapping of mCherry(+) and CaMKII-antibody-stained neurons. (C) Representative images showing the overlapping of mCherry(+) neurons and CaMKII(+) neurons. Summary data were from 6 slices of 3 mice. (D, E) Representative images and summary showing the distribution of mCherry(+) neurons in the right VP. Summary data were from 9 slices of 3 mice. (F) Schematic diagrams for microinjection of transsynaptic anterograde viral vector (AAV1/2-DIO-Flp) into the left VP and AAV-hSyn-fDIO-mCherry into the right VP of Vgat-Cre mice. (G) Representative images showing overlapping of mCherry(+) and GABA(+) neurons. (H) Representative images showing the overlapping of mCherry(+) neurons and GABA(+) neurons. Summary data were from 8 slices of 4 mice. (I, J) Representative images and summary showing the distribution of mCherry(+) neurons in the right VP. Summary data were from 12 slices of 3 mice. Data are available in S1 Data as a part of Supporting information. (TIF) [file pbio.3003923.s017.tif]

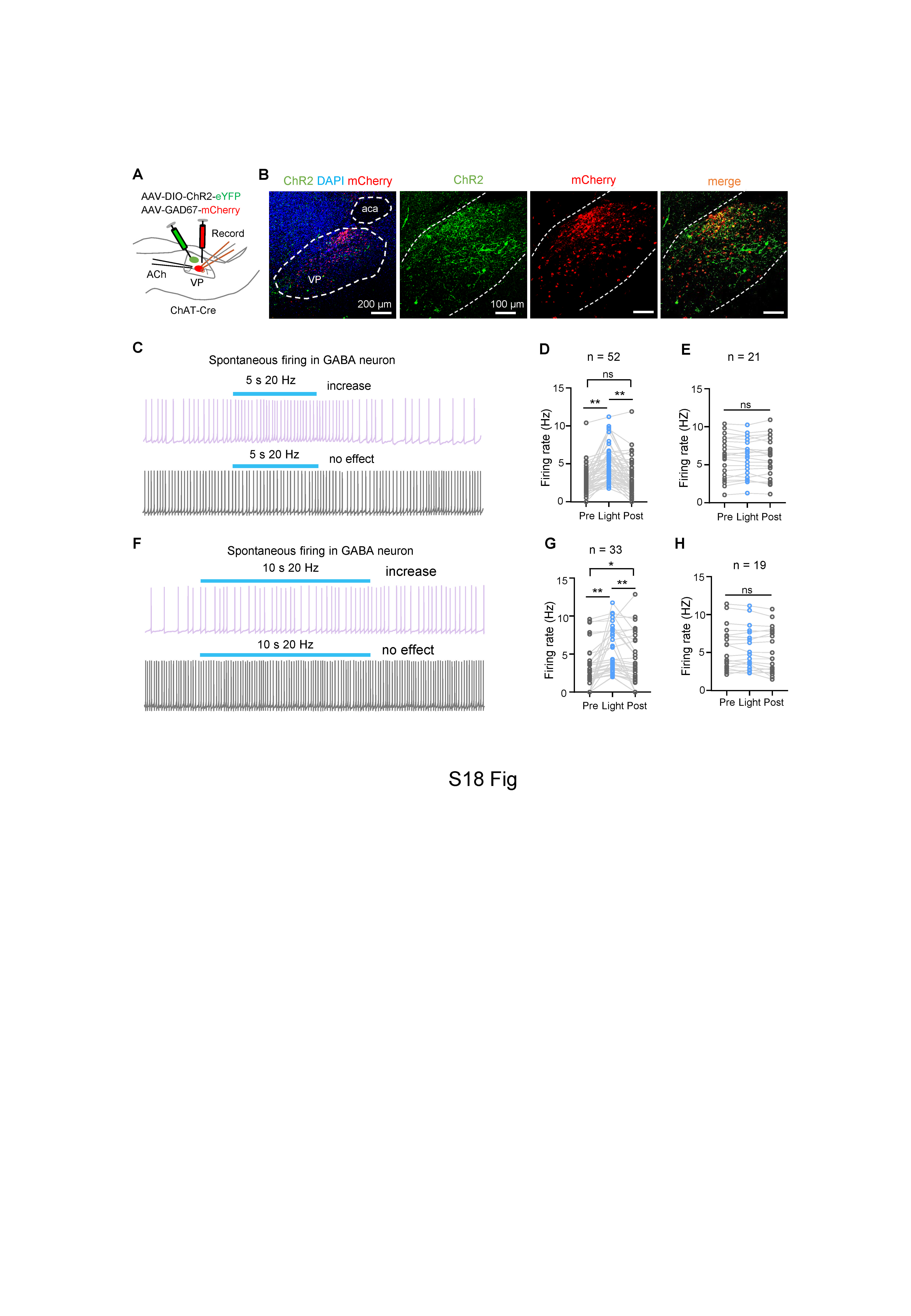

Supplement: S18 Fig — (A) Schematic diagram images for injection of AAV-DIO-ChR2-eYFP and AAVA-GAD67-mCherry into the VP of ChAT-Cre mice. (B) Representative images showing eYFP- (green) and mCherry- (red) labeled neurons in the VP 3 weeks after virus injection. (C–E) Representative traces and summary of firing in VP GABAergic neurons before, during, and after 5 s 20 Hz optogenetic stimulation of VP cholinergic neurons. (D) Increase firing. F(1.696, 86.51) = 41.31, P < 0.001, n = 52; t = 7.35, P < 0.001, before vs. during light; t = 6.88, P < 0.001, during vs. after light. (E) No effect. F(1.756, 35.12) = 0.14, n = 21, P = 0.85. (F–H) Representative traces and summary of firing in VP GABAergic neurons before, during, and after 10 s 20 Hz optogenetic stimulation of VP cholinergic neurons. (G) Increase firing. F(1.629, 52.12) = 14.34, n = 33, P < 0.001; t = 4.39, P < 0.001, before vs. after light; t = 3.31, P = 0.003, during s. after light; t = 2.69, P = 0.01, before vs. after light. (H) No effect. F(1.376, 24.77) = 2.39, n = 19, P = 0.13. * P < 0.05, ** P < 0.01, ns not significant. One-way repeated-measures ANOVAs for (D, E, G, H). Data are available in S1 Data as a part of Supporting information. (TIF) [file pbio.3003923.s018.tif]

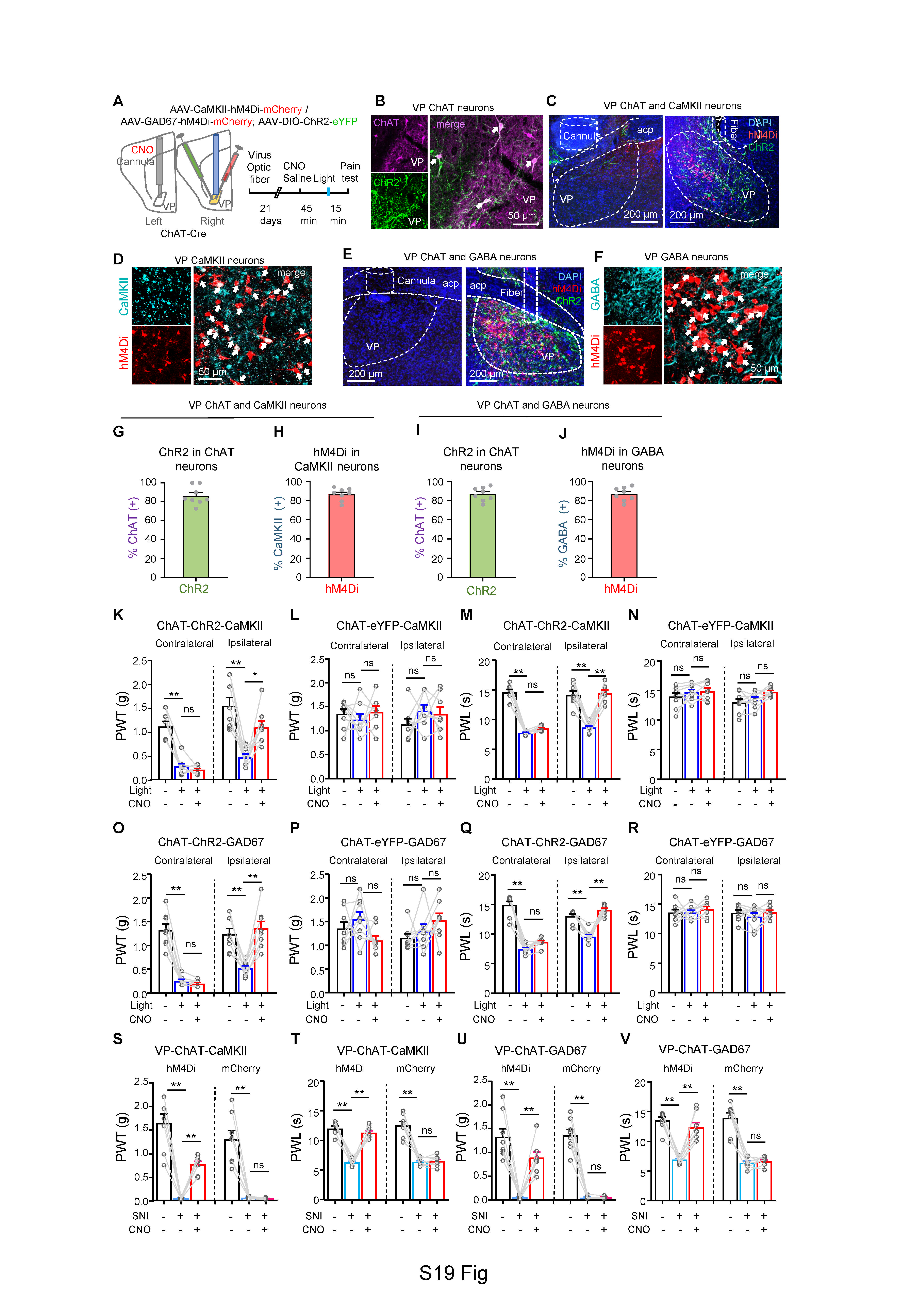

Supplement: S19 Fig — (A) Schematic diagram and timeline for optogenetic stimulation of VP cholinergic neurons and chemogenetic inhibition of contralateral projections from VP glutamatergic or GABAergic neurons. AAV-EF1α-DIO-ChR2-eYFP/AAV-EF1α-DIO-eYFP and AAV-CaMKII-hM4Di-mCherry/ AAV-GAD67-hM4Di-mCherry was injected into the right VP, and an optical fiber and a cannula were respectively implanted into the right VP and left VP. (B–F) Representative images showing ChR2-eYFP labeled cholinergic neurons in the VP (B), locations of cannula above the left VP (C, left panel) for chemogenetic inhibition of CaMKII-hM4Di-mCherry-labeled glutamatergic terminals and optical fiber in the right VP (C, right panel) for optogenetic stimulation of cholinergic neurons, transfection of hM4Di-mCherry in CaMKII neurons in the right VP (D). (E) Representative images showing locations of cannula above the left VP (left panel) for chemogenetic inhibition of GAD67-hM4Di-mCherry-labeled GABAergic terminals and optical fiber in the right VP (right panel) for optogenetic stimulation of cholinergic neurons. (F) Representative images showing transfection of GAD67-hM4Di-mCherry in GABAergic neurons in the right VP. (G, H) The specificity of viral vectors for labeling VP cholinergic neurons with ChR2-eYFP and VP CaMKII neurons with hM4Di-mCherry. (G) 86.28% of neurons transfected with ChR2 were cholinergic neurons. (H) 86.54% of neurons transfected with hM4Di were CaMKII neurons. (I, J) The specificity of viral vectors for labeling VP cholinergic neurons with ChR2-eYFP and VP GABAergic neurons with hM4Di-mCherry. (I) 83.89% of neurons transfected with ChR2 were cholinergic neurons. (J) 86.69% of neurons transfected with hM4Di were GABAergic neurons. Summary data were from 8 slices of 4 mice in each experiment. (K–N) PWT and PWL on either hind paw before and after blue light illumination in ChR2 and eYFP mice when saline or CNO was injected into the contralateral VP to inhibit hM4Di-labeled glutamatergic inputs. (K) P [file pbio.3003923.s019.tif]

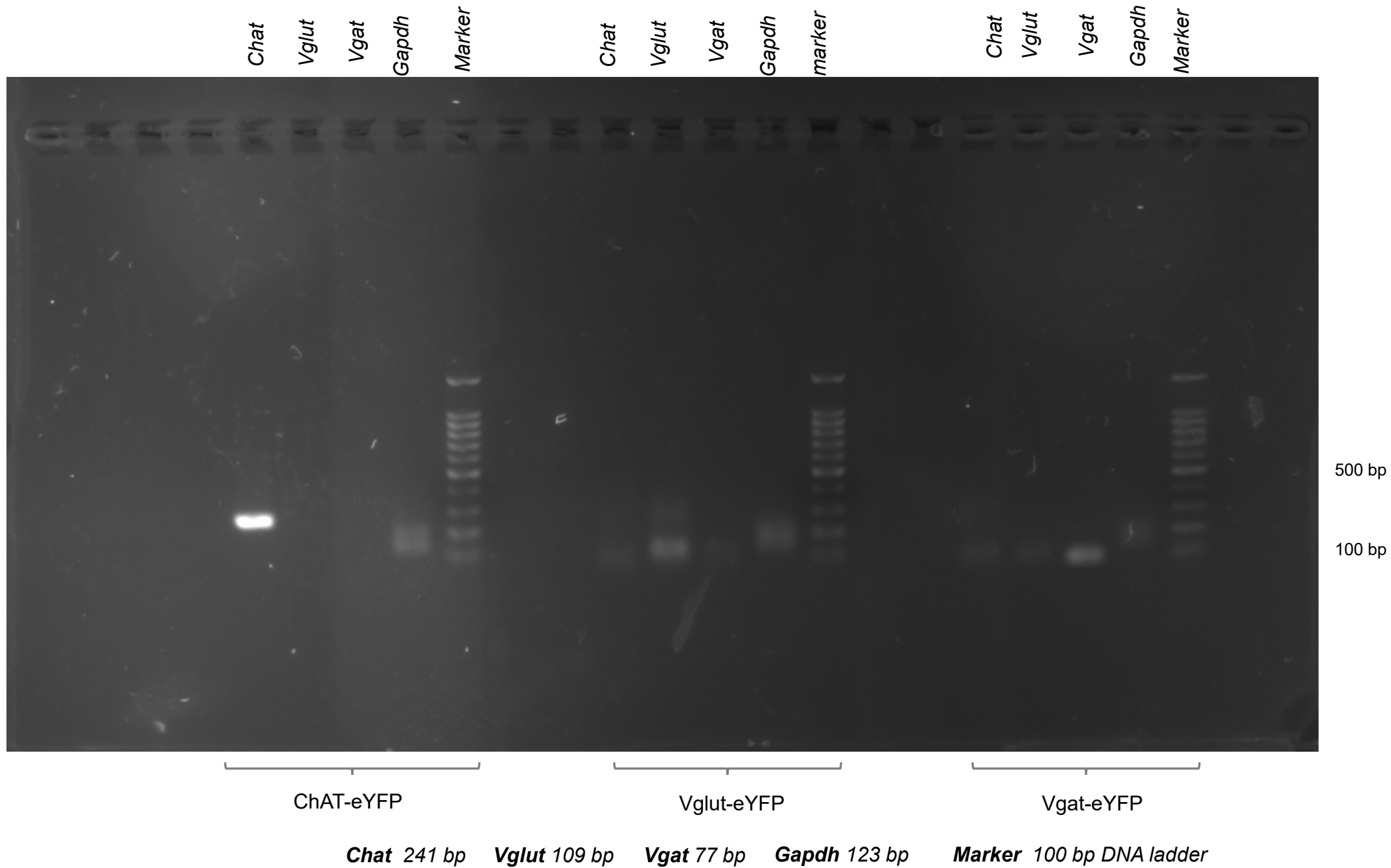

Supplement: S1 Raw Images — (PDF) [file pbio.3003923.s022.pdf]
