# Supplementary material for: The subthalamic nucleus-ventral pallidum projection targeting cholinergic circuits modulates chronic pain
Source: PLoS Biol. 2026 Aug 3;24(8):e3003923. doi: 10.1371/journal.pbio.3003923 (PMC13432146; doi:10.1371/journal.pbio.3003923)
Supplement: S1 Table — (DOCX) [file pbio.3003923.s020.docx]

**S1 Table**. Primer sequences for genes measured in qRT-PCR analysis

| gene | Forward primer | Reverse primer |
| --- | --- | --- |
| *α2* | 5`ACTCCATCCCAGGCTAGTGA3` | 5`TCCCCAGGAAGCAGACGATA3` |
| *α3* | 5`GCACAGAATGTAGCCAAAGAGATTC3` | 5`TTGGCGAACAGGTCACAGTT3` |
| *α4* | 5`tgcgctgggaccctggtgactac3` | 5`tccccgtccgcgttgttgtagag3` |
| *α5* | 5`AGAAGCCGAGAAAGACGGTG3` | 5`TCTTCAACAACCTCGCGGA3` |
| *α6* | 5`AAAGGCAGTACAGGCTGTGAA3` | 5`CGCCGACAGCATTGTTATAC3` |
| *α7* | 5`TGCCACATTCCACACCAACG3` | 5`CTACGGCGCATGGTTACTGT3` |
| *β2* | 5`TGCGAAGTGAAGATGATGACCAG3` | 5`ACATGCCAATGGTCCCAAAGA3` |
| *β3* | 5`AGTTCTGGTCGCTTTCCTGG3` | 5`TCCAGTCTTGAACAACCTGACT3` |
| *β4* | 5`TACAACAACCTGATCCGCCC3` | 5`CATAGGTCCCATCGGCATTG3` |
| *Chat* | 5`AGGGCAGCCTCTCTGTATGA3` | 5`ATCCTCGTTGGACGCCATTT3` |
| *Gapdh* | 5`AGGTCGGTGTGAACGGATTTG3` | 5`TGTAGACCATGTAGTTGAGGTCA3` |
| *M1* | 5`TCCCTCACATCCTCCGAAGGTG3` | 5`CTTTCTTGGTGGGCCTCTTGACTG3` |
| *M2* | 5`CTGGAGCACAACAAGATCCAGAAT3` | 5`CCCCCTGAACGCAGTTTTCAGT3` |
| *M3* | 5`GCAAGACCTCTGACACCAACT3` | 5`AGCAAACCTCTTAGCCAGCG3` |
| *M4* | 5`CGGCTACTGGCTCTGCTACGTCAA3` | 5`CTGTGCCGATGTTCCGATACTGG3` |
| *M5* | 5`TAGCATGGCTGGTCTCCTTCA3` | 5`CGCTTCCCGACCAAGTACTG3` |
| *Vgat* | 5`GGGTCACGACAAACCCAAGA3` | 5`GCACGAACATGCCCTGAATG3` |
| *Vglut2* | 5` GCGGAGGCAAAGTTATCAAG3` | 5`CCTGGAATCTGGGTGATGAT3` |
